# How Occam’s razor guides human decision-making

**DOI:** 10.1101/2023.01.10.523479

**Published:** 2023-01-10

**Authors:** Eugenio Piasini, Shuze Liu, Pratik Chaudhari, Vijay Balasubramanian, Joshua I. Gold

**Affiliations:** 1 International School for Advanced Studies (SISSA), Trieste, Italy; 2 University of Pennsylvania, Philadelphia, PA, USA; 3 Santa Fe Institute, Santa Fe, NM, USA

## Abstract

Occam’s razor is the principle that, all else being equal, simpler explanations should be preferred over more complex ones^1^. This principle is thought to play a role in human perception and decision-making^2^, but the nature of our presumed preference for simplicity is not understood. Here we use preregistered behavioral experiments informed by formal theories of statistical model selection^3^ to show that, when faced with uncertain evidence, human subjects exhibit preferences for particular, theoretically grounded forms of simplicity of the alternative explanations. These forms of simplicity can be understood in terms of geometrical features of statistical models treated as manifolds in the space of the probability distributions, in particular their dimensionality, boundaries, volume, and curvature. The simplicity preferences driven by these features, which are also exhibited by artificial neural networks trained to optimize performance on comparable tasks, generally improve decision accuracy, because they minimize over-sensitivity to noisy observations (i.e., overfitting). However, unlike for artificial networks, for human subjects these preferences persist even when they are maladaptive with respect to the task training and instructions. Thus, these preferences are not simply transient optimizations for particular task conditions but rather a more general feature of human decision-making. Taken together, our results imply that principled notions of statistical model complexity have direct, quantitative relevance to human and machine decision-making and establish a new understanding of the computational foundations, and behavioral benefits, of our predilection for inferring simplicity in the latent properties of our complex world.

## Occam’s razor formalized as model selection

**Figure 1:**
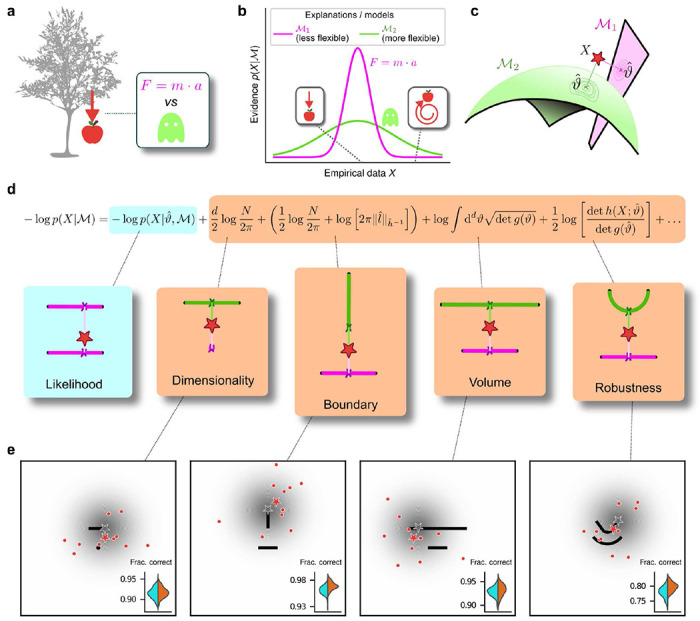
Formalizing Occam’s razor as Bayesian model selection to understand simplicity preferences in human decision-making. ***a:*** Occam’s razor prescribes an aversion to complex explanations (models). In Bayesian model selection, model complexity is a measure of the flexibility of a model, or its capacity to account for a broad range of empirical observations. In this example, we observe an apple falling from a tree (left) and compare two possible explanations: 1) classical mechanics, and 2) the intervention of a ghost. ***b:*** Schematic comparison of the evidence of the two models in ***a:*** Classical mechanics (pink) explains a narrower range of observations than the ghost (green), which is a valid explanation for essentially any conceivable phenomenon (e.g., both a falling and spinning-upward trajectory, as in the insets). Absent further evidence, Occam’s razor posits that the simpler model (classical mechanics) is preferred, because its hypothesis space is more concentrated around the sparse, noisy data and thus avoids “overfitting” to noise. ***c:*** A geometrical view of the model-selection problem. Two alternative models are represented as geometrical manifolds, and the maximum-likelihood point $\hat{\vartheta }$ for each model is represented as the projection of the data (red star) onto the manifolds. ***d:*** Systematic expansion of the log evidence of a model M. $\hat{\vartheta }$ is the maximum-likelihood point on model M for data X, N is the number of observations, d is the number of parameters of the model, $\hat{l}$ is the likelihood gradient evaluated at $\hat{\vartheta }$, h is the observed Fisher information matrix, and g is the expected Fisher information matrix (see Methods). g(ϑ) captures how distinguishable elements of M are in the neighborhood of ϑ. When M is the true source of the data X, h(X;ϑ) can be seen as a noisy version of g(ϑ), estimated from limited data. ${\hat{h}}^{-1}$ is a shorthand for $h{\left(X;\hat{\vartheta }\right)}^{-1}$, and ${\left\|\hat{l}\right\|}_{{\hat{h}}^{-1}}=\sqrt{{\hat{l}}^{T}\left({\hat{h}}^{-1}\right)\hat{l}}$ is the length of $\hat{l}$ measured in the metric defined by ${\hat{h}}^{-1}$. The ellipsis collects terms that decrease as N grows. Each term of the expansion represents a distinct geometrical feature of the model^3^: dimensionality penalizes models with many parameters; boundary (a novel contribution of this work) penalizes models for which $\hat{\vartheta }$ is on the boundary; volume counts the number of distinguishable probability distributions contained in M; and robustness captures the shape (curvature) of M near $\hat{\vartheta }$. **e:** Psychophysical task with variants designed to probe each geometrical feature in **d**. For each trial, a random location on one model was selected (gray star), and data (red dots) were sampled from a Gaussian centered around that point (gray shading). The red star represents the empirical centroid of the data, by analogy with **c**. The maximum-likelihood point can be found by projecting the empirical centroid onto one of the models. Subjects saw the models (black lines) and data (red dots) only and were required to choose which model was best for the data. Insets: task performance for the given task variant, for a set of 100 simulated ideal Bayesian observers (orange) versus a set of 100 simulated maximum-likelihood observers (i.e., choosing based only on whichever model was the closest to the empirical centroid of the data on a given trial; cyan).

To make decisions in the real world, we must often choose between multiple, plausible explanations for noisy, sparse data. When evaluating such competing explanations, Occam’s razor says that we should consider not just how well they account for the observed data, but also their potentially excessive flexibility in describing alternative, and potentially irrelevant, data that have not been observed (e.g., “a ghost did it!”, Figure 1a). In cognitive science, simplicity, or parsimony, has long been proposed as an organizing principle in mental function^2^, from the early concept of Prágnanz in Gestalt psychology^4^, to a number of “minimum principles” for vision^5^, to theories that posit a central role for data compression in cognition^6^. However, despite evidence that human decision-makers can exhibit simplicity preferences under certain task conditions^7–11^, we lack a principled understanding of what, exactly, constitutes the “simplicity” that is favored (or, equivalently, “complexity” that is disfavored) and how we balance that preference with the evidence provided by the observed data when we make decisions.

To provide this understanding, we turn to an approach based on Bayesian statistics^7^, which allows us to measure the complexity of an explanation for data on an absolute scale. Our process is formalized as a model-selection problem: given a set *X* of *N* observations and a set of possible statistical models {*M*_*1*_, *M*_*2*_, …}, we seek the model *M* that in some sense is the best for the data *X*. In this context, Occam’s razor can be interpreted as requiring the goodness-of-fit of a model to be penalized by some measure of its flexibility, or complexity, when comparing it against other models. Bayesian statistics offers a natural characterization of such a measure of complexity and specifies the way in which it should be traded off against goodness-of-fit to maximize decision accuracy, typically because the increased flexibility provided by increased complexity tends to cause errors by overfitting to noise in the observations^12–14^.

Specifically, according to this framework models should be compared based on their evidence or marginal likelihood p(X|M)=∫dϑw(ϑ)p(X|M,ϑ), where ϑ represents model parameters and *w*(ϑ) their associated prior (Figure 1b). Under mild regularity assumptions and with sufficient data, the (log) evidence can be written as the sum of the maximum log likelihood of *M* and several penalty factors (Figure 1d). These penalty factors, which are found even when the prior probabilities of the models under consideration are equal (i.e., independent of the data, all are equally likely to be the correct model), can be interpreted as providing quantitatively defined preferences against certain models according to specific forms of complexity that they embody^3,14^. If the prior over parameters *w*(ϑ) is taken to be uninformative^15^, each penalty factor can be shown to capture a distinct geometric property of the model^3^, including dimensionality (number of parameters), boundary (a novel term, detailed below), volume, and shape (Figure 1c,d). This approach, which we call the Fisher Information Approximation (FIA), generalizes the well-known Bayesian Information Criterion (BIC) for model selection^16,17^. Its effectiveness has been demonstrated by using it to identify worse-fitting, but better-generalizing, psychophysical models describing the relationship between physical variables (e.g., light intensity) and their psychological counterparts (e.g., brightness)^18^. Similar quantitative definitions of statistical model complexity or model-selection prescriptions can be obtained with different theoretical approaches, such as the Minimum Description Length^19–21^, Minimum Message Length^22^, and Predictive Information^23^ frameworks, testifying to the generality of this approach.

A limitation of these existing approaches is that they typically assume that the maximum-likelihood solution is in the interior of the parameter space of a given model^3^. In contrast, because models are just approximations of the true processes in the real world that generated a given set of observations, those observations may fall outside of the parameter space of a given model. In these cases (or even when the observations are based on samples generated by the model but are corrupted by noise to fall outside of the model’s parameter space), the maximum-likelihood solution for that model, given those data, may fall on the boundary of the model’s parameter space. To account for this condition, we extended the FIA to deal with the simple case of a linear boundary in parameter space (see Methods). When the maximum-likelihood solution is on such a boundary, an additional penalty term appears in the FIA, which we denote “boundary” (Figure 1d). This extended FIA, consisting of dimensionality, boundary, volume, and robustness terms, provides a quantitative framework for assessing simplicity preferences in simple decision tasks, as we detail below.

## Humans exhibit theoretically grounded simplicity preferences

We designed a simple decision-making task to relate the FIA complexity terms to the potential preferences exhibited by both human and artificial decision-makers. For each trial, *N*=10 simultaneously presented observations (red dots in Figure 1e) were sampled from a 2D Normal (“generative”) distribution centered somewhere within one of two possible shapes (black shapes in Figure 1e). The identity of the shape generating the data (top versus bottom) was chosen at random with equal probability. Likewise, the location of the center of the Normal distribution within the selected shape was sampled uniformly at random, in a way that did not depend on the model parametrization, by using Jeffrey’s prior^15^. Given the observations, the subjects decided which shape (model) was more likely to contain the center of the generative distribution. We used four task variants, each designed to primarily probe one of the distinct geometrical features that are penalized in Bayesian model selection (i.e., a Bayesian observer is expected to have a particular, quantitative preference away from the more-complex alternative in each pair; Figure 1d and e). In our task, the FIA provided a good approximation of the exact Bayesian posterior (Supplementary Information section S.1 and Extended Data Figure E.1).

For our human studies, we used the on-line research platforms Pavlovia, to implement the task, and Prolific, to recruit subjects. Following our preregistered approaches^24–26^, we collected data from 202 subjects, divided into four groups that each performed one of the four separate versions of the task depicted in Figure 1e (each group comprised ~50 subjects). We provided instructions that used the analogy of seeds from a flower located in one of two flowerbeds, to provide an intuitive framing of the key concepts of noisy data generated by a particular instance of a parametric model from one of two model families. To minimize the possibility that subjects would simply learn from implicit or explicit feedback over the course of each session to make more optimal (i.e., simplicity-preferring) choices of flowerbeds, we: 1) used conditions for which the difference in performance between ideal observers that penalized model complexity according to the FIA and simulated observers that used only model likelihood was ~1% (depending on the task type; Figure 1e, insets), which translates to ~5 additional correct trials over the course of an entire experiment; and 2) provided feedback only at the end of each block of 100 trials, not each trial. We used hierarchical (Bayesian) logistic regression to measure the degree to which each subject’s choices were affected by model likelihood (distance from the data to a given model) and each of the FIA-derived geometrical features characterizing model complexity (see Methods, section M.5). We defined each subject’s sensitivity to each FIA term as a normalized quantity, relative to their likelihood sensitivity (i.e., by dividing the logistic coefficient associated with a given FIA term by the logistic coefficient associated with the likelihood).

**Figure 2:**
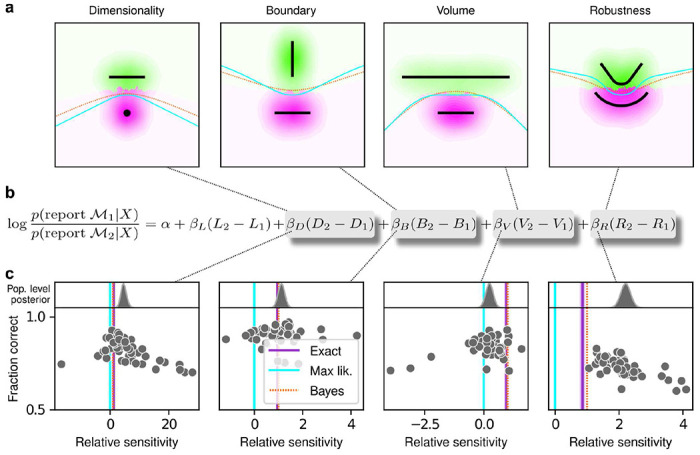
Humans exhibit theoretically grounded simplicity preferences **a**: Summary of human behavior. Hue (pink/green): k-nearest-neighbor interpolation of the model choice, as a function of the empirical centroid of the data. Color gradient (light/dark): marginal density of empirical data centroids for the given model pair, showing the region of space where data were more likely to fall. Cyan solid line: decision boundary for an observer that always chooses the model with highest maximum likelihood. Orange dashed line: decision boundary for an ideal Bayesian observer. The subjects’ choices tended to reflect a preference for the simpler model, particularly near the center of the screen, where the evidence for the alternatives was weak. For instance, in the left panel there is a region where data were closer to the line than to the dot, but subjects chose the dot (the simpler, lower-dimensional “model”) more often than the line. **b:** Subject sensitivity to each geometrical feature characterizing model complexity was estimated via hierarchical logistic regression (see Methods, section M.5, Supplementary Information section S.2 and Extended Data Figure E.2), using as predictors a constant to account for an up/down choice bias, the difference in likelihoods for the two models (L_2_–L_1_) and the difference in each FIA term for the two models (D_2_–D_1_, etc). Following a hierarchical regression scheme, the subject-level sensitivities were in turn modeled as being sampled from a population-level distribution. The mean of this distribution is our population-level estimate for the sensitivity. **c:** Overall accuracy versus estimated relative FIA sensitivity for each task condition, as indicated. Points are data from individual subjects. Each fitted FIA coefficient was normalized to the likelihood coefficient and thus could be interpreted as a relative sensitivity to the associated FIA term. For each term, an ideal Bayesian observer would have a relative sensitivity of one (dashed orange lines), whereas an observer that relied on only maximum-likelihood estimation (i.e., choosing “up” or “down” based on only the model that was the closest to the data) would have a relative sensitivity of zero (solid cyan lines). Top, gray: Population-level estimates (posterior distribution of population-level relative sensitivity given the experimental observations). Bottom: each gray dot represents the task accuracy of one subject (y axis) versus the posterior mean estimate of the relative sensitivity for that subject (x axis). Purple: relative sensitivity of an ideal observer that samples from the exact Bayesian posterior (not the approximated one provided by the FIA). Shading: posterior mean ± 1 or 2 stdev., estimated by simulating 50 such observers.

The human subjects were sensitive to all four forms of model complexity (Figure 2). Specifically, the estimated normalized population-level sensitivity for human subjects (posterior mean ± st. dev., where zero implies no sensitivity and one implies Bayes-optimal sensitivity) was 4.66±0.96 for dimensionality, 1.12±0.10 for boundary, 0.23±0.12 for volume, and 2.21±0.12 for robustness (note that, following our preregistered plan, we emphasize parameter estimation using Bayesian approaches^27–29^ here and throughout the main text, and we provide complementary null hypothesis significance testing in the Supplementary Information, Section S.6 and Extended Data Table E.8). Formal model comparison (WAIC; see Supplementary Information, section S.6.1 and Extended Data Tables E.6 and E.7) confirmed that their behavior was better described by taking into account the geometric penalties defined by the theory of Bayesian model selection, rather than by relying on only the minimum distance between model and data (i.e., the maximum-likelihood solution).

The subjects also exhibited substantial individual variability in performance that included ranges of sensitivities to each FIA term that spanned optimal and sub-optimal values. This variability was large compared to the uncertainty associated with subject-level sensitivity estimates (Supplementary Information, section S.4 and Extended Data Figure E.4) and impacted performance in a manner that highlighted the usefulness of appropriately tuned (i.e., close to Bayes optimal) simplicity preferences: accuracy tended to decline for subjects with FIA sensitivities further away from the theoretical predictions (Figure 2c; posterior mean ± st. dev. of Spearman’s *rho* between accuracy and |β-1|, where β is the sensitivity: dimensionality, −0.69±0.05; boundary, −0.21±0.11; volume, −0.10±0.10; robustness, −0.54±0.10). The sub-optimal sensitivities exhibited by many subjects did not appear to result simply from a lack of task engagement, because FIA sensitivity did not correlate with errors on easy trials (posterior mean ± st. dev. of Spearman’s *rho* between lapse rate, estimated with an extended regression model detailed in Methods, section M.5.1, and the absolute difference from optimal sensitivity for: dimensionality, 0.08±0.12; boundary, 0.15±0.12; volume, −0.04±0.13; robustness, 0.15±0.14; see Supplementary Information section S.5 and Extended Data Figure E.5). Likewise, sub-optimal FIA sensitivity did not correlate with weaker likelihood sensitivity for the boundary (*rho*=−0.13±0.11) and volume (−0.06±0.11) terms, although stronger, negative relationships with the dimensionality (−0.35±0.07) and robustness terms (−0.56±0.10) suggest that the more extreme and variable simplicity preferences under those conditions (and lower performance, on average; see Figure 2c) reflected a more general difficulty in performing those versions of the task.

## Human simplicity preferences are robust to task demands

**Figure 3:**
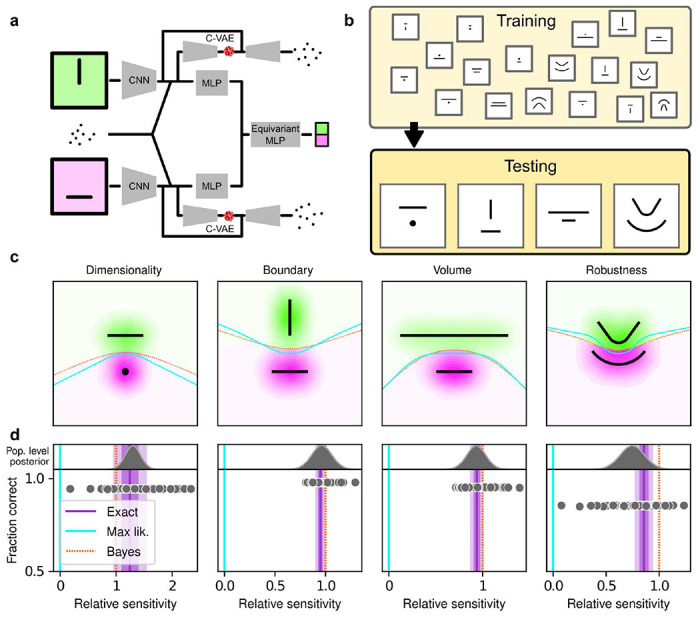
Deep neural networks exhibit theoretically grounded simplicity preferences **a**: A novel deep neural-network architecture for statistical model selection. The network (see text and Methods for details) takes two images as input, each representing a model, and a set of 2D coordinates, each representing a datapoint. The output is a softmax-encoded choice between the two models. **b:** Each network was trained on multiple variants of the task, including systematically varied model length or curvature, then tested using the same configurations as for the human studies. **c:** Summary of network behavior, like Figure 2a. Hue (pink/green): k-nearest-neighbor interpolation of the model choice, as a function of the empirical centroid of the data. Color gradient (light/dark): marginal density of empirical data centroids for the given model pair, showing the region of space where data were more likely to fall. Cyan solid line: decision boundary for an observer that always chooses the model with highest maximum likelihood. Orange dashed line: decision boundary for an ideal Bayesian observer. **d**: Estimated relative sensitivity to geometrical features characterizing model complexity. As for the human subjects, each fitted FIA coefficient was normalized to the likelihood coefficient and thus could be interpreted as a relative sensitivity to the associated FIA term. For each term, an ideal Bayesian observer would have a relative sensitivity of one (dashed orange lines), whereas an observer that relied on only maximum-likelihood estimation (i.e., choosing “up” or “down” based on only the model that was the closest to the data) would have a relative sensitivity of zero (solid cyan lines). Top: population-level estimate (posterior distribution of population-level relative sensitivity given the experimental observations; see Methods, section M.5 for details). Bottom: each gray dot represents the task accuracy of one of 50 trained networks (y axis) versus the posterior mean estimate of the relative sensitivity for that network (x axis). Purple: relative sensitivity of an ideal observer that samples from the exact Bayesian posterior (not the approximated one provided by the FIA). Shading: posterior mean ± 1 or 2 stdev., estimated by simulating 50 such observers.

To better understand the optimality, variability, and generality of the simplicity preferences exhibited by our human subjects, we compared their performance to that of artificial neural networks (ANNs) trained to optimize performance on this task. We used a novel ANN architecture that we designed to perform statistical model selection, in a form applicable to the task described above (Figure 3a,b). On each trial, the network took as input two images representing the models to be compared, and a set of coordinates representing the observations on that trial. The output of the network was a decision between the two models, encoded as a softmax vector. We analyzed 50 instances of the ANN that differed only in the random initialization of their weights and in the examples seen during training, using the same logistic-regression approach we used for the human subjects.

The ANN was designed as follows. The input stage consisted of two pretrained VGG16 convolutional neural networks (CNNs), each of which took in a pictorial representation of one of the two models under consideration. VGG was chosen as a popular architecture that is often taken as a benchmark for comparisons with the human visual system^30,31^. The CNNs were composed by a number of convolutional layers, whose weights were kept frozen at their pretrained values, followed by three fully-connected layers, whose weights were allowed to change during training (see Methods for details). The output of the CNNs were each fed into a multilayer perceptron (MLP) consisting of linear, rectified-linear (ReLU), and batch-normalization layers. The MLP outputs were then concatenated and fed into an Equivariant MLP, which enforces equivariance of the network output under position swap of the two models through a custom parameter-sharing scheme^32^. The network also contained two conditional variational autoencoder (C-VAE) structures, which sought to replicate the data-generation process conditioned on each model and therefore encouraged the fully connected layers upstream to learn model representations that captured task-relevant features.

After training, the ANNs performed the task substantially better than the human subjects, with higher overall accuracies that included higher likelihood sensitivities (Supplementary Information, section S.3 and Extended Data Table E.3) and simplicity preferences that more closely matched the theoretically optimal values (Figure 3d). In fact, these simplicity preferences were closer to those expected from simulated observers that use the exact Bayesian model posterior rather than the FIA-approximated one, indicating an imperfect approximation of the FIA to the exact Bayesian posterior rather than suboptimal network behavior. These simplicity preferences varied slightly in magnitude across the different networks, but unlike for the human subjects this variability was relatively small (compare ranges of values in Figures 2c and 3d, plotted on different x-axis scales) and not related systematically to any differences in the generally high accuracy rates for each condition (Figure 3e; posterior mean ± st. dev. of Spearman’s *rho* between accuracy and |β−1|, where β is the sensitivity: dimensionality, −0.14±0.10; boundary, 0.08±0.11; volume, −0.12±0.11; robustness, −0.08±0.11). These results imply that the stochastic nature of the task gives rise to some variability in simplicity biases even after extensive training to optimize performance accuracy, but this source of variability cannot by itself account for the range of sensitivities (and suboptimalities) exhibited by the human subjects.

These results, combined with the fact that we did not provide trial-by-trial feedback to the subjects while they performed the task, suggest that the human simplicity preferences we measured were not simply learned optimizations for these particular task conditions but rather are a more inherent (and variable) part of how we make decisions under uncertainty. However, because we provided each subject with instructions that echoed Bayesian-like reasoning (see Methods) and a brief training set with feedback before their testing session, we cannot rule out from this dataset alone that at least some aspects of the simplicity preferences we measured from the human subjects depended on those specific instructions and training conditions. We therefore ran a second experiment to rule out this possibility. For this experiment, we used the same task variants as above but a different set of instructions and training, designed to encourage subjects to pick the model with the maximum likelihood, thus disregarding model complexity. Specifically, the visual cues were the same as in the original experiment, but the subjects were asked to report which of the two shapes on the screen was closest to the center-of-mass of the dot cloud. We ensured that the subjects recruited for this “maximum-likelihood” task had not participated in the original, “generative” task. We also trained and tested ANNs on this version of the task, using the maximum-likelihood solution as the correct answer.

**Figure 4:**
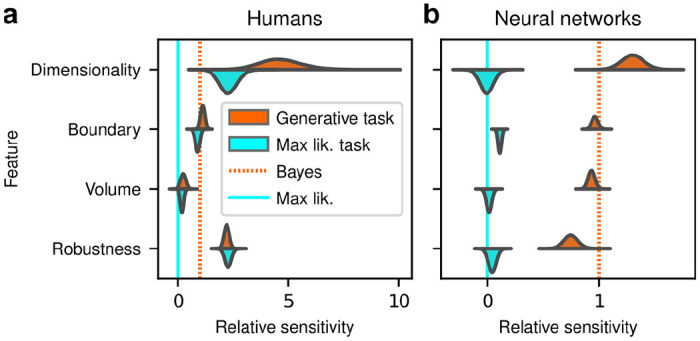
Humans, but not artificial networks, exhibit simplicity preferences even when they are suboptimal **a**: Relative sensitivity of human subjects to the geometric complexity terms (population-level estimates, as in Figure 2c, top) for two task conditions: 1) the original, “generative” task where subjects were implicitly instructed to solve a model-selection problem (same data as in Figure 2c, top; cyan); and 2) a “maximum-likelihood” task variant, where subjects were instructed to report which of two models has the highest likelihood (shortest distance from the data; orange). The two task variants were tested on distinct subject pools of roughly the same size (202 subjects for the generative task, 201 for the maximum-likelihood task, in both cases divided in four groups of roughly 50 subjects each). Solid cyan lines: relative sensitivity of a maximum-likelihood observer. Orange dashed lines: relative sensitivity of an ideal Bayesian observer. **b**: Same comparison and format, but for two distinct populations of 50 deep neural networks trained on the two variants of the task (orange is the same data as in Figure 3d, top).

Despite this major difference in instructions and training, the human subjects exhibited similar simplicity preferences on the generative and maximum-likelihood tasks, suggesting that humans have a general predilection for simplicity even without relevant instructions or incentives (Figure 4, left). Specifically, despite some quantitative differences, the distributions of relative sensitivities showed the same basic patterns for both tasks, with a general increase of relative sensitivity from volume (0.19±0.08 for the maximum-likelihood task; compare to values above), to boundary (0.89±0.10), to robustness (2.27±0.15), to dimensionality (2.29±0.41). In stark contrast to the human data and to ANNs trained on the true generative task, ANN sensitivity to model complexity on the maximum-likelihood task was close to zero for all four terms (Figure 4, right).

**Figure 5:**
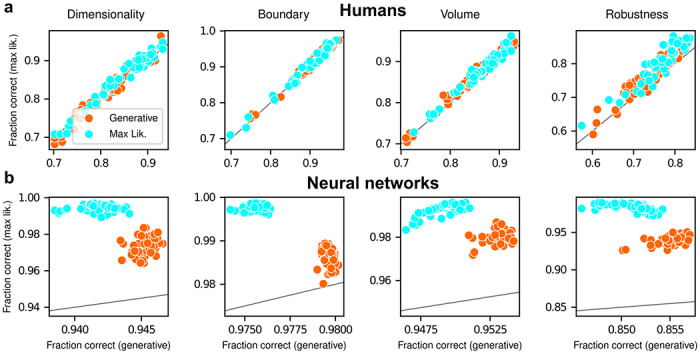
Humans and artificial networks have different patterns of accuracy reflecting their different use of simplicity preferences Each panel shows accuracy with respect to maximum-likelihood solutions (i.e., the model closest to the centroid of the data; ordinate) versus with respect to generative solutions (i.e., the model that generated the data; abscissa). The gray line is the identity. Columns correspond to the four task variants associated with the four geometric complexity terms, as indicated. **a**: Data from individual human subjects (points), instructed to find the generative (orange) or maximum-likelihood (cyan) solution. Subject performance was higher when evaluated against maximum-likelihood solutions than it was when evaluated against generative solutions, for all groups of subjects (two-tailed paired t-test, generative task subjects: dimensionality, t-statistic 2.21, p-value 0.03; boundary, 6.21, 1e-7; volume, 9.57, 8e-13; robustness, 10.6, 2e-14. Maximum-likelihood task subjects: dimensionality, 5.75, 5e-7; boundary, 4.79, 2e-6; volume, 10.8, 2e-14; robustness, 12.2, 2e-16). **b:** Data from individual ANNs (points), trained on the generative (orange) or maximum-likelihood (cyan) task. Network performance was always highest when evaluated against maximum-likelihood solutions, compared to generative solutions (all dots are above the identity line).

To summarize the similarities and differences between how humans and ANNs used simplicity biases to guide their decision-making behaviors for these tasks, and their implications for performance, Figure 5 shows overall accuracy for each set of conditions we tested. Specifically, for each network or subject, task configuration, and instruction set, we computed the percentage of correct responses with respect to both the generative task (i.e., for which theoretically optimal performance depends on simplicity biases) and the maximum-likelihood task (i.e., for which theoretically optimal performance does not depend on simplicity biases). Because the maximum-likelihood solutions are deterministic (they depend only on which model the data centroid is closest to, and thus there exists an optimal, sharp decision boundary that leads to perfect performance) and the generative solutions are not (they depend probabilistically on the likelihood and bias terms, so it is generally impossible to achieve perfect performance), performance on the former is expected to be higher than on the latter. Accordingly, both ANNs and (to a lesser extent) humans tended to perform better when assessed relative to maximum-likelihood solutions. Moreover, the ANNs tended to exhibit behavior that was consistent with optimization to the given task conditions: networks trained to find maximum-likelihood solutions did better than networks trained to find generative solutions for the maximum-likelihood task, and networks trained to find generative solutions did better than networks trained to find maximum-likelihood solutions for the generative task. In contrast, humans tended to adopt similar strategies regardless of the task conditions, in all cases using Bayesian-like simplicity biases.

Put briefly, ANNs exhibited simplicity preferences only when trained to do so, whereas human subjects exhibited them regardless of their instructions and training.

## Discussion

Simplicity has long been regarded as a key element of effective reasoning and rational decision-making, and it has been proposed as a foundational principle in philosophy^1^, psychology^2,6^, statistical inference^3,12–14,20,22,33,34^, and more recently machine learning^35–38^. Accordingly, multiple studies have identified preferences for simplicity in human cognition^7,9,10^, such as a tendency to prefer smoother (simpler) curves as the inferred, latent source of noisy observed data^8,11^. However, the quantitative form and magnitude of this preference have never been identified. In this work, we showed that the simplicity preference is closely related to a specific mathematical formulation of Occam’s razor, situated at the convergence of Bayesian model selection and information theory^3^. This formulation enabled us to go beyond the mere detection of a preference for simple explanations for data and to measure precisely the strength of this preference in artificial and human subjects under a variety of theoretically motivated conditions.

Our study makes several novel contributions. The first is theoretical: we derived a new term of the Fisher Information Approximation (FIA) in Bayesian model selection that accounts for the possibility that the best model is on the boundary of the model family. This boundary term is important because it can account for the possibility that, because of the noise in the data, the best value of one parameter (or of a combination of parameters) takes on an extreme value. This condition is related to the phenomenon of “parameter evaporation” that is common in real-world models for data^39^. Moreover, boundaries for parameters are particularly important for studies of perceptual decision-making, in which sensory stimuli are limited by the physical constraints of the experimental setup and thus reasoning about unbounded parameters would be problematic for subjects. For example, imagine designing an experiment that requires subjects to report the location of a visual stimulus. In this case, an unbounded set of possible locations (e.g., along a line that stretches infinitely far in the distance to the left and to the right) is clearly untenable. Our “boundary” term formalizes the impact of considering the set of possibilities as having boundaries, which tend to increase local complexity because they tend to reduce the number of local hypotheses close to the data (see Figure 1b).

The second contribution of this work relates to ANNs: these networks can learn to use or ignore the simplicity preferences in an optimal way (i.e., according to the magnitudes prescribed by the theory), depending on how they are trained. These results are different from, and complementary to, recent work that has focused on the idea that implementation of simple functions could be key to generalization in deep neural networks^35–38^. Here we have shown that effective learning can take into account the complexity of the hypothesis space, rather than that of the decision function, in producing normative simplicity preferences. On the one hand, these results do not seem surprising, because ANNs, and deep networks in particular, are powerful function approximators that perform well in practice on a vast range of inference tasks^40^. Accordingly, our ANNs trained with respect to the true generative solutions were able to make effective decisions, including simplicity preferences, about the generative source of a given set of observations. Likewise, our ANNs trained with respect to maximum-likelihood solutions were able to make effective decisions, without simplicity preferences, about the maximum-likelihood match for a given set of observations. On the other hand, these results provide new insights into how ANNs might be analyzed to better understand the kinds of solutions they produce for particular problems. In particular, assessing the presence or absence of these kinds of simplicity preferences might help identify if and/or how well an ANN is likely to avoid overfitting to training data and provide more generalizable solutions.

The third, and most important, contribution of this work relates to human behavior: people tend to use simplicity preferences when making decisions, and unlike ANNs these preferences do not seem to be simply the consequences of learning specific task demands but rather an inherent part of how we interpret uncertain information. This tendency has important implications for the kinds of computations our brains must use to solve these kinds of tasks, and how those computations appear to differ from those implemented by the ANNs we used. From a theoretical perspective, the difference between a Bayesian solution (i.e., one that includes the simplicity preferences) and a maximum-likelihood solution (i.e., one that does not include the simplicity preferences) to these tasks is that the latter considers only the single best-fitting model from each family, whereas the former integrates over all possible models in each family. Our finding that ANNs can converge on either solution when trained appropriately indicates that both are, in principle, learnable. However, our finding that people tend to use the Bayesian solution even when instructed to use the maximum-likelihood solution suggests that we naturally do not make decisions based simply on the single best or archetypical instance within a family of possibilities but rather integrate across that family. Put more concretely in terms of our task, when told to identify the shape closest to the data points, subjects were likely uncertain about which exact location on each shape was closest and thus integrated over the possibilities – thus inducing simplicity preferences as prescribed by the Bayesian solution. These findings will help motivate and inform future studies to identify where and how the brain implements and stores these integrated solutions to relevant decision problems.

Another key feature of our findings that merits further study is the magnitude and variability of preferences exhibited by the human subjects. On average, human sensitivity to each geometrical model feature was: 1) larger than zero, 2) at least slightly different from the optimal value (e.g., larger for dimensionality and robustness, smaller for volume), 3) different for distinct features and different subjects; and 4) independent of instructions and training. What is the source of this diversity? One hypothesis is that people may weigh more heavily the model features that are easier or cheaper to compute. In our experiments, the most heavily weighted feature was model dimensionality. In our mathematical framework, this feature corresponds to the number of degrees of freedom of a possible explanation for the observed data and thus can be relatively easy to assess. By contrast, the least heavily weighted feature was model volume. This feature involves integrating over the whole model family (to count how many distinct states of the world can be explained by a certain hypothesis, one needs to enumerate them) and thus can be very difficult to compute. The other two terms, boundary and robustness, are intermediate in terms of human weighting and computational difficulty: they are harder to compute than dimensionality, because they depend on the data and on the properties of the model at the maximum likelihood location, but are also simpler than the volume term, because they are local quantities that do not require integration over the whole model manifold. This intuition leads to new questions about the relationship between the complexity of the explanations being compared and the complexity of the decision-making process itself, calling into question notions of bounded rationality and diminishing returns in optimal inference^41,42^. Answering such questions is beyond the scope of the present work but merits further study.

Another potentially intriguing future direction is a comparison with other formal approaches to the emergence of simplicity that can lead to different predictions. Recent studies have argued that Jeffrey’s prior (upon which our geometric approach is based) could give an incomplete picture of the complexity of a class of models that occur commonly in the natural sciences, which contain many combinations of parameters that do not affect model behavior, and proposed instead the use of data-dependent priors^43,44^. The two methods lead to different results, especially in the data-limited regime^45^. It would be useful to understand the relevance of these differences to human and machine decision-making.

In summary, our work reveals the direct, quantitative relevance of formal notions of model complexity for human behavior. By relying on a combination of theoretical advances, computational modeling and behavioral experiments, we have established a novel set of normative reference points for decision making under uncertainty. Our findings therefore open up a new arena in which human cognition could be measured against optimal inferential processes, potentially leading to new insights into the constraints affecting information processing in the brain.

## Supplementary Material

1

## Data Availability

All experimental data collected in this work is available at doi:10.17605/OSF.IO/R6D8N
