# Supplementary material for: How Occam’s razor guides human decision-making
Source: bioRxiv. 2025 Mar 16:2023.01.10.523479. Originally published 2023 Jan 10. Preprint. [Version 4] doi: 10.1101/2023.01.10.523479 (PMC9882019; doi:10.1101/2023.01.10.523479)
Supplement: Supplementary file 1 [file NIHPP2023.01.10.523479v4-supplement-1.pdf]

bioRxiv preprint doi: https://doi.org/10.1101/2023.01.10.523479; this version posted March 16, 2025. The copyright holder for this preprint (which was not certified by peer review) is the author/funder, who has granted bioRxiv a license to display the preprint in perpetuity. It is made available under aCC-BY 4.0 International license.

Appendix A
Methods

A.1 Noise-Integration-Noise (NIN) ideal observer

To formalize the intuition around Occam's razor emerging from a process of integration over latent causes, we define an ideal observer that can perform the model-selection tasks described in the main text based on a simple set of heuristics. The observer has three degrees of freedom: 1) the intensity of sensory noise, 2) the extent to which it performs integration over latent causes, and 3) the intensity of the choice noise. We call this the Noise-Integration-Noise (NIN) observer.

In particular, we assume that some empirical data $X = \{x_i\}_{i=1}^N$ are generated from one of the models described in section A.4. Briefly, each datapoint $x_i \in \mathbb{R}^2$ is sampled independently with

$$p(x|t) = \frac{1}{\sqrt{2\pi\sigma^2}} \exp\left[-\frac{\|x - \mu_{\mathcal{M}}(t)\|^2}{2\sigma^2}\right]$$

where σ is fixed and known (in practice, $\sigma = 1$ for our simulations), $\|\cdot\|$ is the Euclidean norm in $\mathbb{R}^2$, and the (smooth) function $\mu_{\mathcal{M}} : [0,1] \to \mathbb{R}^2$ is specific to the model $\mathcal{M}$ generating the data. For instance, in the example shown in Figure 1 in the main text (the Dimensionality task), $\mu_{\mathcal{M}}(t) = (0,0)$ for the model on the bottom (the dot) and $\mu_{\mathcal{M}}(t) = ((t-1)/2, 1)$ for the model on top (the line).

For data generated according to the procedure above, the ideal observer starts by computing the average of the empirical data

$$\bar{x} = \frac{1}{N}\sum_{i=1}^{N} x_i$$

This representation of the data is corrupted by normally distributed sensory noise of intensity ρ:

$$p(\tilde{x}|\bar{x}) = \frac{1}{\sqrt{2\pi\rho^2}} \exp\left[-\frac{(\tilde{x} - \bar{x})^2}{2\rho^2}\right]$$

Based on this (noisy) internal representation of the data, the participant computes an approximate Bayesian posterior, based on computing the evidence in a restricted neighborhood of the maximum-likelihood point:

$$p_b(X|\mathcal{M}) = \int_{\Psi(b)} \mathrm{d}t\, w(t) p(X|t)$$

where w is uniform over Ψ, and the neighborhood $\Psi(b)$ is defined as a function of the integration parameter b in such a way that the observer will:

1. perform full Bayesian integration for $b = 1$,
2. take into account only the maximum-likelihood point for $b = 0$, so that $p(X|\mathcal{M}) = p(X|\hat{t})$, and
3. will perform an operation that interpolates between these two extremes for intermediate values of b.

This behavior is obtained by setting

$$\Psi(b) = \left\{t : \mu_{\mathcal{M}}(t) \leq d(b)\right\} \quad ,$$

$$d(b) = \begin{cases} \max_t \|\tilde{x} - \mu_{\mathcal{M}}(t)\| & \text{if } b = 1 \\ \min_t \|\tilde{x} - \mu_{\mathcal{M}}(t)\| & \text{if } b = 0 \\ bd(1) + (1-b)d(0) & \text{if } 0 < b < 1 \end{cases}$$

In practice, the observer considers only the portion of the model contained within a circle of radius $d(b)$ centered on the data, with the radius set such that: 1) for $b = 0$, the circle will touch the model only at the point that is closest to the data; 2) for $b = 1$, the circle will include all of the model; and 3) for intermediate values, the circle will interpolate linearly between these two cases.

bioRxiv preprint doi: https://doi.org/10.1101/2023.01.10.523479; this version posted March 16, 2025. The copyright holder for this preprint (which was not certified by peer review) is the author/funder, who has granted bioRxiv a license to display the preprint in perpetuity. It is made available under aCC-BY 4.0 International license.

Finally, the participant picks a model sampling from the following distribution:

$$p(\mathcal{M}_1|X) = \frac{1}{1 + \exp\left[-(1/T)\left(\log p_b(X|\mathcal{M}_1) - \log p_b(X|\mathcal{M}_2)\right)\right]}$$

Where the "temperature" parameter T controls the level of choice noise. This procedure corresponds to sampling from the approximate Bayesian posterior when $T = 1$, picking the model with the highest posterior for $T \to \infty$, and picking a model at random for $T \to 0$.

A.1.1 Model-free analysis of simplicity bias for the NIN observer

We performed an elementary, model-free assessment of the simplicity bias exhibited by the NIN observer as a function of the observer's parameters. For a given configuration of the observer, we simulated 10000 trials for each of the task types described in the main text. We then quantified the choice bias by fitting a sigmoid psychometric curve (as a function of y, the vertical coordinate) to data falling in a restricted region in the center of the data space ($-0.1 < x < 0.1$, $0 < y < 1$):

$$p(\text{choose "up"}|y) = \frac{1}{1 + e^{-a(y-(0.5-b)))}}$$

where a governs the shape (slope) of the psychometric curve, and b governs the bias that here is defined as the distance between the decision boundary and the midpoint between the models ($x = 0, y = 0.5$). The sign of the bias is taken such that a positive bias pushes the decision boundary towards the more complex model (this is the direction expected from Occam's razor), and a negative bias has the opposite effect.

A.2 Derivation of the boundary term in the Fisher Information Approximation

Here we generalize the derivation of the Fisher Information Approximation (FIA) given by Balasubramanian (1997) to the case where the maximum-likelihood solution for a model lies on the boundary of the parameter space. This generalization is important because it relies on more realistic assumptions than existing approaches. In particular, existing approaches typically assume that the maximum-likelihood solution is in the interior of the parameter space of a given model. In contrast, models are just approximations of the true processes in the real world that generated a given set of observations, implying that those observations may fall outside of the range that can be expected based on the parameter space of a given model. In these cases (or even when the observations are based on samples generated by the model but are corrupted by noise to fall outside of the range implied by most values of the model's parameters space), the maximum-likelihood solution for that model, given those data, may fall on the boundary of the model's parameter space. To account for this condition, we extended the FIA to deal with the simple case of a linear boundary in parameter space. When the maximum-likelihood solution is on such a boundary, an additional penalty term appears in the FIA, which we denote "boundary" (see Figure 1d in the main text).

Apart from the more general assumptions, the following derivation follows closely the original one, with some minor notational changes. This derivation appeared in preliminary form in Piasini et al. (2021a).

Sketch of the derivation. Our derivation below uses a well-known approximation method to yield a closed-form expression for the model evidence in the limit of large sample size (large N). The evidence is the integral of the likelihood over all possible parameter values, weighted by the prior. Up to constant terms, this integral can be written in the form $\int \mathrm{d}\vartheta \exp\left[-N\psi(\vartheta, X)\right]$, where $\psi(\vartheta, X)$ is a function that depends on the data X and on a location in parameter space ϑ. In a nutshell, the approximation consists in noticing that, as N increases, the integral will come to be dominated by the behavior of the integrand within a small neighborhood of the maximum-likelihood point $\hat{\vartheta}$ in parameter space (which is itself dependent on the data: $\hat{\vartheta} = \hat{\vartheta}(X)$). In this neighborhood, we rewrite the integrand by Taylor-expanding $\psi(\vartheta, X)$ around the maximum-likelihood point. By inspecting the resulting series, we drop the terms of the expansion that decrease in magnitude when N grows, keeping only those that dominate in that regime. The end result is a Gaussian integral, which we can solve in closed form to yield the Fisher Information Approximation. This procedure is the same whether the maximum-likelihood point is in the interior of or on the boundary of the parameter space; the difference is that with a few technical passages we can show that, in the latter case, an additional term appears in the solution to the Gaussian integral. Intuitively, this extra term is due to the fact that when the maximum likelihood point $\hat{\vartheta}$ is on the boundary of the parameter space the gradient of the likelihood in $\hat{\vartheta}$ is not zero.

bioRxiv preprint doi: https://doi.org/10.1101/2023.01.10.523479; this version posted March 16, 2025. The copyright holder for this preprint (which was not certified by peer review) is the author/funder, who has granted bioRxiv a license to display the preprint in perpetuity. It is made available under aCC-BY 4.0 International license.

A.2.1 Set-up and hypotheses

The problem we consider here is that of selecting between two models (say $\mathcal{M}_1$ and $\mathcal{M}_2$), after observing empirical data $X = \{x_i\}_{i=1}^N$. N is the sample size and $\mathcal{M}_1$ is assumed to have d parameters, collectively indexed as ϑ taking values in a compact domain Θ. As a prior over ϑ we take Jeffrey's prior:

$$w(\vartheta) = \frac{\sqrt{\det g(\vartheta)}}{\int \mathrm{d}^d \vartheta \sqrt{\det g(\vartheta)}} \tag{A1}$$

where g is the (expected) Fisher Information of the model $\mathcal{M}_1$:

$$g_{\mu\nu}(\vartheta) = \mathbb{E}\left[-\frac{\partial^2 \ln p(x|\vartheta)}{\partial\vartheta^\mu \partial\vartheta^\nu}\right]_\vartheta \tag{A2}$$

The Bayesian posterior

$$\mathbb{P}(\mathcal{M}_1|X) = \frac{\mathbb{P}(\mathcal{M}_1)}{\mathbb{P}(X)} \int \mathrm{d}^d\, w(\vartheta)\mathbb{P}(X|\vartheta) \tag{A3}$$

then becomes, after assuming a flat prior over models and dropping irrelevant terms:

$$\mathbb{P}(\mathcal{M}_1|X) = \frac{\int_\Theta \mathrm{d}^d \vartheta \sqrt{\det g} \exp[-N(-\frac{1}{N} \ln \mathbb{P}(X|\vartheta))]}{\int \mathrm{d}^d \vartheta \sqrt{\det g}} \tag{A4}$$

Just as in Balasubramanian (1997), we now make a number of regularity assumptions: 1) $\ln \mathbb{P}(X|\vartheta)$ is smooth; 2) there is a unique global minimum $\hat{\vartheta}$ for $\ln \mathbb{P}(X|\vartheta)$; 3) $g_{\mu\nu}(\vartheta)$ is smooth; 4) $g_{\mu\nu}(\hat{\vartheta})$ is positive definite; 5) $\Theta \subset \mathbb{R}^d$ is compact; and 6) the values of the local minima of $\ln \mathbb{P}(X|\vartheta)$ are bounded away from the global minimum by some $\epsilon > 0$. Importantly, unlike in Balasubramanian (1997), we do not assume that $\hat{\vartheta}$ is in the interior of Θ.

The shape of Θ. Because we are interested in understanding what happens at a boundary of the parameter space, we add a further assumption that, while being not very restrictive in spirit, allows us to derive a particularly interpretable result. In particular, we assume that Θ is specified by a single linear constraint of the form:

$$D_\mu \vartheta^\mu + d \geq 0 \tag{A5}$$

Without loss of generality, we also take the constraint to be expressed in Hessian normal form, namely, $\|D_\mu\| = 1$. For clarity, note this assumption on the shape of Θ is used only from subsubsection A.2.3 onward.

A.2.2 Preliminaries

We now proceed to set up a low-temperature expansion of Equation A4 around the saddle point $\hat{\vartheta}$. We start by rewriting the numerator in Equation A4 as:

$$\int_\Theta \mathrm{d}^d \vartheta \exp[-N\left(-\frac{1}{2N}\ln\det g - \frac{1}{N}\ln \mathbb{P}(X|\vartheta)\right)] \tag{A6}$$

The idea of the FIA is to expand the integrand in Equation A6 in powers of N around the maximum likelihood point $\hat{\vartheta}$. To this end, we define three useful objects:

$$\tilde{I}_{\mu_1\cdots\mu_i} := -\frac{1}{N}\nabla_{\mu_1}\cdots\nabla_{\mu_i}\ln \mathbb{P}(X|\vartheta)\Big|_{\hat{\vartheta}} = -\frac{1}{N}\sum_{j=1}^N \nabla_{\mu_1}\cdots\nabla_{\mu_i}\ln \mathbb{P}(x_j|\vartheta)\Big|_{\hat{\vartheta}}$$

$$F_{\mu_1\cdots\mu_i} := \nabla_{\mu_1}\cdots\nabla_{\mu_i}\ln\det g(\vartheta)\Big|_{\hat{\vartheta}}$$

$$\psi := -\frac{1}{2N}\ln\det g - \frac{1}{N}\ln \mathbb{P}(X|\vartheta)$$

bioRxiv preprint doi: https://doi.org/10.1101/2023.01.10.523479; this version posted March 16, 2025. The copyright holder for this preprint (which was not certified by peer review) is the author/funder, who has granted bioRxiv a license to display the preprint in perpetuity. It is made available under aCC-BY 4.0 International license.

We immediately note that:

$$\nabla_{\mu_1}\cdots\nabla_{\mu_i}\psi\Big|_{\hat\vartheta} = \tilde I_{\mu_1\cdots\mu_i} - \frac{1}{2N}F_{\mu_1\cdots\mu_i}$$

which is useful to compute

$$\begin{aligned}\psi(\vartheta) &= \psi(\hat\vartheta) + \nabla_\mu\psi\Big|_{\hat\vartheta}(\vartheta^\mu-\hat\vartheta^\mu) + \frac{1}{2}\nabla_\mu\nabla_\nu\psi\Big|_{\hat\vartheta}(\vartheta^\mu-\hat\vartheta^\mu)(\vartheta^\nu-\hat\vartheta^\nu)+\ldots\\ &= \sum_{i=0}^{\infty}\frac{1}{i!}\nabla_{\mu_1}\cdots\nabla_{\mu_i}\psi\Big|_{\hat\vartheta}(\vartheta^{\mu_1}-\hat\vartheta^{\mu_1})\cdots(\vartheta^{\mu_i}-\hat\vartheta^{\mu_i})\\ &= \sum_{i=0}^{\infty}\frac{1}{i!}\nabla_{\mu_1}\cdots\nabla_{\mu_i}\psi\Big|_{\hat\vartheta}\prod_{k=1}^{i}(\vartheta^{\mu_k}-\hat\vartheta^{\mu_k})\end{aligned}$$

It is also useful to center the integration variables by introducing

$$\phi := \sqrt{N}(\vartheta-\hat\vartheta) \tag{A7}$$

$$\mathrm{d}^d\phi = N^{d/2}\,\mathrm{d}^d\vartheta \tag{A8}$$

so that

$$\nabla_{\mu_1}\cdots\nabla_{\mu_i}\psi\Big|_{\hat\vartheta}\prod_{k=1}^{i}(\vartheta^{\mu_k}-\hat\vartheta^{\mu_k}) = N^{-i/2}\left(\tilde I_{\mu_1\cdots\mu_i}-\frac{1}{2N}F_{\mu_1\cdots\mu_i}\right)\phi^{\mu_1}\cdots\phi^{\mu_i} \tag{A9}$$

and Equation A6 becomes:

$$\begin{aligned}\int \mathrm{d}^d\vartheta\exp[-N\psi] &= N^{-d/2}\int\mathrm{d}^d\phi\exp\left[-N\sum_{i=0}^{\infty}\frac{1}{i!}N^{-i/2}\left(\tilde I_{\mu_1\cdots\mu_i}-\frac{1}{2N}F_{\mu_1\cdots\mu_i}\right)\phi^{\mu_1}\cdots\phi^{\mu_i}\right]\\ &= N^{-d/2}\int\mathrm{d}^d\phi\exp\Bigg\{-N\left(-\frac{1}{N}\ln\mathbb{P}(X|\hat\vartheta)-\frac{1}{2N}\ln\det g(\hat\vartheta)\right)+\\ &\quad -N\left[\sum_{i=1}^{\infty}\frac{1}{i!}N^{-i/2}\left(\tilde I_{\mu_1\cdots\mu_i}-\frac{1}{2N}F_{\mu_1\cdots\mu_i}\right)\phi^{\mu_1}\cdots\phi^{\mu_i}\right]\Bigg\}\\ &= N^{-\frac{d}{2}}\exp[-\left(-\ln\mathbb{P}(X|\hat\vartheta)-\frac{1}{2}\ln\det g(\hat\vartheta)\right)]\times\\ &\quad\times\int\mathrm{d}^d\phi\exp\Bigg\{-N\Bigg[\frac{1}{\sqrt{N}}\tilde I_\mu\phi^\mu+\frac{1}{2N}\tilde I_{\mu\nu}\phi^\mu\phi^\nu+\\ &\quad+\frac{1}{N}\sum_{i=1}^{\infty}N^{-\frac{i}{2}}\left(\frac{1}{(i+2)!}\tilde I_{\mu_1\cdots\mu_{i+2}}\phi^{\mu_1}\cdots\phi^{\mu_{i+2}}-\frac{1}{2i!}F_{\mu_1\cdots\mu_i}\phi^{\mu_1}\cdots\phi^{\mu_i}\right)\Bigg]\Bigg\}\end{aligned}$$

Therefore,

$$\begin{aligned}\mathbb{P}(\mathcal{M}_1|X) &= N^{-\frac{d}{2}}\exp[-\left(-\ln\mathbb{P}(X|\hat\vartheta)-\frac{1}{2}\ln\det g(\hat\vartheta)+\ln\int\mathrm{d}^d\vartheta\sqrt{\det g}\right)]\times\\ &\quad\times\int\mathrm{d}^d\phi\exp\Bigg[-\sqrt{N}\tilde I_\mu\phi^\mu-\frac{1}{2}\tilde I_{\mu\nu}\phi^\mu\phi^\nu+\\ &\quad-\sum_{i=1}^{\infty}N^{-\frac{i}{2}}\left(\frac{1}{(i+2)!}\tilde I_{\mu_1\cdots\mu_{i+2}}\phi^{\mu_1}\cdots\phi^{\mu_{i+2}}-\frac{1}{2i!}F_{\mu_1\cdots\mu_i}\phi^{\mu_1}\cdots\phi^{\mu_i}\right)\Bigg]\Bigg\}\\ &= N^{-\frac{d}{2}}\exp[-\left(-\ln\mathbb{P}(X|\hat\vartheta)-\frac{1}{2}\ln\det g(\hat\vartheta)+\ln\int_\Theta\mathrm{d}^d\vartheta\sqrt{\det g}\right)]\cdot Q\end{aligned} \tag{A10}$$

bioRxiv preprint doi: https://doi.org/10.1101/2023.01.10.523479; this version posted March 16, 2025. The copyright holder for this preprint (which was not certified by peer review) is the author/funder, who has granted bioRxiv a license to display the preprint in perpetuity. It is made available under aCC-BY 4.0 International license.

where

$$Q = \int_\Phi \mathrm{d}^d\,\phi \exp\left[-\sqrt{N}\tilde{I}_\mu\phi^\mu - \frac{1}{2}\tilde{I}_{\mu\nu}\phi^\mu\phi^\nu - G(\phi)\right] \tag{A11}$$

and

$$G(\phi) = \sum_{i=1}^{\infty} N^{-\frac{i}{2}}\left(\frac{1}{(i+2)!}\tilde{I}_{\mu_1\cdots\mu_{i+2}}\phi^{\mu_1}\cdots\phi^{\mu_{i+2}} - \frac{1}{2i!}F_{\mu_1\cdots\mu_i}\phi^{\mu_1}\cdots\phi^{\mu_i}\right) \tag{A12}$$

where $G(\phi)$ collects the terms that are suppressed by powers of N.

Our problem has been now reduced to computing Q by performing the integral in Equation A11. Now our assumptions come into play for the key approximation step. For the sake of simplicity, assuming that N is large we drop $G(\phi)$ from the expression above, so that Q becomes a simple Gaussian integral with a linear term:

$$Q = \int_\Phi \mathrm{d}^d\,\phi \exp\left[-\sqrt{N}\tilde{I}_\mu\phi^\mu - \frac{1}{2}\phi^\mu\tilde{I}_{\mu\nu}\phi^\nu\right] \tag{A13}$$

A.2.3 Choosing a good system of coordinates

Consider now the Observed Fisher Information at the maximum likelihood, $\tilde{I}_{\mu\nu}$. As long as it is not singular, we can define its inverse $\Delta^{\mu\nu} = (\tilde{I}_{\mu\nu})^{-1}$. If $\tilde{I}_{\mu\nu}$ is positive definite, then the matrix representation of $\tilde{I}_{\mu\nu}$ has a set of d positive eigenvalues, which we denote by $\{\sigma_{(1)}^{-2}, \sigma_{(2)}^{-2}, \ldots, \sigma_{(d)}^{-2}\}$. The matrix representation of $\Delta^{\mu\nu}$ has eigenvalues $\{\sigma_{(1)}^{2}, \sigma_{(2)}^{2}, \ldots, \sigma_{(d)}^{2}\}$, and is diagonal in the same choice of coordinates as $\tilde{I}_{\mu\nu}$. We denote by U the (orthogonal) diagonalizing matrix; i.e., U is such that

$$U\Delta U^\intercal = \begin{bmatrix} \sigma_{(1)}^2 & 0 & \cdots & 0 \\ 0 & \sigma_{(2)}^2 & & \vdots \\ \vdots & & \ddots & 0 \\ 0 & \ldots & 0 & \sigma_{(d)}^2 \end{bmatrix}, \quad U^\intercal U = UU^\intercal = \mathbb{I} \tag{A14}$$

We define also the matrix K as the product of the diagonal matrix with elements $1/\sigma_{(k)}$ along the diagonal and U:

$$K = \begin{bmatrix} 1/\sigma_{(1)} & 0 & \cdots & 0 \\ 0 & 1/\sigma_{(2)} & & \vdots \\ \vdots & & \ddots & 0 \\ 0 & \ldots & 0 & 1/\sigma_{(d)} \end{bmatrix} U \tag{A15}$$

Note that

$$\det K = (\det \Delta^{\mu\nu})^{-1/2} = \sqrt{\det \tilde{I}_{\mu\nu}}$$

and that K corresponds to a sphering transformation, in the sense that

$$K\Delta K^\intercal = \mathbb{I} \quad \text{or} \quad K^\mu{}_\kappa \Delta^{\kappa\lambda} K^\nu{}_\lambda = \delta^{\mu\nu} \tag{A16}$$

and therefore, if we define the inverse

$$P = K^{-1}$$

we have

$$P^\intercal(\tilde{I}_{\mu\nu})P = \mathbb{I} \quad \text{or} \quad P^\kappa{}_\mu \tilde{I}_{\kappa\lambda} P^\lambda{}_\nu = \delta_{\mu\nu} \tag{A17}$$

We can now define a new set of coordinates by centering and sphering, as follows:

$$\xi^\mu = K^\mu{}_\nu\left(\phi^\nu + \sqrt{N}\Delta^{\nu\kappa}\tilde{I}_\kappa\right) \tag{A18}$$

Then,

$$\mathrm{d}^d\,\xi = \sqrt{\det \tilde{I}_{\mu\nu}}\,\mathrm{d}^d\,\phi \tag{A19}$$

bioRxiv preprint doi: https://doi.org/10.1101/2023.01.10.523479; this version posted March 16, 2025. The copyright holder for this preprint (which was not certified by peer review) is the author/funder, who has granted bioRxiv a license to display the preprint in perpetuity. It is made available under aCC-BY 4.0 International license.

and

$$\phi^\mu = P^\mu{}_\nu \xi^\nu - \sqrt{N}\Delta^{\mu\nu}\tilde{I}_\nu \tag{A20}$$

In this new set of coordinates,

$$\begin{aligned}
&-\sqrt{N}\tilde{I}_\nu\phi^\nu - \frac{1}{2}\phi^\mu \tilde{I}_{\mu\nu}\phi^\nu = \\
&= -\left(\sqrt{N}\tilde{I}_\nu + \frac{1}{2}\phi^\mu\tilde{I}_{\mu\nu}\right)\phi^\nu \\
&= -\left(\sqrt{N}\tilde{I}_\nu + \frac{1}{2}P^\mu{}_\kappa\xi^\kappa\tilde{I}_{\mu\nu}\frac{1}{2}\sqrt{N}\Delta^{\mu\kappa}\tilde{I}_\kappa\tilde{I}_{\mu\nu}\right)\phi^\nu \\
&= -\sqrt{N}\tilde{I}_\nu P^\nu{}_\lambda\xi^\lambda + N\Delta^{\nu\lambda}\tilde{I}_\lambda\tilde{I}_\nu - \frac{1}{2}P^\mu_\kappa\xi^\kappa\tilde{I}_{\mu\nu}P^\nu{}_\lambda\xi^\lambda + \frac{\sqrt{N}}{2}P^\mu{}_\kappa\xi^\kappa\tilde{I}_{\mu\nu}\Delta^{\nu\lambda}\tilde{I}_\lambda + \\
&\quad + \frac{\sqrt{N}}{2}\Delta^{\mu\kappa}\tilde{I}_\kappa\tilde{I}_{\mu\nu}P^\nu{}_\lambda\xi^\lambda - \frac{N}{2}\Delta^{\mu\kappa}\tilde{I}_\kappa\tilde{I}_{\mu\nu}\Delta^{\nu\lambda}\tilde{I}_\lambda \\
&= \frac{N}{2}\tilde{I}_\nu\Delta^{\nu\lambda}\tilde{I}_\lambda - \frac{1}{2}\xi^\kappa\delta_{\kappa\lambda}\xi^\lambda
\end{aligned} \tag{A21}$$

where we have used Equation A17 as well as the fact that $\Delta^{\mu\nu} = \Delta^{\nu\mu}$ and that $\Delta^{\mu\kappa}\tilde{I}_{\kappa\nu} = \delta^\mu{}_\nu$ by definition.

Therefore, putting Equation A19 and Equation A21 together, Equation A13 becomes

$$Q = \frac{\exp[\frac{N}{2}\tilde{I}_\mu\Delta^{\mu\nu}\tilde{I}_\nu]}{\sqrt{\det \tilde{I}_{\mu\nu}}}\int_\Xi \mathrm{d}^d\,\xi \exp[-\frac{1}{2}\xi_\mu\delta^{\mu\nu}\xi_\nu] \tag{A22}$$

The problem is reduced to a (truncated) spherical gaussian integral, where the domain of integration Ξ will depend on the original domain Θ but also on $\tilde{I}_\mu$, $\tilde{I}_{\mu\nu}$ and $\hat{\vartheta}$. To complete the calculation, we now need to make this dependence explicit.

A.2.4 Determining the domain of integration

We start by combining Equation A7 and Equation A20 to yield:

$$\vartheta^\mu = \frac{1}{\sqrt{N}}P^\mu{}_\nu\xi^\nu - \Delta^{\mu\nu}\tilde{I}_\nu + \hat{\vartheta}^\mu \tag{A23}$$

By substituting Equation A23 into Equation A5 we get

$$D_\mu\left(\frac{P^\mu{}_\nu\xi^\nu}{\sqrt{N}} - \Delta^{\mu\nu}\tilde{I}_\nu + \hat{\vartheta}^\mu\right) + d \geq 0$$

which we can rewrite as

$$\tilde{D}_\mu\xi^\mu + \tilde{d} \geq 0 \tag{A24}$$

with

$$\tilde{D}_\mu := \frac{1}{\sqrt{N}}D_\nu P^\nu{}_\mu \tag{A25}$$

and

$$\begin{aligned}
\tilde{d} &:= d + D_\mu\hat{\vartheta}^\mu - D_\mu\Delta^{\mu\nu}\tilde{I}_\nu \\
&= d + D_\mu\hat{\vartheta}^\mu - \langle D_\mu, \tilde{I}_\mu\rangle_\Delta
\end{aligned} \tag{A26}$$

where by $\langle\cdot,\cdot\rangle_\Delta$ we mean the inner product in the inverse observed Fisher information metric. Note that whenever $\tilde{I}_\mu$ is not zero, it will be parallel to D_μ. Indeed, by construction of the maximum-likelihood point $\hat{\vartheta}$, the gradient of the log likelihood can be orthogonal to the boundary only at $\hat{\vartheta}$, where it points towards the outside of the domain. Therefore $\tilde{I}_\mu$, which is defined as minus the gradient, will point inward. At the same time, D_μ will also always point

bioRxiv preprint doi: https://doi.org/10.1101/2023.01.10.523479; this version posted March 16, 2025. The copyright holder for this preprint (which was not certified by peer review) is the author/funder, who has granted bioRxiv a license to display the preprint in perpetuity. It is made available under aCC-BY 4.0 International license.

toward the interior of the domain because of the form of the constraint we have chosen in Equation A5. Because by assumption $\|D_\mu\| = 1$, we have that

$$\tilde{I}_\mu = \|\tilde{I}_\nu\| D_\mu$$

and

$$\langle D_\mu, \tilde{I}_\mu \rangle_\Delta = \|D_\nu\|_\Delta \cdot \|\tilde{I}_\nu\|_\Delta$$

so that

$$\tilde{d} = d + D_\mu \hat{\vartheta}^\mu - \|D_\mu\|_\Delta \cdot \|\tilde{I}_\mu\|_\Delta \tag{A27}$$

Now, the signed distance of the boundary to the origin in ξ-space is

$$l = -\frac{\tilde{d}}{\|\tilde{D}_\mu\|}$$

where the sign is taken such that l is negative when the origin is included in the integration domain. But noting that

$$K^\mu{}_\kappa \Delta^{\kappa\lambda} K^\nu{}_\lambda = \delta^{\mu\nu} \quad \Rightarrow \quad \Delta^{\mu\nu} = P^\mu{}_\kappa \delta^{\kappa\lambda} P^\nu{}_\lambda$$

we have

$$\begin{aligned} \|\tilde{D}_\mu\| &= \sqrt{\tilde{D}_\mu \delta^{\mu\nu} \tilde{D}_\nu} = \sqrt{\frac{1}{N} D_\kappa \left(P^\kappa{}_\mu \delta^{\mu\nu} P^\lambda{}_\nu\right) D_\lambda} \\ &= \sqrt{\frac{1}{N} D_\kappa \Delta^{\kappa\lambda} D_\lambda} = \frac{\|D_\mu\|_\Delta}{\sqrt{N}} \end{aligned}$$

and therefore

$$l = -\sqrt{N} \frac{\tilde{d}}{\|D_\mu\|} \tag{A28}$$

Finally, by plugging Equation A27 into Equation A28 we obtain

$$\begin{aligned} l &= -\sqrt{N} \left[\frac{d + D_\mu \hat{\vartheta}^\mu}{\|D_\mu\|_\Delta} - \|\tilde{I}_\mu\|_\Delta \right] \\ &=: \sqrt{2}\,(s - m) \end{aligned} \tag{A29}$$

where m and s are defined for convenience like so:

$$m := \sqrt{\frac{N}{2}} \frac{d + D_\mu \hat{\vartheta}^\mu}{\|D_\mu\|_\Delta} \quad (\geq 0) \tag{A30}$$

$$s := \sqrt{\frac{N}{2}} \|\tilde{I}_\mu\|_\Delta \quad (\geq 0) \tag{A31}$$

We note that m is a rescaled version of the margin defined by the constraint on the parameters (and therefore is never negative by assumption), and s is a rescaled version of the norm of the gradient of the log likelihood in the inverse observed Fisher metric (and therefore is nonnegative by construction).

A.2.5 Computing the penalty

We can now perform a final change of variables in the integral in Equation A22. We rotate our coordinates to align them to the boundary, so that

$$\tilde{D}_\mu = (\|\tilde{D}_\mu\|, 0, 0, \ldots, 0)$$

bioRxiv preprint doi: https://doi.org/10.1101/2023.01.10.523479; this version posted March 16, 2025. The copyright holder for this preprint (which was not certified by peer review) is the author/funder, who has granted bioRxiv a license to display the preprint in perpetuity. It is made available under aCC-BY 4.0 International license.

Note that we can always do this as our integrand is invariant under rotation. In this coordinate system, Equation A22 factorizes:

$$
\begin{aligned}
Q &= \frac{\exp[\frac{N}{2}\tilde{I}_\mu \Delta^{\mu\nu}\tilde{I}_\nu]}{\sqrt{\det \tilde{I}_{\mu\nu}}} \int_{\mathbb{R}^{d-1}} \mathrm{d}^{d-1}\,\xi \exp[-\frac{\xi_\mu \delta^{\mu\nu}\xi_\nu}{2}] \int_l^\infty \mathrm{d}\zeta \exp[-\frac{\zeta^2}{2}] \\
&= \sqrt{\frac{(2\pi)^d}{\det \tilde{I}_{\mu\nu}}} \exp[\frac{N}{2}\|\tilde{I}\|^2_\Delta] \frac{1}{\sqrt{\pi}} \int_l^\infty \frac{\mathrm{d}\zeta}{\sqrt{2}} \exp[-\frac{\zeta^2}{2}] \\
&= \sqrt{\frac{(2\pi)^d}{\det \tilde{I}_{\mu\nu}}} \exp(s^2) \frac{1}{\sqrt{\pi}} \int_{l/\sqrt{2}}^\infty \mathrm{d}\zeta \exp[-\zeta^2] \\
&= \sqrt{\frac{(2\pi)^d}{\det \tilde{I}_{\mu\nu}}} \exp(s^2) \frac{\operatorname{erfc}(s-m)}{2}
\end{aligned}
\tag{A32}
$$

where erfc(·) is the complementary error function (Abramowitz & Stegun, 1972, section 7.1.2).

Finally, plugging Equation A32 into Equation A10 and taking the log, we obtain the extended FIA:

$$
-\ln \mathbb{P}(\mathcal{M}_1|X) \simeq \ln \mathbb{P}(X|\hat{\vartheta}) + \frac{d}{2}\ln\frac{N}{2\pi} + \ln \int_\Theta \mathrm{d}^d\vartheta\,\sqrt{\det g} + \frac{1}{2}\ln\left[\frac{\det \tilde{I}_{\mu\nu}}{\det g_{\mu\nu}}\right] + B \tag{A33}
$$

where

$$
B := \ln(2) - \ln\left[\exp(s^2)\operatorname{erfc}(s-m)\right] \tag{A34}
$$

can be interpreted as a penalty arising from the presence of the boundary in parameter space.

A.2.6 Interpreting the penalty

We now take a closer look at Equation A34. One key observation is that, by construction, at most one of m and s is ever nonzero. In the interior of the manifold, $m > 0$ by definition, but $s = 0$ because the gradient of the likelihood is zero at $\hat{\vartheta}$. On the boundary, $m = 0$ by definition, and s can be either zero or positive.

Interior of the manifold. When $\hat{\vartheta}$ is in the interior of the parameter space Θ, then $\tilde{I}_\mu = 0 \Rightarrow s = 0$, and Equation A34 simplifies to

$$
B = \ln(2) - \ln(\operatorname{erfc}(-m)) \tag{A35}
$$

but because N is large we have $m \gg 0$, $\operatorname{erfc}(-m) \to 2$ and $B \to 0$, so our result passes the first sanity check: we recover the expression in Balasubramanian (1997).

Boundary of the manifold. When $\hat{\vartheta}$ is on the boundary of Θ, $m = 0$ and $s \geq 0$. Equation A34 becomes

$$
B = \ln(2) - \ln\left[\exp(s^2)\operatorname{erfc}(s)\right] = \ln(2) - \ln(w(is)) \tag{A36}
$$

where w is the Feddeeva function (Abramowitz & Stegun, 1972, p. 7.1.3):

$$
w(z) = e^{-z^2}\operatorname{erfc}(-iz)
$$

This function is tabulated and can be computed efficiently. However, it is interesting to analyze its limiting behavior, as follows.

As a consistency check, when s is small we have at fixed N, to first order:

$$
\begin{aligned}
B &\simeq \ln(2) - \ln(1 - \frac{2s}{\sqrt{\pi}}) \\
&\simeq \ln(2) + \frac{2s}{\sqrt{\pi}} = \ln(2) + \sqrt{\frac{2N}{\pi}}\|\tilde{I}_\mu\|_\Delta
\end{aligned}
\tag{A37}
$$

and $B = \ln(2)$ when $\tilde{I}_\mu = 0$, as expected.

bioRxiv preprint doi: https://doi.org/10.1101/2023.01.10.523479; this version posted March 16, 2025. The copyright holder for this preprint (which was not certified by peer review) is the author/funder, who has granted bioRxiv a license to display the preprint in perpetuity. It is made available under aCC-BY 4.0 International license.

However, the real case of interest is the behavior of the penalty when N is assumed to be large, which is consistent with the fact that we derived Equation A32 as an asymptotic expansion of Equation A11. In this case, using the asymptotic expansion for the Feddeeva function (Abramowitz & Stegun, 1972, section 7.1.23):

$$\exp[s^2]\,\mathrm{erfc}(s) \sim \frac{1}{s\sqrt{\pi}}\left[1+\sum_{m=1}^{\infty}(-1)^m\frac{1\cdot 3\cdots(2m-1)}{(2s^2)^m}\right]$$

To leading order, we obtain

$$\begin{aligned} B &\simeq \ln(2)+\ln(s\sqrt{\pi}) \\ &= \ln(2)+\ln(\sqrt{\frac{N\pi}{2}}\|\tilde{I}_\mu\|_\Delta) \end{aligned}$$

which we can rewrite as

$$\boxed{B \simeq \frac{1}{2}\ln\frac{N}{2\pi}+\ln\left[2\pi\|\tilde{I}_\mu\|_\Delta\right]} \tag{A38}$$

We can summarize the above by saying that a new penalty term of order $\ln N$ arose due to the presence of the boundary. Interestingly, comparing Equation A38 with Equation A33 we see that the first term in Equation A38 is analogous to counting an extra parameter dimension in the original Fisher Information Approximation.

A.3 Human psychophysics

The behavioral task required participants to view a screen showing two curves (one on the upper half, the other on the lower half of the screen) and 10 dots and decide, based on different instructions (see below for details), which curve was the more likely source of the observed dots. There were four task types that differed in terms of the shapes of the curves, corresponding to the different terms of the FIA (see Figure 1 in the main text and Figure B.1): *dimensionality*, *boundary*, *volume*, and *robustness*. In each case, the curves represent two parametric statistical models of the form:

$$p(x|t) = \frac{1}{\sqrt{2\pi\sigma^2}}\exp[-\frac{\|x-\mu(t)\|^2}{2\sigma^2}] \tag{A39}$$

where x is a location on the 2D plane visualized on the screen, and $\mu(t)$, $t \in [0,1]$ is a parametrization of the curve. In other words, the curves represent Gaussians of unit isotropic variance whose mean μ can be located at any point along them. The dots shown to the participants were sampled iid from one of the two models, selected at random with uniform probability. The location of the true mean of the Gaussian generating the dots (i.e., the value of t in the expression above) was sampled randomly from Jeffrey's prior for the selected model. All dots shown within a trial come from the same distribution (same model and same true mean). In the "generative" version of the task, the participants had to report which curve (model) the dots are more likely to come from. In the "maximum-likelihood" version, the participants had to report which curve was closest to the empirical centroid of the dot cloud. In both versions of the task, they pressed the "up" or "down" keys on their keyboard to select the curve in the upper or lower part of the screen, respectively.

Each model pairing was designed to emphasize a different term of the FIA. In the dimensionality variant, models have different dimensionality ($d = 0$ for the point and $d = 1$ for the line). In the boundary variant, both models have the same dimensionality and volume and are both flat so that their robustness terms are always identically zero. However, they are oriented such that, for ambiguous data falling around the midpoint between the two models, the influence of the boundary of the vertical model is stronger than that of the horizontal model. In the volume variant, models have the same dimensionality but different volumes (length). In the robustness variant, models have the same dimensionality and volume, but their curvature is such that one of them bends away from the region of data space that is more likely to contain ambiguous stimuli, whereas the other bends around it (and therefore the robustness term for these models has opposite sign for data points that fall in that region).

A single run of the task consisted of a brief tutorial followed by 500 trials, divided in 5 blocks of 100 trials each. For each trial, the chosen curve pairing was presented, randomly flipped vertically to dissociate a fixed preference for one of the two models from a fixed preference for reporting "up" or "down". At the end of each

bioRxiv preprint doi: https://doi.org/10.1101/2023.01.10.523479; this version posted March 16, 2025. The copyright holder for this preprint (which was not certified by peer review) is the author/funder, who has granted bioRxiv a license to display the preprint in perpetuity. It is made available under aCC-BY 4.0 International license.

block, the participant received feedback on their overall performance during that block. Participants received a fixed compensation for taking part in the experiment.

We report how we determined our sample size, all data exclusions (if any), all manipulations, and all measures in the study. We ran both experiments (generative and maximum-likelihood) on the online platform Pavlovia (pavlovia.org). For each task type, we collected data from at least 50 participants who passed a pre-established performance threshold (60% correct for the robustness task variant and 70% correct for the other variants; the sample size and the thresholds were chosen based on pilot data and were fixed at preregistration (Piasini et al., 2020, 2021b, 2022)). We discarded the data collected from all other participants. For the generative task, the final dataset included 52 participants for the robustness task variant and 50 participants for each of the other task variants. For the maximum-likelihood task, the final dataset included 51 participants for the dimensionality task variant and 50 participants for each of the others.

A.4 Detailed model definitions and computation of FIA terms

In this section, we report the detailed mathematical form of the models we used for the psychophysics experiment. Each model is defined by specifying the form of the function μ in Equation A39. Given this function, we then derive the analytical solution to the maximum-likelihood problem for any value of $X = \{x_i\}_{i=1}^N$, and finally the expressions for the likelihood (L), dimensionality (D), boundary (B), volume (V), and robustness (R) terms in the FIA for the model pairings we use in the experiment.

We also show that the (expected) Fisher information is constant for all models considered:

$$g(t) \equiv \frac{T^2}{\sigma^2} \tag{A40}$$

so that Jeffrey's prior is simply the uniform probability distribution over the $[0, 1]$ interval:

$$w(t) = \mathbb{1}_{[0,1]}(t) \tag{A41}$$

A.4.1 Fisher information and robustness term for curved exponential families

In the following, we compute the observed Fisher information for each of our models. To do so, it is convenient to have a general expression for the Hessian of the log likelihood and for the observed and expected Fisher information for curved exponential families.

The general form of a curved exponential family is:

$$p(x|u) = \exp\left[C(x) + \vartheta^i(u)F_i(x) - \psi(\vartheta(u))\right] \tag{A42}$$

where $\vartheta(u) : \mathbb{R}^d \to \mathbb{R}^k$, $k \geq d$, is a smooth parametrization. The Hessian of the log-likelihood is:

$$\begin{aligned}
\partial_a\partial_b \log p(x|u) &= F_i(x)\partial_a\partial_b\vartheta^i(u) - \partial_a\partial_b\psi(\vartheta(u)) \\
&= F_i(x)\frac{\partial^2\vartheta^i}{\partial u^a \partial u^b} - \frac{\partial}{\partial u^a}\left(\frac{\partial\psi}{\partial\vartheta^i}\frac{\partial\vartheta^i}{\partial u^b}\right) \\
&= F_i(x)\frac{\partial^2\vartheta^i}{\partial u^a \partial u^b} - \frac{\partial\vartheta^j}{\partial u^a}\frac{\partial\psi}{\partial\vartheta^j\partial\vartheta^i}\frac{\partial\vartheta^i}{\partial u^b} - \frac{\partial\psi}{\partial\vartheta^i}\frac{\partial^2\vartheta^i}{\partial u^a \partial u^b} \\
&= \frac{\partial^2\vartheta^i}{\partial u^a \partial u^b}\left[F_i(x) - \mathbb{E}_u[F_i]\right] - \frac{\partial\vartheta^j}{\partial u^a}g_{ji}\frac{\partial\vartheta^i}{\partial u^b}
\end{aligned} \tag{A43}$$

where we note that $g_{ij} = -\operatorname{Cov}_u[F]_{ji}$ (remember that by g_{ij} we indicate the Fisher information of the ambient family). Therefore, the (expected) Fisher information is:

$$g_{ab} = \mathbb{E}_u\left[-\partial_a\partial_b \log p(x_i|u)\right] = \frac{\partial\vartheta^j}{\partial u^a}g_{ji}\frac{\partial\vartheta^i}{\partial u^b} \tag{A44}$$

bioRxiv preprint doi: https://doi.org/10.1101/2023.01.10.523479; this version posted March 16, 2025. The copyright holder for this preprint (which was not certified by peer review) is the author/funder, who has granted bioRxiv a license to display the preprint in perpetuity. It is made available under aCC-BY 4.0 International license.

and the oberved Fisher information is:

$$\begin{aligned} h_{ab} &= -\frac{1}{N}\sum_{i=1}^{N} \partial_a \partial_b \log p(x_i|u) \\ &= g_{ab} + \frac{\partial^2 \vartheta^i}{\partial u^a \partial u^b}\left[\mathbb{E}_u[F_i] - \frac{1}{N}\sum_{n=1}^{N} F_i(x_n)\right] \end{aligned} \tag{A45}$$

As a corollary, we note that $h_{ab} = g_{ab}$ whenever $\vartheta(\cdot)$ is an affine transformation, that is when

$$\vartheta^i(u) = A^i_a u^a + B^i \tag{A46}$$

For some constant A^i_b and B^i. In this case (which corresponds to autoparallel submanifolds in the exponential connection, (Amari & Nagaoka, 2000, Theorem 1.1)), the robustness term in the FIA is identically zero:

$$\vartheta^i(u) = A^i_a u^a + B^i \Rightarrow R(X;u) \equiv 0 \tag{A47}$$

A.4.2 General properties of curved 2D Gaussian models

Our models of interest, defined through Equation A39, are a special case of curved exponential families. They are all submanifolds of the same, larger model — the 2-dimensional exponential family of 2D Gaussian distributions with known isotropic covariance and unknown center. We call this larger family the *ambient family* $\mathcal{S} \supset \mathcal{M}$, composed by all probability distributions whose density is of the form:

$$p(x|\mu) = \frac{1}{2\pi\sigma^2}\exp\left[-\frac{\|x-\mu\|^2}{2\sigma^2}\right] \tag{A48}$$

We can reduce Equation A39 to the notation of Equation A42 by noting that

$$\begin{aligned} \ln p(x|t) &= -\frac{\|x-\mu\|^2}{2\sigma^2} - \ln(2\pi\sigma^2) \\ &= -\frac{1}{2}\|x\|^2_{g_{ij}} + \mu^i(t) g_{ij} x^j - \frac{1}{2}\left[\|\mu(t)\|^2_{g_{ij}} + \ln((2\pi)^2 \det g_{ij})\right] \end{aligned} \tag{A49}$$

where we indicate by g_{ij} the Fisher information of the ambient family $\mathcal{S}$:

$$g_{ij} = \begin{bmatrix} 1/\sigma^2 & 0 \\ 0 & 1/\sigma^2 \end{bmatrix} \tag{A50}$$

By comparing Equation A49 with Equation A42, we see that

$$C(x) = -\frac{1}{2}\|x\|^2_{g_{ij}} \tag{A51}$$

$$F^i(x) = x^i \qquad (\text{and } F_i(x) = g_{ij}x^j = \frac{x^i}{\sigma^2}) \tag{A52}$$

$$\psi(t) = \frac{1}{2}\left[\|\mu(t)\|^2_{g_{ij}} + \ln((2\pi)^2 \det g_{ij})\right] \tag{A53}$$

and that $\mu(t)$ plays the role that $\vartheta(u)$ played in Equation A42.

We can now compute the expected and observed Fisher information for our models by specializing Equation A44 and Equation A45:

$$g(t) = \dot\mu^i(t) g_{ij} \dot\mu^j(t) \tag{A54}$$

$$h(t) = g(t) + \ddot\mu^i(t) g_{ij}\left[\mu^j(t) - \bar{x}\right] \tag{A55}$$

bioRxiv preprint doi: https://doi.org/10.1101/2023.01.10.523479; this version posted March 16, 2025. The copyright holder for this preprint (which was not certified by peer review) is the author/funder, who has granted bioRxiv a license to display the preprint in perpetuity. It is made available under aCC-BY 4.0 International license.

Where $\bar{x}$ is the empirical centroid of the dataset X,

$$\bar{x} = \bar{x}(X) := \frac{1}{N}\sum_{i=1}^{N} x_i \tag{A56}$$

and g and h have no indices, because they are scalar functions of t.

We note then that $g(t)$ is simply the squared Euclidean norm of the vector $\dot{\mu}(t)$ divided by σ^2. In other words, the geometry of $\mathcal{M}$ coincides, up to scaling by σ^2, with the Euclidean geometry of the plane curve $\mu(t)$. This very convenient fact is a consequence of the particularly simple noise model we have assumed (Gaussian with known isotropic covariance).

Model volume. The volume of a model described by $\mu(\cdot)$ is

$$\int_0^1 \mathrm{d}t\, \sqrt{g(t)} = \int_0^1 \mathrm{d}t\, \sqrt{\dot{\mu}^i(t) g_{ij} \dot{\mu}^j(t)} = \frac{1}{\sigma}\int_0^1 \mathrm{d}t \|\dot{\mu}(t)\| \tag{A57}$$

In other words, it is simply the length of the curve $\mu(\cdot)$ measured in units of σ.

Likelihood gradient and maximum-likelihood point. In the following, we will indicate the log-likelihood function for a model by

$$l = l(x;t) = \ln p(x|t) \tag{A58}$$

In order to find the maximum-likelihood point for our models, it is convenient to write a general expression for the score function (the derivative of the log likelihood with respect to the parameter). We start by noting that

$$\begin{aligned} \frac{\partial}{\partial t}\ln p(X|t) &= -\sum_i \frac{\partial}{\partial t}\frac{\|x_i - \mu(t)\|^2}{2\sigma^2} = \frac{N}{2\sigma^2}\left[\frac{2}{N}\sum_n x_n - \mu(t)\right]\cdot \dot{\mu}(t) \\ &= N\frac{\partial}{\partial t}\ln p(\bar{x}|t) = N\frac{\partial l(\bar{x};t)}{\partial t} \end{aligned}$$

Therefore, to find the maximum likelihood point $\hat{t}$ for a certain X we can simply solve the corresponding one-sample ($N = 1$) case for the centroid $\bar{x}$. We can also write the rescaled likelihood gradient (which appears in the FIA as I_μ) as

$$-\frac{1}{N}\frac{\partial l}{\partial t}(X;t) = -\frac{1}{N}\frac{\partial}{\partial t}\ln p(X|\mu(t)) = \dot{\mu}^i(t) g_{ij}\left[\mu^j(t) - \bar{x}^j\right] \tag{A59}$$

If we interpret $\dot{\mu}(t)$ as the tangent vector to μ in t, we see that away from model boundaries this equation expresses the familiar condition that the maximum-likelihood point (where $\partial l/\partial t = 0$) is the (Euclidean) orthogonal projection of $\bar{x}$ onto the model manifold. Again, this convenient property is a consequence of assuming isotropic Gaussian noise.

A.4.3 Horizontal model

This model, used in the Dimensionality, Boundary, and Volume tasks, is defined as

$$\mu(t) = \begin{bmatrix} T\left(t - \frac{1}{2}\right) \\ \tau \end{bmatrix} \tag{A60}$$

It is immediately evident that this model has volume (length) T/σ. The "base" model corresponds to $T = 1$, $\tau = 0$, and the model type called "horizontal" is defined with $T = 3$, $\tau = 1$.

Because

$$\dot{\mu}(t) = T\begin{bmatrix} 1 \\ 0 \end{bmatrix} \tag{A61}$$

and following Equation A54 and Equation A55, the observed and expected Fisher information coincide and are given by

$$g = h = \frac{T^2}{\sigma^2} \tag{A62}$$

bioRxiv preprint doi: https://doi.org/10.1101/2023.01.10.523479; this version posted March 16, 2025. The copyright holder for this preprint (which was not certified by peer review) is the author/funder, who has granted bioRxiv a license to display the preprint in perpetuity. It is made available under aCC-BY 4.0 International license.

Given a centroid $X = [X^1, X^2]^\intercal$, the rescaled likelihood gradient is (from Equation A59)

$$\frac{1}{N}\frac{\partial l}{\partial t}(X;t) = \frac{T^2}{\sigma^2}\left[\frac{\bar{x}^1}{T} - \left(t - \frac{1}{2}\right)\right] \tag{A63}$$

and the maximum-likelihood point $\hat{t}$ is

$$\hat{t}(X) = \begin{cases} 0 & \text{if } \bar{x}^1 < -T/2 \\ \frac{1}{2} + \frac{\bar{x}^1}{T} & \text{if } -T/2 < \bar{x}^1 < T/2 \\ 1 & \text{if } \bar{x}^1 > T/2 \end{cases} \tag{A64}$$

All the FIA terms can be computed in closed form from these expressions:

$$L(X) = -\frac{N}{2\sigma^2}\left[(\bar{x}^1 - T(\hat{t}(X) - 1/2))^2 + (\bar{x}^2 - \tau)^2\right] - \frac{N}{2}\ln(2\pi\sigma^2) \tag{A65}$$

$$D = \frac{1}{2}\ln\frac{N}{2\pi} \tag{A66}$$

$$B = \frac{1}{2}\ln\frac{N}{2\pi} + \ln\left[2\pi\frac{T}{\sigma}\left|\frac{\bar{x}^1}{T} - \left(\hat{t}(X) - \frac{1}{2}\right)\right|\right] \quad \left(\text{if } |\bar{x}^1| > \frac{T}{2}\right) \tag{A67}$$

$$V = \ln\frac{T}{\sigma} \tag{A68}$$

$$R = 0 \tag{A69}$$

A.4.4 Vertical model

This model, used the Boundary task, is just a rotated and translated version of the horizontal model. It is defined as

$$\mu(t) = \begin{bmatrix} 0 \\ \tau + Tt \end{bmatrix} \tag{A70}$$

where we keep T and τ as arbitrary parameters for notational clarity, although in practice they are both fixed to 1 in our study. From the definition, it follows that

$$\dot{\mu}(t) = T\begin{bmatrix} 0 \\ 1 \end{bmatrix} \tag{A71}$$

$$g = h = \frac{T^2}{\sigma^2} \tag{A72}$$

$$\frac{1}{N}\frac{\partial l}{\partial t}(X;t) = \frac{T^2}{\sigma^2}\left[\frac{X^2 - \tau}{T} - t\right] \tag{A73}$$

and

$$\hat{t}(X) = \begin{cases} 0 & \text{if } \bar{x}^2 < \tau \\ \frac{\bar{x}^2 - \tau}{T} & \text{if } \tau < \bar{x}^2 < \tau + T \\ 1 & \text{if } \bar{x}^2 > \tau + T \end{cases} \tag{A74}$$

so that the FIA terms can be written as

$$L(X) = -\frac{N}{2\sigma^2}\left[(\bar{x}^1)^2 + (\bar{x}^2 - \tau - T\hat{t}(X))^2\right] - \frac{N}{2}\ln(2\pi\sigma^2) \tag{A75}$$

$$D = \frac{1}{2}\ln\frac{N}{2\pi} \tag{A76}$$

$$B = \frac{1}{2}\ln\frac{N}{2\pi} + \ln\left[2\pi\frac{T}{\sigma}\left|\frac{\bar{x}^2 - \tau}{T} - \hat{t}(X)\right|\right] \quad \left(\text{if } \bar{x}^2 < \tau \vee \bar{x}^2 > \tau + T\right) \tag{A77}$$

$$V = \ln\frac{T}{\sigma} \tag{A78}$$

$$R = 0 \tag{A79}$$

bioRxiv preprint doi: https://doi.org/10.1101/2023.01.10.523479; this version posted March 16, 2025. The copyright holder for this preprint (which was not certified by peer review) is the author/funder, who has granted bioRxiv a license to display the preprint in perpetuity. It is made available under aCC-BY 4.0 International license.

A.4.5 Circular-arc model

This model, used in the Robustness task, is constituted by an arc of a circle, and is defined as

$$\mu(t) = \begin{bmatrix} \frac{T}{\gamma}\sin(\alpha) \\ \tau + \frac{T}{\gamma}(1-\cos(\alpha)) \end{bmatrix} \tag{A80}$$

where

$$\alpha = \gamma\left(t - \frac{1}{2}\right) \tag{A81}$$

and γ is a positive constant. Concretely, in the experiments we fixed $\gamma = (3/5)\pi$ and T to the value determined below for the rounded model type (Equation A99). The radius of the circle is $r = T/\gamma$, and the y-coordinate of the center is $\tau + r$. The tangent vector $\dot{\mu}$ and the acceleration vector $\ddot{\mu}$ in t are, respectively:

$$\dot{\mu}(t) = T\begin{bmatrix} \cos(\alpha) \\ \sin(\alpha) \end{bmatrix} \tag{A82}$$

$$\ddot{\mu}(t) = T\gamma\begin{bmatrix} -\sin(\alpha) \\ \cos(\alpha) \end{bmatrix} \tag{A83}$$

so that, by substitution in Equation A54,

$$g = \dot{\mu}^i g_{ij}\dot{\mu}^j = \frac{T^2}{\sigma^2}(\cos^2(\alpha) + \sin^2(\alpha)) = \frac{T^2}{\sigma^2} \tag{A84}$$

and by substitution in Equation A55

$$\begin{aligned} h &= g + \frac{T^2}{\sigma^2}\left[-\sin^2(\alpha) + \frac{\gamma\bar{x}^1}{T}\sin(\alpha) + \frac{\gamma\tau}{T}\cos(\alpha) + \cos(\alpha) - \cos^2(\alpha) - \frac{\gamma\bar{x}^2}{T}\cos(\alpha)\right] \\ &= g\left[\frac{\gamma\bar{x}^1}{T}\sin(\alpha) + \frac{\gamma\tau}{T}\cos(\alpha) + \cos(\alpha) - \frac{\gamma\bar{x}^2}{T}\cos(\alpha)\right] \\ &= \frac{g}{r}\left[\sin(\alpha)\bar{x}^1 + \cos(\alpha)\left(\tau + r - \bar{x}^2\right)\right] \end{aligned} \tag{A85}$$

The rescaled likelihood gradient is (from Equation A59)

$$\begin{aligned} -\frac{1}{N}\frac{\partial}{\partial t}\ln(p(X|t)) &= \frac{T^2}{\gamma\sigma^2}\Big[\cos(\alpha)\sin(\alpha) - \frac{\gamma\bar{x}^1}{T}\cos(\alpha) \\ &\quad + \frac{\gamma\tau}{T}\sin(\alpha) + \sin(\alpha) - \sin(\alpha)\cos(\alpha) - \frac{\gamma\bar{x}^2}{T}\sin(\alpha)\Big] \\ &= \frac{T}{\sigma^2}\left[-\bar{x}^1\cos(\alpha) + \tau\sin(\alpha) + r\sin(\alpha) - \bar{x}^2\sin(\alpha)\right] \\ &= \frac{T}{\sigma^2}\left[-\cos(\alpha)\bar{x}^1 + \sin(\alpha)\left(\tau + r - \bar{x}^2\right)\right] \end{aligned} \tag{A86}$$

(note that h can also be obtained by differentiating this last expression).

To compute the FIA, we need the maximum-likelihood projection. As for the other models, this projection is defined piecewise because of the presence of model boundaries. To properly partition the plane, we need to define first the equation for the line intersecting the model perpendicularly at t:

$$\rho(x;t) = \tau + r - \cot(\alpha(t))x \tag{A87}$$

With this definition, the maximum-likelihood point is

$$\hat{t}(X) = \begin{cases} 0 & \text{if } \bar{x}^1 < 0 \wedge \bar{x}^2 > \rho(\bar{x}^1;0) \\ 1 & \text{if } \bar{x}^1 > 0 \wedge \bar{x}^2 > \rho(\bar{x}^1;1) \\ \frac{1}{2} + \frac{1}{\gamma}\arctan\frac{\bar{x}^1}{\tau + r - \bar{x}^2} & \text{otherwise} \end{cases} \tag{A88}$$

bioRxiv preprint doi: https://doi.org/10.1101/2023.01.10.523479; this version posted March 16, 2025. The copyright holder for this preprint (which was not certified by peer review) is the author/funder, who has granted bioRxiv a license to display the preprint in perpetuity. It is made available under aCC-BY 4.0 International license.

and therefore the FIA terms are:

$$L(X) = -\frac{N}{2\sigma^2}\|\bar{x} - \mu(\hat{t}(X))\|^2 - \frac{N}{2}\ln(2\pi\sigma^2) \tag{A89}$$

$$D = \frac{1}{2}\ln\frac{N}{2\pi} \tag{A90}$$

$$B = \frac{1}{2}\ln\frac{N}{2\pi} + \ln\left[2\pi\sqrt{\frac{r}{\sigma^2}\frac{\left[-\cos(\alpha(\hat{t}))\bar{x}^1 + \sin(\alpha(\hat{t}))\left(\tau + r - \bar{x}^2\right)\right]^2}{\sin(\alpha(\hat{t}))\bar{x}^1 + \cos(\alpha(\hat{t}))\left(\tau + r - \bar{x}^2\right)}}\right] \tag{A91}$$

$$V = \ln\frac{T}{\sigma} \tag{A92}$$

$$R = \sin(\alpha(\hat{t}))\frac{\bar{x}^1}{r} + \cos(\alpha(\hat{t}))\frac{\tau + r - \bar{x}^2}{r} \tag{A93}$$

where the value given for B is relevant only when $\hat{t}$ is either 0 or 1. Note that, because of the shape of the model and the presence of the boundary, there are regions of the data space such that the log-likelihood function at the maximum likelihood point will not be concave. These regions represent a complete breakdown of the FIA, but they are not a problem in practice because the approximation holds in the region of data space that is relevant for the experiments (see Figure B.1).

A.4.6 Rounded model

This model, also used in the Robustness task, is a circular arc (like the "circular" model described above) with two straight arms attached on either side. The ratio of the length of the circular section of the model over its total length is defined as a parameter f. The model definition is

$$\mu(t) = \begin{cases} \begin{bmatrix} -T\left[\frac{f}{\gamma}\sin(\gamma/2) - \left(t - \frac{(1-f)}{2}\right)\cos(\gamma/2)\right] \\ \tau + T\left[\frac{f}{\gamma}\left(1 - \cos(\gamma/2)\right) - \left(t - \frac{1-f}{2}\right)\sin(\gamma/2)\right] \end{bmatrix} & \text{if } t < \frac{1-f}{2} \\ \mu_c\left(\frac{t-(1-f)/2}{f}; \tau, \gamma, fT\right) & \text{if } \frac{1-f}{2} \leq t \leq \frac{1+f}{2} \\ \begin{bmatrix} T\left[\frac{f}{\gamma}\sin(\gamma/2) + \left(t - \frac{(1+f)}{2}\right)\cos(\gamma/2)\right] \\ \tau + T\left[\frac{f}{\gamma}\left(1 - \cos(\gamma/2)\right) + \left(t - \frac{1+f}{2}\right)\sin(\gamma/2)\right] \end{bmatrix} & \text{if } t > \frac{1+f}{2} \end{cases} \tag{A94}$$

where μ_c is the μ mapping defined for the circular model, Equation A80.

For the experiment, the values of the parameters were chosen to guarantee that the circular section of this model would have the same center as a circular model (described above) with $\gamma = (3/5)\pi$ and $\tau = 0$, and that a relatively large fraction of the two models is in close proximity. The values are

$$f = 1/3 \tag{A95}$$

$$\gamma = (3/5)\pi \tag{A96}$$

$$\tau = 3/5 \tag{A97}$$

$$T = \frac{\tau\gamma}{1-f} \tag{A98}$$

Closed-form expressions for all FIA terms can be derived for this model by a straightforward, if somewhat laborious, extension of those presented above for the circular-arc model. We do not report them here in the interest of brevity.

bioRxiv preprint doi: https://doi.org/10.1101/2023.01.10.523479; this version posted March 16, 2025. The copyright holder for this preprint (which was not certified by peer review) is the author/funder, who has granted bioRxiv a license to display the preprint in perpetuity. It is made available under aCC-BY 4.0 International license.

A.4.7 Point model

This model, used in the Dimensionality task variant, has no associated latent parameters (it is zero-dimensional). To cast it in the same language as the others, we can define it as

$$\mu(t) = \mu = \begin{bmatrix} 0 \\ \tau \end{bmatrix} \tag{A99}$$

For the point model, the FIA (which is an approximation to a model's log evidence) is replaced by the exact evidence, which simply coincides with the log likelihood. For notational consistency, we adopt the following values for the FIA terms:

$$L(X) = \frac{N}{2\sigma^2}\left[(\bar{x}^1)^2 + (\bar{x}^2 - \tau)^2\right] - \frac{N}{2}\ln(2\pi\sigma^2) \tag{A100}$$

$$D = 0 \tag{A101}$$

$$B = 0 \tag{A102}$$

$$V = 0 \tag{A103}$$

$$R = 0 \tag{A104}$$

A.5 Numerical experiments with Artificial neural networks

A.5.1 Inputs

On each trial, our artificial neural network ("ANN") takes as input: 1) two images, each depicting one candidate model's location in the data space; and 2) a length-20 vector, containing the horizontal and vertical coordinates of the $N = 10$ data points. Each of the two images is provided as one RGB matrix of size (3*256*256). In data-space units (used for the model definitions in subsection A.4), each image extends from $x = -4$ to $x = 4$ and from $y = -3.5$ to $y = 4.5$, so that the center of the image (located in $(0, 0.5)$) is equidistant from the models in each model pair.

A.5.2 Training dataset

The training dataset consisted of 5000 model pairs. Each model pair was used for generating 50 trials. This approach led to a total of 250,000 trials in the entire dataset.

The random generation of model pairs was performed as follows (see subsection A.4 for the detailed mathematical definitions of each model and the precise meaning of the parameters controlling its shape). Each model pair could be of one of the four variants described in subsection A.3, chosen randomly with equal probabilities. Each model pair could be flipped vertically with probability 0.5. For the robustness variant, the separation of the model pair was 0.6 data-space units; for all other model pairs, the separation was 1 data-space unit. For the dimensionality variant, the length T (in data-space units) of the one-dimensional model was sampled uniformly from $\mathcal{U}(0.5, 5)$. For the boundary variant, the length of both model families were kept identical and sampled from $\mathcal{U}(0.5, 3)$. For the volume variant, the lengths of both models were sampled independently from $\mathcal{U}(0.5, 5)$; if their length difference was no greater than the task's noise level $\sigma = 1$, then the length of one model was resampled from $\mathcal{U}(0.5, 5)$ until the length difference was greater than 1. For the robustness variant, the length of both model families was kept constant at $(27/50) \cdot \pi$. The length proportion of the rounded model that was perfectly circular was $f = 1/3$, and both model families share the same curvature parameter γ sampled from $\mathcal{U}(1.5, 3)$. The model pairs were centered around the center of each input image.

Given a model pair, each trial was generated randomly as follows: 1) select one model randomly with equal probability; 2) sample a location along this model uniformly; and 3) using this location as the center of a 2D isotropic Gaussian and standard deviation of $\sigma = 1$ data-space units, sample $N = 10$ data points that were given to the network.

The training dataset was pre-shuffled randomly for training purposes. The input batch size was always 50 trials.

bioRxiv preprint doi: https://doi.org/10.1101/2023.01.10.523479; this version posted March 16, 2025. The copyright holder for this preprint (which was not certified by peer review) is the author/funder, who has granted bioRxiv a license to display the preprint in perpetuity. It is made available under aCC-BY 4.0 International license.

A.5.3 Test dataset

The test dataset consisted of 8 model pairs, each generating 15,000 trials. Thus, there was a total of 120,000 trials in the dataset.

The model pairs were as follows. For the Dimensionality task, the one-dimensional model had length (in data space units) 1. For the Boundary task, both model families had length 1. For the Volume task, one model had length 1, the other had 3. For the Robustness task, both model families had length $T = (27/50) \cdot \pi$, $f = 1/3$, and curvature parameter $\gamma = (3/5)\pi$. Each model pair was presented in the "upright" position (as per the definitions in subsection A.4) and in the vertically flipped position, for a total of 8 cases. The separation between model families and the generation of trials was identical to as in the training dataset.

A.5.4 Artificial neural network architecture

Our ANN had the following architecture (see Figure 4). Each of the two model input images was passed through the pretrained convolutional neural network VGG16, which had its parameters frozen during training. We replaced the fully connected layers at the end of VGG16 with our own structure of Linear-ReLU-BatchNorm1D layers and allowed the updating of weights in these and all subsequent layers. For each image input, the output of this image-processing module was a length-50 vector (model image representation).

In parallel, the length-20 vector of raw data point coordinates was fed through a permutation-invariant layer. This layer featured shared weights such that its outputs were not affected by the sequence of the N=10 data points in the length-20 vector input. This layer also outputted a length-20 vector, which was concatenated to the end of each of the length-50 vectors (the model image representations) along the preexisting dimension, producing two length-70 vectors.

Each length-70 vector was fed through Linear-ReLU-BatchNorm1D layers (identical weights used to process each vector). The resultant two length-50 output vectors were then concatenated together along the preexisting dimension, with the first input image's representation in front.

The resultant length-100 vector was then fed through EquiLinear-ReLU-BatchNorm1D layers. The EquiLinear layers were permutation-equivariant layers of our design, again achieved by weight sharing. They ensure that if we concatenated the two length-50 output vectors in the opposite sequence, then their output, a length-2 vector, also had the same values but in opposite sequence. This length-2 vector was passed through a log softmax layer to produce the ANN's final output, which was also a length-2 vector.

We also introduced a conditional variational encoder (CVAE) structure and used its output as part of the loss function (discussed later), to encourage model representations to preserve information about the data generation process. The details are described below.

We concatenated the length-20 raw data points vector (before passing input the permutation-invariant layers) to the end of each length-50 model image representation vector. The resultant two length-70 vectors (each corresponding to one model) were used as inputs for our CVAE (identical weights used to process each vector). The CVAE took each length-70 vector through its encoder structure to produce 10-dimensional vectors, which were used as parameters $(\mu_{CVAE}, \sigma_{CVAE})$ for the Gaussian random generation of another 10-dimensional vector. The latter vector was again concatenated to the end of the length-50 model image representation vector responsible for its own generation, before being fed to the CVAE decoder, which mapped back to a 20-dimensional output vector reminiscent of data points. Hence, there were two 20-dimensional output vectors generated, each originating from one model.

A.5.5 Loss function

The loss function for each trial consisted of 2 parts: 1) the final output loss, and 2) the CVAE output loss. For the final output loss, we used Pytorch's negative log likelihood loss function `NLLLoss()`, which computed the loss between the ANN's length-2 output vector and the target label. For each trial's CVAE output loss, we considered only the CVAE output associated with the correct model image/target label (hence one out of the two CVAE output vectors). The CVAE output loss was the sum of a MSE reconstruction loss (between the length-20 CVAE output vector and the length-20 raw data points vector) and a KL Divergence Loss (considering $(\mu_{CVAE}, \sigma_{CVAE})$ used in the CVAE data generation process, using sum reduction). The total loss was the sum of the final output loss and the CVAE output loss.

bioRxiv preprint doi: https://doi.org/10.1101/2023.01.10.523479; this version posted March 16, 2025. The copyright holder for this preprint (which was not certified by peer review) is the author/funder, who has granted bioRxiv a license to display the preprint in perpetuity. It is made available under aCC-BY 4.0 International license.

A.5.6 Update rule

We used Pytorch's Adam optimizer with learning rate 0.005, keeping all other arguments at their default values.

A.5.7 ANN predictions

To evaluate ANN task performance in a way that was comparable to human performance, we needed to specify how the ANN output, a length-2 log softmax vector, mapped onto a chosen candidate model. The mapping was as follows: we compared the two entries in the output vector and assumed that the ANN chose the candidate model associated with the larger entry.

A.6 Experimental data analysis

For both human and ANN experiments, we modeled behavior assuming that each observer samples from a posterior over models determined by a modified version of the FIA, where each term of the approximation is multiplied by a free parameter to be inferred, representing the sensitivity of the participant to that term.

Specifically, in our experimental scenario the theory of Bayesian model selection applies directly. Given two models $\mathcal{M}_1$ and $\mathcal{M}_2$, assuming a flat prior over models $p(\mathcal{M}_1) = p(\mathcal{M}_2) = 1/2$ and an uninformative prior (Jeffrey's prior, see Balasubramanian (1997) and Jaynes (2003)) over the parameters of each model, when N is sufficiently large we can use the asymptotic expansion in Figure 1 and Equation A33 to write the log posterior ratio for $\mathcal{M}_1$ over $\mathcal{M}_2$ as

$$\begin{aligned}\log \frac{p(\mathcal{M}_1|X)}{p(\mathcal{M}_2|X)} =& \log \frac{p(\mathcal{M}_1|X)}{1-p(\mathcal{M}_1|X)} \\ \simeq& (L_2 - L_1) + (D_2 - D_1) + (B_2 - B_1) + (V_2 - V_1) + (R_2 - R_1)\end{aligned} \tag{A105}$$

where L_i, D_i, etc represent the FIA terms for model i:

$$\begin{aligned} L_i &= -\log p(X|\hat{\vartheta}, \mathcal{M}_i) \quad \text{(Likelihood)} \\ D_i &= \frac{d}{2} \log \frac{N}{2\pi} \quad \text{(Dimensionality)} \\ B_i &= \frac{1}{2} \log \frac{N}{2\pi} + \log \left[2\pi \left\| \hat{l} \right\|_{\hat{h}^{-1}} \right] \quad \text{(Boundary)} \\ V_i &= \log \int \mathrm{d}^d \vartheta \sqrt{\det g(\vartheta)} \quad \text{(Volume)} \\ R_i &= \frac{1}{2} \log \left[\frac{\det h(X; \hat{\vartheta})}{\det g(\hat{\vartheta})} \right] \quad \text{(Robustness)} \end{aligned}$$

This expression suggests a simple normative model for participant behavior. Equation A105 determines the probability of reporting $\mathcal{M}_1$ for an ideal Bayesian observer performing probability matching. We can then compare participant behavior to the normative prescription by allowing participants to have distinct sensitivities to the various terms of the FIA:

$$\begin{aligned}\log \frac{p(\text{report } \mathcal{M}_1|X)}{p(\text{report } \mathcal{M}_2|X)} =& \alpha + \beta_L(L_2 - L_1) + \beta_D(D_2 - D_1) + \\ &+ \beta_B(B_2 - B_1) + \beta_V(V_2 - V_1) + \beta_R(R_2 - R_1)\end{aligned} \tag{A106}$$

where α and β were free parameters: α captures any fixed bias, β_L the sensitivity to differences in maximum likelihood, β_D the sensitivity to differences in dimensionality, and so on.

We fitted the model expressed by Equation A106 to participant behavior using a hierarchical, Bayesian

bioRxiv preprint doi: https://doi.org/10.1101/2023.01.10.523479; this version posted March 16, 2025. The copyright holder for this preprint (which was not certified by peer review) is the author/funder, who has granted bioRxiv a license to display the preprint in perpetuity. It is made available under aCC-BY 4.0 International license.

logistic-regression scheme:

$$\nu_\alpha, \nu_L, \ldots, \nu_R \sim 1 + \text{Exponential}(29) \tag{A107}$$
$$\mu_\alpha, \mu_L, \ldots, \mu_R \sim \text{Normal}(0, 3) \tag{A108}$$
$$\sigma_\alpha, \sigma_L, \ldots, \sigma_R \sim \text{Exponential}(3) \tag{A109}$$
$$\alpha_i \sim \text{StudentT}(\nu_\alpha, \mu_\alpha, \sigma_\alpha) \tag{A110}$$
$$\beta_{L,i} \sim \text{StudentT}(\nu_L, \mu_L, \sigma_L) \tag{A111}$$
$$\vdots \tag{A112}$$
$$\beta_{R,i} \sim \text{StudentT}(\nu_R, \mu_R, \sigma_R) \tag{A113}$$
$$C_{i,t} \sim \text{Bernoulli}\left(\text{logit}^{-1}\left(\text{lpr}\left(\alpha_i, \beta_{L,i}, \beta_{D,i}, \beta_{B,i}, \beta_{V,i}, \beta_{R,i}, X_{i,t}\right)\right)\right) \tag{A114}$$

where $C_{i,t}$ is the choice made by participant i on trial t, $X_{i,t}$ is the sensory stimulus on that same trial, lpr is the log posterior ratio defined by Equation A106, α_i is the bias for participant i, $\beta_{L,i}$ is the likelihood sensitivity of that same participant, and so on for the other sensitivity parameters. The bias and sensitivity parameters describing each participant are modeled as independent samples from a population-level Student-T probability distribution characterized by a certain shape (ν), location (μ), and scale (σ). The priors assumed over these population-level parameters are standard weakly informative priors (Gelman et al., 2014; Kruschke, 2015). The model was implemented in PyMC (Salvatier et al., 2016), and inference was performed by sampling from the posterior for the parameters given the experimental data $\{C_{i,t}, X_{i,t}\}$ using the No-U-Turn Sampler algorithm (Betancourt, 2018; Hoffman & Gelman, 2014). Further technical details on the inference procedure can be found in subsubsection A.6.2.

Definition of relative sensitivity. Relative sensitivity for a certain feature was defined as the sensitivity for that feature divided by the relevant posterior mean for the likelihood sensitivity. For instance, for dimensionality:

$$\tilde{\beta}_D = \frac{\beta_D}{\langle \beta_L \rangle_{p(\beta_L|\text{data})}} \tag{A115}$$

This formulation applies both at the participant level and at the population level.

Because each human participant performed only one task variant, not all sensitivities could be estimated for all participants. For instance, β_D entered the behavioral model (and therefore could be estimated) only for the participants that performed the Dimensionality task, where the alternative models had different dimensionality. The same holds with β_V and the Volume task, and β_R and the Robustness task. The boundary term entered the behavioral model for all task variants, although by design it took on a much broader range of values for the Boundary task. For consistency, for each sensitivity parameter, we reported its estimate only for those participants that performed the task variant designed to test it.

A.6.1 Lapse rates

We designed a variant of the behavioral model that accounts for lapses in participants' responses (i.e., errors on easy trials). Specifically, we modified Equation A106 as follows:

$$\begin{aligned}\log\frac{p(\text{report } \mathcal{M}_1|X)}{p(\text{report } \mathcal{M}_2|X)} =& \frac{\epsilon}{2} + (1-\epsilon)\left[\alpha + \beta_L(L_2 - L_1) + \beta_D(D_2 - D_1) + \right. \\ & \left. + \beta_B(B_2 - B_1) + \beta_V(V_2 - V_1) + \beta_R(R_2 - R_1)\right]\end{aligned} \tag{A116}$$

where $\epsilon \in [0, 1]$ is the lapse rate, representing the probability that a given response is completely random. For $\epsilon = 1$ the responses are random on every trial, whereas for $\epsilon = 0$ this model is equivalent to the original one in Equation A106.

To estimate ϵ from our experimental data jointly with all other parameters, we kept the same structure as in Equations A107–A114 and extended it by modeling the population level distribution of ϵ as a Beta distribution, parameterized by count parameters a and b. Following the recommendations by Gelman et al. (2014, section 5.3) and development team (2022, section 24.2), we specify hyperpriors in terms of the mean of the distribution $\phi = a/(a + b)$

bioRxiv preprint doi: https://doi.org/10.1101/2023.01.10.523479; this version posted March 16, 2025. The copyright holder for this preprint (which was not certified by peer review) is the author/funder, who has granted bioRxiv a license to display the preprint in perpetuity. It is made available under aCC-BY 4.0 International license.

Parameter	ESS	$\hat{R}$
μ_α	52148	1.000
μ_L	19254	1.000
μ_D	27384	1.000
μ_B	32434	1.000
μ_V	64614	1.000
μ_R	112118	1.000

Table A.1

$\hat{R}$ statistic and effective sample size (ESS) for 12 Markov Chain traces run as described in the text, for the fit to human data for the generative task. See Gelman et al. (2014, sections 11.4–11.5) and Vehtari et al. (2020) for in-depth discussion of chain quality diagnostics. Briefly, $\hat{R}$ depends on the relationship between the variance of the draws estimated within and between contiguous draw sequences. $\hat{R}$ is close to 1 when the chains have successfully converged. The effective sample size estimates how many independent samples one would need to extract the same amount of information as that contained in the (correlated) MCMC draws.

and the total count $\lambda = a + b$:

$$\phi \sim \text{Uniform}(0, 1) \tag{A117}$$

$$\lambda \sim \text{Pareto}(0.1, 1.5) \quad \left(p(\lambda) \propto \lambda^{-2.5}\right) \tag{A118}$$

$$a = \lambda \cdot \phi \tag{A119}$$

$$b = \lambda \cdot (1 - \phi) \tag{A120}$$

$$\epsilon_i \sim \text{Beta}(a, b) \tag{A121}$$

where ϵ_i is the lapse rate for participant i.

A.6.2 Technical details of the inference procedure

Posterior sampling was performed with PyMC (Salvatier et al., 2016) version 4.2.0, using the NUTS Hamiltonian Monte Carlo algorithm (Hoffman & Gelman, 2014). Target acceptance probability was set to 0.9 for the human data (both generative and maximum-likelihood task), to 0.8 for the generative task for ANNs, and 0.99 for the maximum-likelihood task for ANNs. The posterior distributions were built by sampling 12 independent Markov chains for 10000 draws each. No divergence occurred in any of the chains. Effective sample size and $\hat{R}$ diagnostics for some of the key parameters are given in Table A.1.

A.6.3 Reporting of posterior distributions for inferred parameters

The posterior distributions reported in all figures are Kernel Density Estimates with bandwidth chosen according to Scott's rule (Pedregosa et al., 2011).

A.7 Fitting the NIN observer to participant data

We fit the NIN model to our human behavioral data by maximum likelihood, on a single-participant basis and marginalizing numerically over the effect of sensory noise. In particular, say that $\bar{x}$ is the centroid of the dot cloud. Recall that the NIN model involves a latent (internal) sensory noise process that corrupts the sensory data by adding noise $\eta \sim \mathcal{N}(0, \rho)$:

$$\tilde{x} = \bar{x} + \eta$$

Then, the model observer computes a Bayesian posterior $q(\mathcal{M}_1|\tilde{x})$ by integrating locally around the maximum-likelihood point $\hat{\theta}(\tilde{x})$. The range of the local integration is controlled by an integration parameter b, so we write

$$q_1 = q(\mathcal{M}_1; b|\tilde{x})$$

bioRxiv preprint doi: https://doi.org/10.1101/2023.01.10.523479; this version posted March 16, 2025. The copyright holder for this preprint (which was not certified by peer review) is the author/funder, who has granted bioRxiv a license to display the preprint in perpetuity. It is made available under aCC-BY 4.0 International license.

Finally, choice noise is implemented through a softmax transform:

$$p(\text{choose } \mathcal{M}_1|x) = \frac{e^{\beta q_1}}{e^{\beta q_1} + e^{\beta q_2}}$$

where $\beta = 1/T$ is the "inverse temperature" parameter controlling the intensity of the choice noise. Inference is based on calculating the (log)likelihood of the model (parameterized by ρ, b and β), obtained by marginalizing over the sensory noise η:

$$\begin{aligned}\mathcal{L}(\rho, b, \beta) = \sum_{i=1}^{N} \log p(c_i|\bar{x}_i; \rho, b, \beta) &= \sum_{i=1}^{N} \log \int \mathrm{d}\eta p(\eta) \frac{e^{\beta q(c_i;b|\bar{x}_i+\eta)}}{e^{\beta q(c_i;b|\bar{x}_i+\eta)} + e^{\beta(1-q(c_i;b|\bar{x}_i+\eta))}} \\ &= \sum_{i=1}^{N} \log \int \mathrm{d}\eta \frac{1}{2\pi\rho^2} \exp\left[-\frac{\|\eta\|^2}{2\rho^2}\right] \frac{e^{\beta q(c_i;r|\bar{x}_i+\eta)}}{e^{\beta q(c_i;r|\bar{x}_i+\eta)} + e^{\beta(1-q(c_i;r|\bar{x}_i+\eta))}}\end{aligned} \tag{A122}$$

We approximate the integral in Equation A122 using a randomized quasi-Monte Carlo (QMC) approach (Owen, 2023):

$$\mathcal{L}(\rho, b, \beta) \simeq \sum_{i=1}^{N} \log \sum_{k=1}^{K} \frac{e^{\beta q(c_i;r|\bar{x}_i+\eta_{ik})}}{e^{\beta q(c_i;r|\bar{x}_i+\eta_{ik})} + e^{\beta(1-q(c_i;r|\bar{x}_i+\eta_{ik}))}} \tag{A123}$$

where $\{\eta_{ik}\}_{k=1}^{K}$, for any i, is a bivariate normal QMC set of points generated from a scrambled Sobol' sequence with $K = 1024$ using the `scipy.stats.qmc` module (Roy et al., 2023), centered in $(0, 0)$ and with variance ρ^2. We used an independent set of QMC points for each participant and each trial, but we kept this set fixed throughout the optimization (that is, we did not resample the QMC set at each iteration of the optimization procedure) to avoid introducing stochasticity in the loss function and to help with convergence.

To fit the model, we maximised the likelihood Equation A123 using Differential Evolution (Storn & Price, 1997) as implemented in the `scipy.optimize` module with a candidate population of size 64. The model parameters were constrained to the following ranges: $\rho \in (0, 2]$, $b \in [0, 1]$, $\beta \in [0, 10]$.

References

Abramowitz, M., & Stegun, I. A. (1972). *Handbook of mathematical functions: With formulas, graphs, and mathematical tables.* Dover.

Amari, S.-i., & Nagaoka, H. (2000). *Methods of information geometry* (D. Harada, Trans.). American Mathematical Society.

Balasubramanian, V. (1997). Statistical Inference, Occam's Razor, and Statistical Mechanics on the Space of Probability Distributions. *Neural Computation*, *9*(2), 349–368. https://doi.org/10.1162/neco.1997.9.2.349

Betancourt, M. (2018, July 15). *A Conceptual Introduction to Hamiltonian Monte Carlo.* arXiv: 1701.02434 `[stat]`. https://doi.org/10.48550/arXiv.1701.02434

development team, S. (2022). *Stan Modeling Language Users Guide, version 2.31.*

Gelman, A., Carlin, J. B., Stern, H. S., Dunson, D. B., Vehtari, A., & Rubin, D. B. (2014). *Bayesian Data Analysis* (3rd ed.). CRC Press.

Hoffman, M. D., & Gelman, A. (2014). The No-U-Turn Sampler: Adaptively Setting Path Lengths in Hamiltonian Monte Carlo. *Journal of Machine Learning Research*, *15*(47), 1593–1623. http://jmlr.org/papers/v15/hoffman14a.html

Jaynes, E. T. (2003, April 1). *Probability Theory: The Logic of Science.* Cambridge University Press.

Kruschke, J. K. (2015). *Doing Bayesian Data Analysis* (2nd ed.). Academic Press.

Owen, A. (2023). *Practical Quasi-Monte Carlo Integration.* https://artowen.su.domains/mc/practicalqmc.pdf

Pedregosa, F., Varoquaux, G., Gramfort, A., Michel, V., Thirion, B., Grisel, O., Blondel, M., Prettenhofer, P., Weiss, R., Dubourg, V., Vanderplas, J., Passos, A., Cournapeau, D., Brucher, M., Perrot, M., & Duchesnay, É. (2011). Scikit-learn: Machine Learning in Python. *Journal of Machine Learning Research*, *12*(85), 2825–2830. Retrieved January 6, 2023, from http://jmlr.org/papers/v12/pedregosa11a.html

Piasini, E., Balasubramanian, V., & Gold, J. I. (2020). *Preregistration document.* https://doi.org/10.17605/OSF.IO/2X9H6

bioRxiv preprint doi: https://doi.org/10.1101/2023.01.10.523479; this version posted March 16, 2025. The copyright holder for this preprint (which was not certified by peer review) is the author/funder, who has granted bioRxiv a license to display the preprint in perpetuity. It is made available under aCC-BY 4.0 International license.

Piasini, E., Balasubramanian, V., & Gold, J. I. (2021a). Effect of Geometric Complexity on Intuitive Model Selection. *The First International Symposium on AI and Neuroscience - ACAIN 2021.*

Piasini, E., Balasubramanian, V., & Gold, J. I. (2021b). *Preregistration document addendum.* https://doi.org/10.17605/OSF.IO/5HDQZ

Piasini, E., Liu, S., Balasubramanian, V., & Gold, J. I. (2022). *Preregistration document addendum.* https://doi.org/10.17605/OSF.IO/826JV

Roy, P. T., Owen, A. B., Balandat, M., & Haberland, M. (2023). Quasi-Monte Carlo Methods in Python. *Journal of Open Source Software*, *8*(84), 5309. https://doi.org/10.21105/joss.05309

Salvatier, J., Wiecki, T. V., & Fonnesbeck, C. (2016). Probabilistic programming in Python using PyMC3. *PeerJ Computer Science*, *2*, e55. https://doi.org/10.7717/peerj-cs.55

Storn, R., & Price, K. (1997). Differential Evolution – A Simple and Efficient Heuristic for global Optimization over Continuous Spaces. *Journal of Global Optimization*, *11*(4), 341–359. https://doi.org/10.1023/A:1008202821328

Vehtari, A., Gelman, A., Simpson, D., Carpenter, B., & Bürkner, P.-C. (2020). Rank-Normalization, Folding, and Localization: An Improved $\hatR$ for Assessing Convergence of MCMC. *Bayesian Analysis.* https://doi.org/10.1214/20-ba1221

bioRxiv preprint doi: https://doi.org/10.1101/2023.01.10.523479; this version posted March 16, 2025. The copyright holder for this preprint (which was not certified by peer review) is the author/funder, who has granted bioRxiv a license to display the preprint in perpetuity. It is made available under aCC-BY 4.0 International license.

Appendix B
Supplementary information

B.1 Numerical comparison of the extended FIA vs exact Bayes

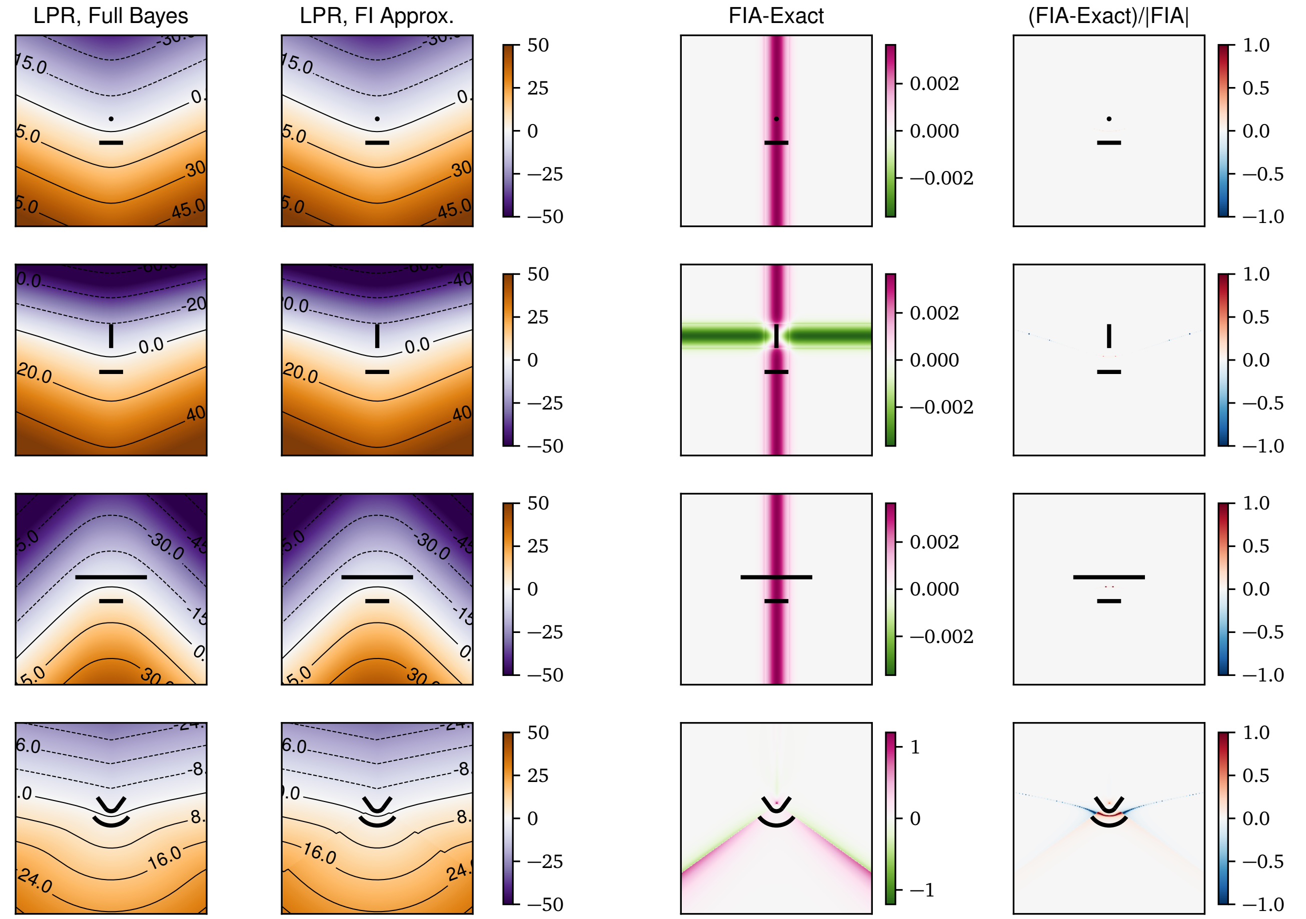

Figure B.1

Comparison of the full Bayesian and FIA computation of the log posterior ratio (LPR) for the model pairs used in our psychophysical tasks ($N = 10$). Each row corresponds to one task variant (from top to bottom, "dimensionality", "boundary", "volume", "robustness"). First column from the left: full Bayesian LPR, computed by numerical integration. Second column: LPR computed with the FIA. Third column: difference between FIA and exact LPR. Fourth column: relative difference (difference divided by the absolute value of the FIA LPR). Adapted from Piasini et al. (2021a).

Figure B.1 shows that the FIA computed with the expressions given in this document provides a very good approximation to the exact Bayesian log posterior ratio (LPR) for the model pairs used in the psychophysics experiments, and for the chosen sample size ($N = 10$). As highlighted in the panels in the rightmost column, the discrepancies between the exact and the approximated LPR are generally small in relative terms, and therefore are not very important for the purpose of model fitting and interpretation. Note that here, as well as for the results in the main text, the B term in the FIA is computed using Equation A34 rather than Equation A38 to avoid infinities (that for finite N can arise when the likelihood gradient is very small) and discontinuities (that for finite N can arise on the interior of the manifold, in proximity to the boundary, where the value of B goes from zero when $\hat{\vartheta}$ is in the interior to $\log(2)$ when $\hat{\vartheta}$ is exactly on the boundary).

Even though overall the agreement between the approximation is good, it is interesting to look more closely at where the approximation is poorest. The task type for which the discrepancies are the largest (both in absolute and relative terms) is the "robustness" type (fourth row in Figure B.1). This discrepancy arises because the FIA

bioRxiv preprint doi: https://doi.org/10.1101/2023.01.10.523479; this version posted March 16, 2025. The copyright holder for this preprint (which was not certified by peer review) is the author/funder, who has granted bioRxiv a license to display the preprint in perpetuity. It is made available under aCC-BY 4.0 International license.

hypotheses are not fully satisfied everywhere for one of the models. Specifically, the models in that task variant are a circular arc (the bottom model in Figure B.1, third row) and a smaller circular arc, concentric with the first, with a straight segment attached to either side (the top model). The log-likelihood function for this second model is smooth only to first order, but its second derivative (and therefore its Fisher Information and its observed Fisher Information) is not continuous at the points where the circular arc is joined with the straight segments, locally breaking hypothesis number 3 in subsubsection A.2.1. Geometrically, this discontinuity is analogous to saying that the curvature of the manifold changes abruptly at the joints. It is likely that the FIA for a model with a smoother transition between the circular arc and the straight arms would have been even closer to the exact value for all points on the 2D plane (the data space). More generally, this line of reasoning suggests that it would be interesting to investigate the features of a model that affect the quality of the Fisher Information Approximation.

B.2 Posterior predictive checks

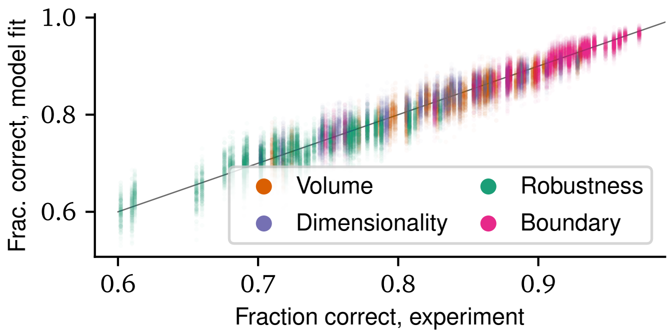

Figure B.2

Posterior predictive check for human performance on the generative task. We sampled 240 samples from the posterior over model parameters by thinning the MCMC chains used for model inference. For each of these samples, we ran a simulation of the experiment using the actual stimuli shown to the participants, and we recorded the resulting performance of all 202 simulated participants. This procedure yielded 240 samples of the joint posterior-predictive distribution of task performance over all participants. To visualize this distribution, for each participant we plotted a cloud of 240 dots, where the y coordinate of each dot is the simulated performance of that participant in one of the simulations, and the x coordinate is the true performance of that participant in the experiment plus a small random jitter (for ease of visualization). The gray line is the identity, showing that our inference procedure captures well the behavioral patterns in the experimental data. Colors indicate different task types, as indicated.

We performed a simple posterior predictive check (Kruschke, 2015) to ensure that the Bayesian hierarchical model described in the text captures the main pattern of behavior across our participants. In Figure B.2, the behavioral performance of the participants is compared with its posterior predictive distribution under the model, for the case of the human participants in the generative task. As can be seen from the figure, the performance of each participant is correctly captured by the model, across systematic differences between task types (with participants performing better in the boundary task variant than the robustness task variant, for instance) as well as individual differences between participants that performed the same task variant.

B.3 Details on raw estimated sensitivities

Table B.3 reports the posterior mean and standard deviation of the population-level parameters entering the regression (Equation A106). Note that these are the raw parameters, not their normalized counterparts relative to the likelihood sensitivity as reported in the rest of the paper.

B.4 Uncertainty in participant-level sensitivities

Figure B.4 illustrates the uncertainty in the estimate for the relative sensitivity of each participant. This uncertainty is typically small compared to between-participant variability of the sensitivity, which is therefore not a trivial consequence of the noise in the sensitivity estimation for individual participants.

bioRxiv preprint doi: https://doi.org/10.1101/2023.01.10.523479; this version posted March 16, 2025. The copyright holder for this preprint (which was not certified by peer review) is the author/funder, who has granted bioRxiv a license to display the preprint in perpetuity. It is made available under aCC-BY 4.0 International license.

	Generative task		Max likelihood task	
Parameter	Humans	ANNs	Humans	ANNs
μ_α (up/down bias)	0.107 ± 0.023	-0.242 ± 0.151	0.056 ± 0.024	0.010 ± 0.07
μ_L (likelihood)	0.461 ± 0.012	6.529 ± 0.188	0.561 ± 0.018	7.966 ± 0.401
μ_D (dimensionality)	2.150 ± 0.445	8.484 ± 0.653	1.285 ± 0.231	-0.030 ± 0.486
μ_B (boundary)	0.518 ± 0.045	6.286 ± 0.186	0.499 ± 0.058	0.883 ± 0.121
μ_V (volume)	0.108 ± 0.057	6.089 ± 0.204	0.105 ± 0.044	0.128 ± 0.196
μ_R (robustness)	1.018 ± 0.056	4.882 ± 0.417	1.276 ± 0.085	0.356 ± 0.281

Table B.3

Posterior mean±standard deviation for population-level parameters. See Equation A106 to Equation A114 for the precise definition of each parameter and its role in the hierarchical model of behavior.

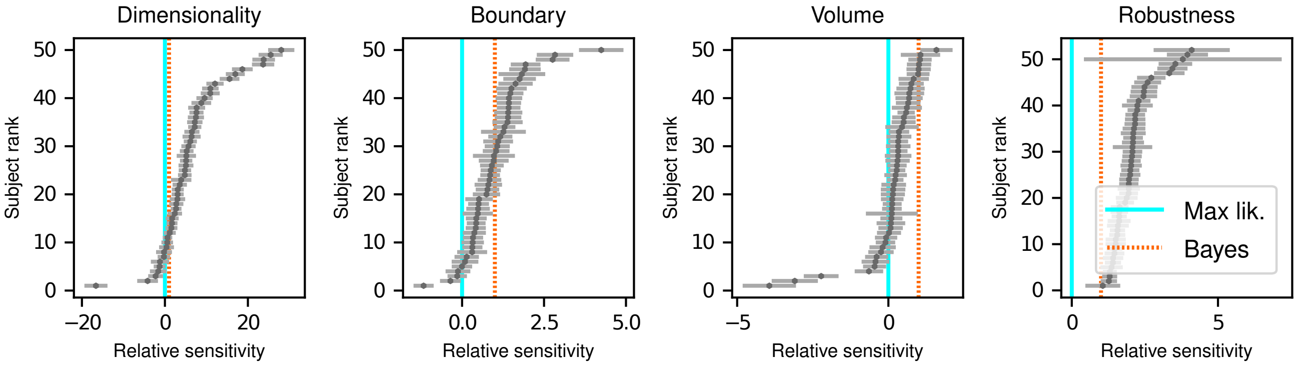

Figure B.4

Participant-level relative sensitivities to the geometric features that determine model complexity. Dots with error bars: posterior mean ± standard deviation of the relative sensitivity (the dots are the same as in Figure 3c). For ease of visualization, participants are ranked based on their posterior mean.

B.5 Lapse-rate analysis

To ensure that our findings were not sensitive to possible lapses in attention by the participants, we used the model variant described in Section A.6.1 to estimate a lapse rate for each participant simultaneously with the sensitivity parameters (Figure B.5). There was a substantial spread of lapse rates in the range 0–0.2 but no clear relationship between lapse rates and sensitivity. The sensitivity parameters estimated with this extended model were qualitatively compatible with those presented everywhere else in the text.

B.6 Outcome of significance tests specified in the preregistration documents

B.6.1 Formal comparison between ideal observers

We compared the Bayesian hierarchical model described in section A.6 to a simpler model, where participants were assumed to be sensitive only to likelihood differences, or in other words to choose $\mathcal{M}_1$ over $\mathcal{M}_2$ based only on which model was on average closer to the dot cloud constituting the stimulus on a given trial. Mathematically, this "likelihood-only" model was equivalent to fixing all β parameters to zero except for β_L in the model described in section A.6. All other details of the model were the same, and in particular the model still had a hierarchical structure with adaptive shrinkage (the participant-level parameters α and β_L were modeled as coming from Student T distributions controlled by population-level parameters). We compared the full model and the likelihood-only model on our human behavior data using WAIC (Gelman et al., 2014). This comparison indicated strong evidence in favor of the full model for both the generative task (Table B.6) and the maximum-likelihood task (Table B.7).

bioRxiv preprint doi: https://doi.org/10.1101/2023.01.10.523479; this version posted March 16, 2025. The copyright holder for this preprint (which was not certified by peer review) is the author/funder, who has granted bioRxiv a license to display the preprint in perpetuity. It is made available under aCC-BY 4.0 International license.

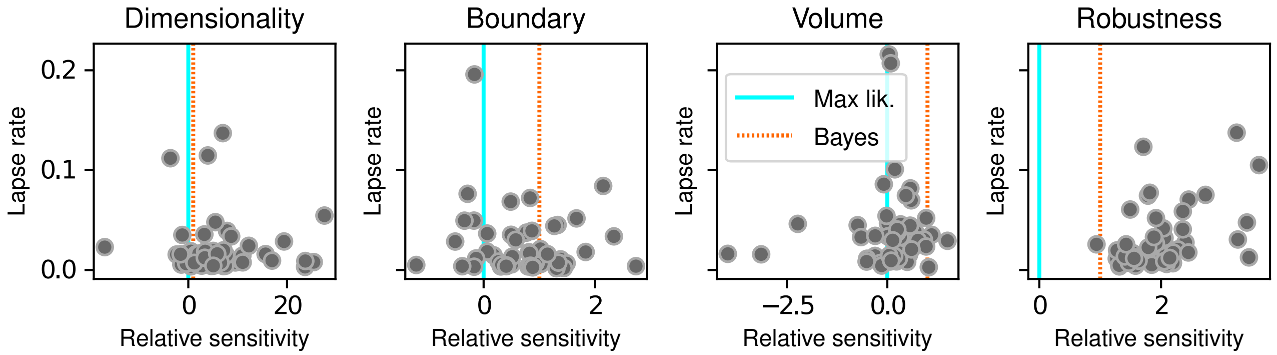

Figure B.5

Lapse rate versus relative sensitivity to complexity across participants. Each dot gives the posterior mean estimate of the relative sensitivity to one of the features that determine model complexity (abscissa) and the posterior mean estimate of the lapse rate, as defined in Section A.6.1.

Table B.6

Model	Rank	WAIC	pWAIC	dWAIC	SE	dSE
Full	0	-34824.6	641.963	0	183.981	0
Likelihood only	1	-37524.9	370.340	2700.4	183.923	69.3817

WAIC comparison of the full model and the likelihood-only model for human performance on the generative task, reported in the standard format used by McElreath (2016, section 6.4.2). WAIC is the value of the criterion (log-score scale, where higher is better), pWAIC is the estimated effective number of parameters, dWAIC is the difference between the WAIC of the given model and the highest-ranked one, SE is the standard error of the WAIC estimate, and dSE is the standard error of the difference in WAIC. These estimates were produced with the `compare` function provided by ArviZ (Kumar et al., 2019), using 12 MCMC chains with 10000 samples each for each model (in total, 120,000 samples for each model).

B.6.2 Other statistical tests

As described in the preregistration documents (Piasini et al., 2020, 2021b, 2022), in this work we have emphasized parameter estimation and information criteria-based model comparison over null hypothesis significance testing (see for instance McElreath (2016), and Kruschke (2015) for a discussion and comparison of these ideas). However, for completeness, we report in Table B.8 (1) the comparison between the Regions of Practical Equivalence (ROPE, Kruschke (2015)) and the 95% highest-density interval (HDI) for each population-level parameter, and (2) the "probability of direction" (Makowski, Ben-Shachar, Chen, & Lüdecke, 2019; Makowski, Ben-Shachar, & Lüdecke, 2019) for the same parameters (see below for more details on these methods). The ROPE-HDI tests highlight that the null value of zero sensitivity is not credible (rejected) for L, D and R, and neither rejected not accepted for V. The probability of direction is high for all parameters, including V, which has $PD = 0.97$ for the generative task and $PD = 0.99$ for the maximum-likelihood task. Overall, these analyses point to a significant sensitivity for all terms of the FIA in both experiments (generative and maximum-likelihood), with V having a more moderate effect size than the other terms.

Technical details on the ROPE-HDI comparison and on the Probability of Direction for sensitivity parameters. Briefly, the ROPE for a parameter is the range around a null value for that parameter such that variations within this range would imply only a "negligible change" in the behavior of the model, if all other parameters were held at their null values. The HDI is the smallest interval that contains a certain probability mass for the posterior of that parameter. The ROPE-HDI comparison is based on the idea that if the bulk of the posterior distribution for that parameter (represented by the HDI) falls outside the ROPE, then the null value for that parameter can be considered not credible (rejected). On the other hand, if the bulk of the posterior for the parameter falls within the ROPE, the null value can be considered credible (accepted). Finally, if the posterior distribution has a partial overlap with the ROPE (neither mostly contained within it, nor mostly falling outside of

bioRxiv preprint doi: https://doi.org/10.1101/2023.01.10.523479; this version posted March 16, 2025. The copyright holder for this preprint (which was not certified by peer review) is the author/funder, who has granted bioRxiv a license to display the preprint in perpetuity. It is made available under aCC-BY 4.0 International license.

Model	Rank	WAIC	pWAIC	dWAIC	SE	dSE
Full	0	-31022.8	638.926	0	184.912	0
Likelihood only	1	-33155.1	374.023	2132.28	186.667	63.1851

Table B.7

Same as Table B.6, for the maximum-likelihood task, where participants were asked to report the model that was closest to the data.

Parameter	ROPE	95% HDI (generative)	PD (generative)	95% HDI (max lik.)	PD (max lik.)
Likelihood	$\lvert\beta_L\rvert < 0.0076$	$[0.012, 0.437]$	1.00	$[0.526, 0.597]$	1.00
Dimensionality	$\lvert\beta_D\rvert < 0.43$	$[1.299, 3.048]$	1.00	$[0.835, 1.745]$	1.00
Boundary	$\lvert\beta_B\rvert < 0.06$	$[0.43, 0.604]$	1.00	$[0.386, 0.612]$	1.00
Volume	$\lvert\beta_V\rvert < 0.091$	$[-0.005, 0.218]$	0.97	$[0.019, 0.193]$	0.99
Robustness	$\lvert\beta_R\rvert < 0.11$	$[0.908, 1.126]$	1.00	$[1.11, 1.446]$	1.00

Table B.8

HDI vs ROPE comparison and Probability of Direction (PD) for the population-level parameters in the human experiments. See Supplementary Information section B.6.2 and Kruschke (2015) for an explanation of the ROPE-HDI comparison, and Makowski, Ben-Shachar, Chen, and Lüdecke (2019) and Makowski, Ben-Shachar, and Lüdecke (2019) for more details on the probability of direction metric. Note that the ROPE and HDI definitions were preregistered (Piasini et al., 2020, 2021b, 2022).

it), then the test is inconclusive. Note that, just like frequentist null hypothesis significance testing procedures and unlike the information criterion approach used above, this method depends on some arbitrary assumptions, namely the definition of the ROPE and the probability to use in computing the HDI.

In practice (for details, see our preregistration documents (Piasini et al., 2020, 2021b, 2022)), here we define, conventionally, the HDI as the smallest interval that contains 95% of the posterior. The ROPE is computed as follows. We start by defining a "negligible change" over the probability of the choice variable over the "main range" $[\mu_x - 2\sigma_x, \mu_x + 2\sigma_x]$ of one of the predictors in our model (L, D, B, V, or R). In other word, pick an interval of probabilities $[\pi_0 - \delta, \pi_0 + \delta]$ such that if the probability stays within $[\pi_0 - \delta, \pi_0 + \delta]$ when x varies over its typical range, then the probability is not meaningfully affected by x. Mathematically, if the probability of choosing one of the alternatives in the task is π and the log-odds is $\text{logit}(\pi) = \log(\pi/(1-\pi)) =$, then in a logistic regression setting

$$\text{logit}(\pi) = \alpha + \beta x \quad . \tag{B1}$$

If $\pi_0 = \text{logit}^{-1}(\alpha)$, then the ROPE for β is defined as

$$\text{logit}(\pi_0 + \delta) = \alpha + \beta_+(\mu_x + 2\sigma_x) \tag{B2}$$
$$\text{logit}(\pi_0 - \delta) = \alpha + \beta_-(\mu_x - 2\sigma_x) \tag{B3}$$

so that

$$\beta_+ = \frac{\text{logit}(\pi_0 + \delta) - \alpha}{\mu_x + 2\sigma_x} \tag{B4}$$
$$\beta_- = \frac{\text{logit}(\pi_0 - \delta) - \alpha}{\mu_x - 2\sigma_x} \tag{B5}$$

In our case, assuming a negligible influence of the up/down bias (α in Equation A106), $\pi_0 = 0.5$, and therefore we can assume $\alpha = 0$. The definition of the ROPE further depends on the arbitrary choice of δ, and on the values of μ_x and σ_x. We choose $\delta = 0.025$, and we estimated μ_x and σ_x by generating 25,000 experimental trials per task type (Dimensionality, Boundary, Volume, Robustness) and computing the empirical average and standard deviation of the predictors over that trial set. These numbers were all fixed at preregistration time (Piasini et al., 2020, 2021b, 2022).

bioRxiv preprint doi: https://doi.org/10.1101/2023.01.10.523479; this version posted March 16, 2025. The copyright holder for this preprint (which was not certified by peer review) is the author/funder, who has granted bioRxiv a license to display the preprint in perpetuity. It is made available under aCC-BY 4.0 International license.

B.7 Further details and results on the Noise-Integration-Noise observer

B.7.1 Estimation of the simplicity bias in the NIN observer

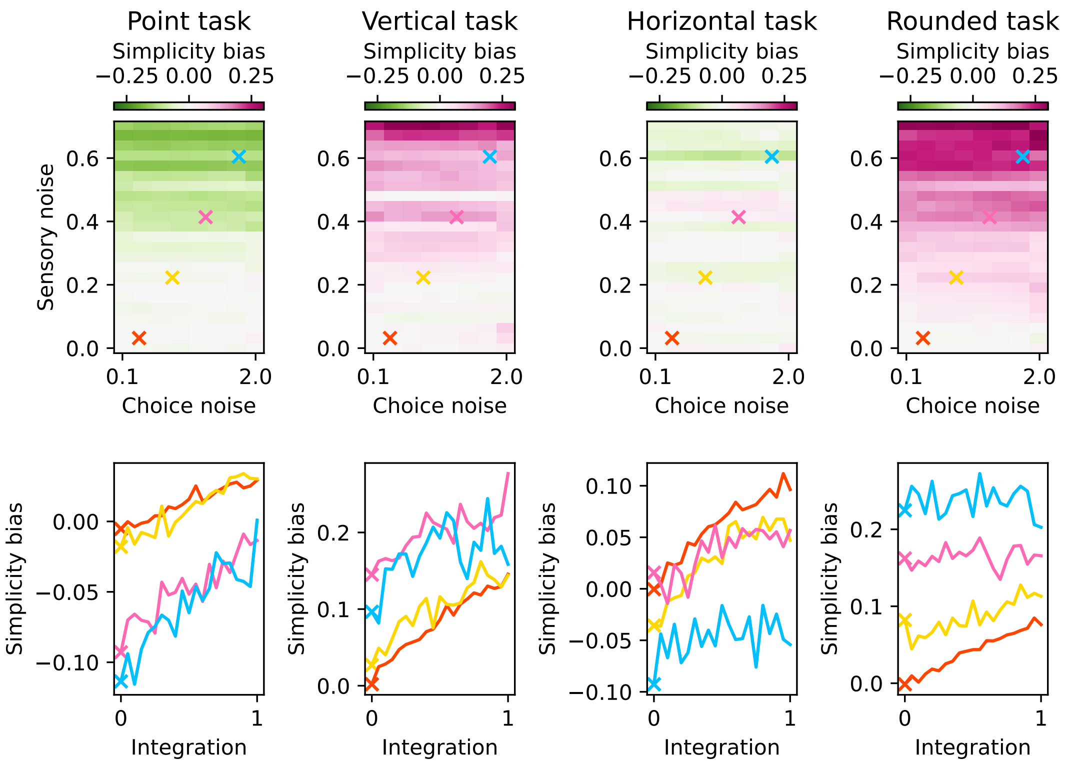

Figure B.9

Model-free estimate of simplicity bias in the Noise-Integration-Noise (NIN) observer, as a function of the observer's parameters, for each of the four task types (Dimensionality, Boundary, Volume, and Robustness; different columns show results for different task types). The example in Figure 2b in the main text corresponds to the Dimensionality task type. Top: simplicity bias as a function of sensory noise ρ and choice noise T, when the integration parameter b is set to 0, meaning that the observer does not integrate over latent causes (see section A.1). Note that the grid of choice noise values tested in the figure is not equally spaced; the values of T shown here are [0.10, 0.50, 0.56, 0.63, 0.71, 0.83, 1.00, 2.00], which correspond to the following values for the inverse temperature $1/T$: [10, 2.0, 1.8, 1.6, 1.4, 1.2, 1.0, 0.5]. Bottom: simplicity bias as a function of the integration parameter b, with sensory noise and choice noise fixed to the values indicated in the top panels with the colored crosses. Note how integration is the only parameter that is associated with consistent changes in the simplicity bias for all task types (increasing integration increases the simplicity bias). Sensory noise has inconsistent effects across task types, and choice noise does not affect the simplicity bias.

To estimate the magnitude and source(s) of simplicity biases exhibited by the Noise-Integration-Noise (NIN) observer, for a given configuration of the observer we started by simulating 10000 trials for each task type. Using this simulated data, we first quantified the bias in a model-free way, following the procedure outlined in A.1.1. This analysis showed that integration, and not sensory or choice noise, is the only parameter that is associated with consistent changes in the simplicity bias for all task types (Figure B.9).

We then measured the sensitivity of the NIN observer to the terms of the FIA expansion. To do so, we fitted to the simulated data a simplified version of the model we used for humans and artificial neural networks, described in detail in A.6. Briefly, the log odds for the observer to choose model $\mathcal{M}_1$ over $\mathcal{M}_2$ was taken to be similar to Equation A106:

$$\log \frac{p(\text{report } \mathcal{M}_1|X)}{p(\text{report } \mathcal{M}_2|X)} = \beta_L(L_2 - L_1) + \beta_D(D_2 - D_1) + \beta_B(B_2 - B_1) + \beta_V(V_2 - V_1) + \beta_R(R_2 - R_1)$$

where L_i, D_i, B_i, V_i and R_i are the FIA expansion terms for model $\mathcal{M}_i$, and β_k are parameters to be fitted, with,

bioRxiv preprint doi: https://doi.org/10.1101/2023.01.10.523479; this version posted March 16, 2025. The copyright holder for this preprint (which was not certified by peer review) is the author/funder, who has granted bioRxiv a license to display the preprint in perpetuity. It is made available under aCC-BY 4.0 International license.

for instance, β_D representing the sensitivity of the observer to model dimensionality. We fit this expression to the behavior of the observer in the 10000 simulated trials using a regularized logistic regression:

$$\begin{aligned} \beta_k &\sim \text{Normal}(\mu = 0, \sigma = 3) \\ C_t &\sim \text{Bernoulli}\left(\text{logit}^{-1}(\beta_L(L_2 - L_1) + \beta_D(D_2 - D_1) \right. \\ &\quad \left. + \beta_B(B_2 - B_1) + \beta_V(V_2 - V_1) + \beta_R(R_2 - R_1))\right) \end{aligned}$$

where C_t is the choice of the observer on model t, with $C_t = 1$ indicating that the observer chooses to report model $\mathcal{M}_1$ on that trial. For each NIN configuration tested, we report in Figure B.10 the maximum a posteriori (MAP) fit of the β values, obtained using the `find_MAP` function in PyMC (Salvatier et al., 2016). Our results were broadly consistent to those in Figure B.9, showing that integration was the only observer parameter with a consistent effect on FIA term sensitivity (Figure B.10, top and middle). Finally, we normalized the FIA term sensitivity by the likelihood sensitivity, in the same way as we did in the main text for human and artificial neural network observers. This analysis showed an even stronger qualitative match between the model-free and the FIA-based quantification of the simplicity bias (compare Figure B.9 and Figure B.10, bottom).

B.7.2 Human simplicity preference is compatible with integration over latent causes

Fits of the Noise-Integration-Noise (NIN) model indicated that the behavior of most of our human participants was consistent with integrating over latent causes. Specifically, for thefor the Robustness and Dimensionality versions of the generative task most participants had their "integration indices" b pinned at 1, which is the maximum possible value and indicated integration over the full statistical manifolds (Figure B.11b). For the Volume and Boundary tasks, some participants failed to integrate ($b = 0$) but the others showed a range of propensities toward integration. These results are consistent with the sensitivities extracted with the FIA analysis (Table B.3), which showed that participants were generally more strongly sensitive to dimensionality and robustness than volume and boundary.

The distribution of best-fitting values of the sensory noise parameter ρ (Figure B.11a) exhibited two components: 1) a spike at zero, indicating a number of participants with negligible sensory noise; and 2) a separate, broader peak. This peak was centered around an "efficent" value, as we will now argue. As a reminder, on each trial of the experiment we showed a cloud of $N = 10$ dots to the participants, with each dot being sampled from a 2D Gaussian distribution with isotropic variance and standard deviation $\sigma = 1$. For our task, the location of the cloud centroid $\bar{x} = (\sum_{i=1}^{N} x_i)/N$ is a sufficient statistic, in the sense that it is enough to compute the posterior probabilities for our behavioral models (both FIA and NIN). The standard deviation of the sampling distribution for $\bar{x}$ (its standard error) therefore captures the intrinsic reliability of the raw visual information provided to the participants, before it is corrupted by the sensory noise $\eta \sim \mathcal{N}(0, \rho)$. From the definition of $\bar{x}$, its standard error is simply $\sigma/\sqrt{N} = 0.36$. The variance of the perceived centroid location $\tilde{x}$ is then $\sigma^2/N + \rho^2$, which is dominated by ρ^2 when $\rho > \sigma/\sqrt{N}$ and by $\sigma/\sqrt{N}$ when $\rho < \sigma/\sqrt{N}$. Therefore, $\rho = \sigma/\sqrt{N}$ is the "efficient" value of ρ, corresponding to the maximum spatial resolution beyond which it is not convenient to encode the location of the centroid due to diminishing returns. This value is reported in Figure B.11a as an arrow, showing that the estimated values of ρ for the participants with non-negligible sensory noise are centered around this value.

The best-fitting inverse-temperature parameters have a distribution with two components (Figure B.11c): 1) a bulk below 1, and 2) a tail with values that go far above 1. As a reminder, for this parameter, 0 corresponds to making random choices, 1 corresponds to sampling from the posterior over models (computed by integrating in a neighborhood of the max likelihood point whose radius is determined by the "integration" parameter b), and infinity corresponds to selecting deterministically the model with the largest posterior. Also recall from A.7 that the inverse temperature β is bounded in the fitting procedure to the interval $[0, 10]$, so the participants with $\beta = 10$ could possibly be fit even better by larger values. However, in practice this constraint does not make a large difference with respect to the behavior of the fitted model, as $\beta = 10$ can be already considered close enough to a deterministic choice regime. The peak of the inverse-temperature distribution is very close to the mean posterior estimate for the (population-level) sensitivity to likelihood in our FIA model (which is 0.46 in the generative task, plotted as a dashed cyan line in Figure B.11c). This result is consistent with the idea that the likelihood sensitivity captures an overall scaling of the slope of the psychometric function, and further supports our choice to focus on normalized sensitivities in the main text.

Applying the same analysis to the data from the maximum-likelihood task gives broadly consistent results, with the exception that the inferred degree of integration is less peaked around 1 and much more distributed over a

bioRxiv preprint doi: https://doi.org/10.1101/2023.01.10.523479; this version posted March 16, 2025. The copyright holder for this preprint (which was not certified by peer review) is the author/funder, who has granted bioRxiv a license to display the preprint in perpetuity. It is made available under aCC-BY 4.0 International license.

continuum between 0 and 1 for all tasks (Figure B.12). This result implies that the participants still performed integration under these conditions, albeit to a lesser extent than for the generative task, corroborating the results of the FIA analysis in Figure 5 in the main text.

B.7.3 Model comparison between NIN and FIA

We checked whether our more refined, theory-driven behavioral model based on the FIA explained participant behavior better than the more elementary NIN model. To do so in a fair way, we re-fit the FIA model on a individual-participant basis (that is, we removed the hierarchical structure) using maximum likelihood, with the same optimization algorithm described for the NIN model in A.7. This procedure allowed us to compute an Akaike Information Criterion for each model on each participant. Figure B.11b shows that the the vast majority of participants (182 out of 202) were better described by the FIA model.

References

Gelman, A., Carlin, J. B., Stern, H. S., Dunson, D. B., Vehtari, A., & Rubin, D. B. (2014). *Bayesian Data Analysis* (3rd ed.). CRC Press.

Kruschke, J. K. (2015). *Doing Bayesian Data Analysis* (2nd ed.). Academic Press.

Kumar, R., Carroll, C., Hartikainen, A., & Martin, O. (2019). ArviZ a unified library for exploratory analysis of Bayesian models in Python. *Journal of Open Source Software*, *4*(33), 1143. https://doi.org/10.21105/joss.01143

Makowski, D., Ben-Shachar, M. S., Chen, S. H. A., & Lüdecke, D. (2019). Indices of Effect Existence and Significance in the Bayesian Framework. *Frontiers in Psychology*, *10*. Retrieved December 11, 2022, from https://www.frontiersin.org/articles/10.3389/fpsyg.2019.02767

Makowski, D., Ben-Shachar, M. S., & Lüdecke, D. (2019). bayestestR: Describing Effects and their Uncertainty, Existence and Significance within the Bayesian Framework. *Journal of Open Source Software*, *4*(40), 1541. https://doi.org/10.21105/joss.01541

McElreath, R. (2016). *Statistical Rethinking*. CRC Press.

Piasini, E., Balasubramanian, V., & Gold, J. I. (2020). *Preregistration document*. https://doi.org/10.17605/OSF.IO/2X9H6

Piasini, E., Balasubramanian, V., & Gold, J. I. (2021a). Effect of Geometric Complexity on Intuitive Model Selection. *The First International Symposium on AI and Neuroscience - ACAIN 2021*.

Piasini, E., Balasubramanian, V., & Gold, J. I. (2021b). *Preregistration document addendum*. https://doi.org/10.17605/OSF.IO/5HDQZ

Piasini, E., Liu, S., Balasubramanian, V., & Gold, J. I. (2022). *Preregistration document addendum*. https://doi.org/10.17605/OSF.IO/826JV

Salvatier, J., Wiecki, T. V., & Fonnesbeck, C. (2016). Probabilistic programming in Python using PyMC3. *PeerJ Computer Science*, *2*, e55. https://doi.org/10.7717/peerj-cs.55

bioRxiv preprint doi: https://doi.org/10.1101/2023.01.10.523479; this version posted March 16, 2025. The copyright holder for this preprint (which was not certified by peer review) is the author/funder, who has granted bioRxiv a license to display the preprint in perpetuity. It is made available under aCC-BY 4.0 International license.

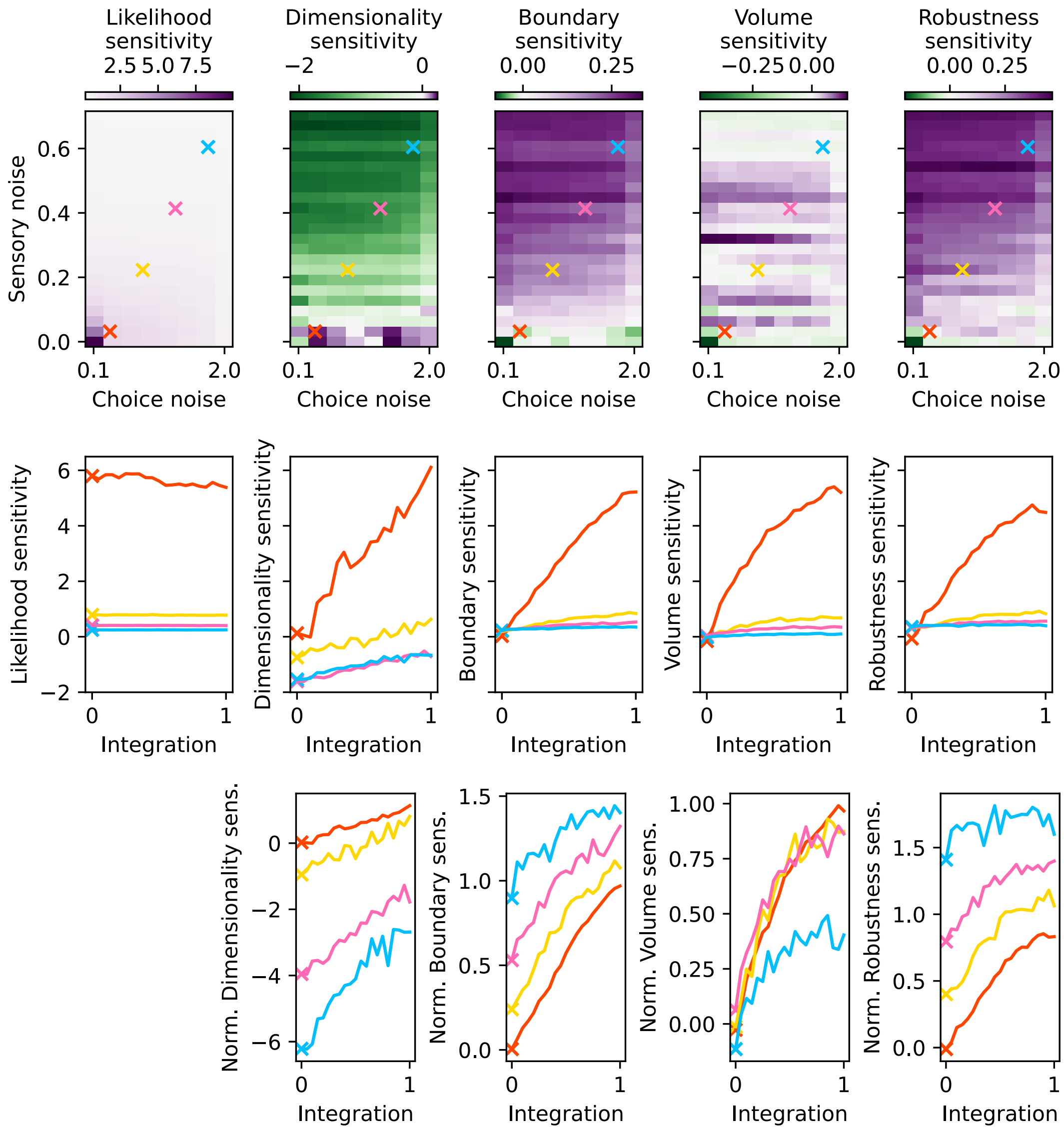

Figure B.10

Sensitivity to model likelihood and to the geometric features that characterize model complexity, for the Noise-Integration-Noise observer, as a function of the parameters of the observer. The parameter values tested are the same as in Figure B.9. Top: dependence of the sensitivities on the sensory noise σ and the choice noise T, when the integration parameter b is fixed to zero (meaning that the observer does not integrate over latent causes). Middle: dependence on the sensitivities on integration, when sensory and choice noise are fixed to the values indicated by the colored crosses in the top panels. Bottom: same as middle, but for the normalized sensitivities, obtained by dividing the raw sensitivities by the likelihood sensitivity. Note that, reflecting the results in Figure B.9, the parameter controlling integration (x axis on each individual subplot) is the only one that has a consistent effect on the sensitivity to all features, generally increasing it. Note also the qualitative match between the bottom panels here and those in Figure B.9. The agreement with the data presented here and that in Figure B.9 further confirms that our theory-driven approach captures the intuitive notion of simplicity bias in this task.

bioRxiv preprint doi: https://doi.org/10.1101/2023.01.10.523479; this version posted March 16, 2025. The copyright holder for this preprint (which was not certified by peer review) is the author/funder, who has granted bioRxiv a license to display the preprint in perpetuity. It is made available under aCC-BY 4.0 International license.

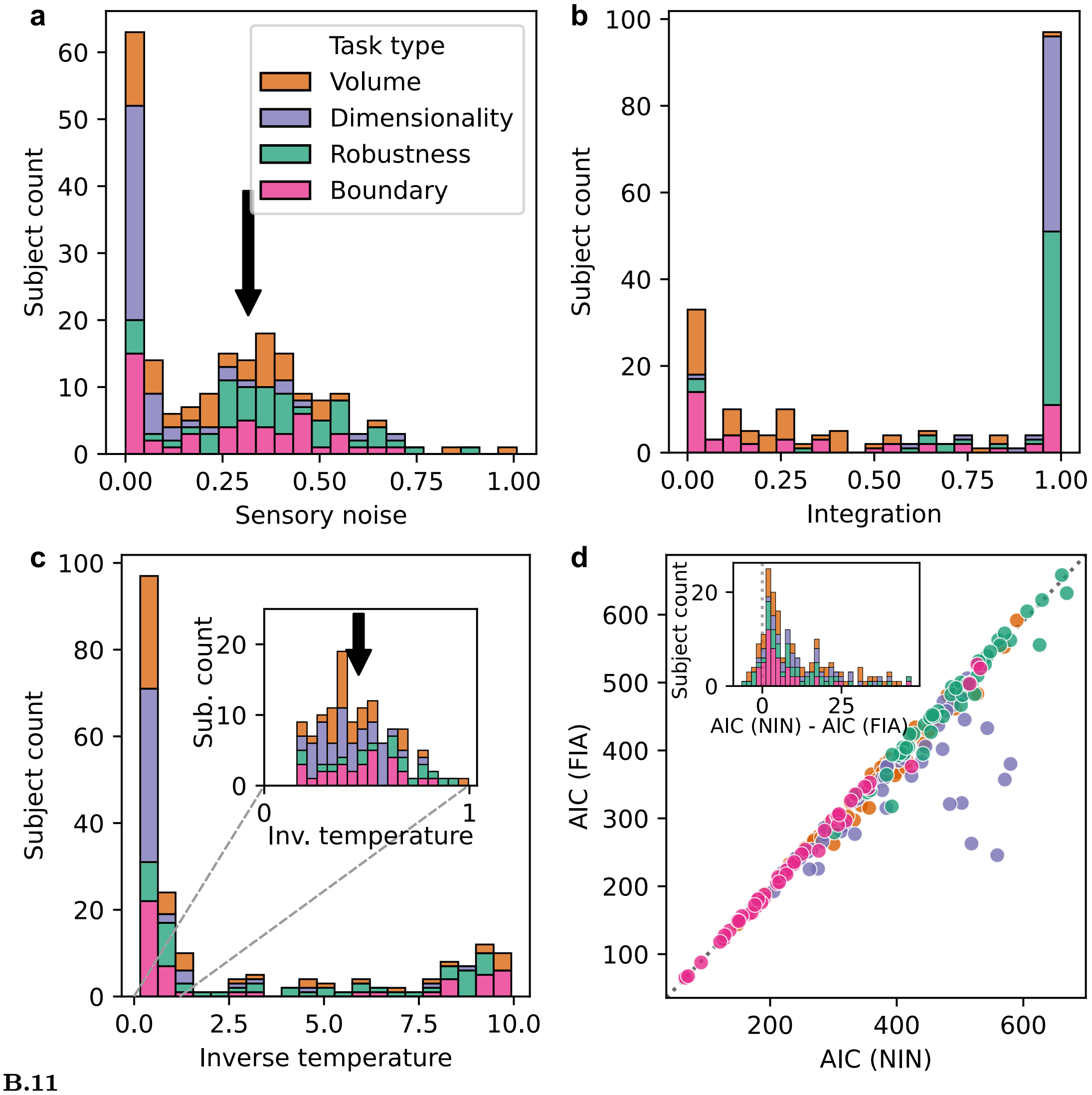

Figure B.11

Analysis of human behavior on the generative task, using the Noise-Integration-Noise (NIN) model. ***a:*** *sensory noise (ρ) estimate for all participants, broken down by task type (colors). Arrow: standard error of the location of the centroid of the dot cloud that, on any trial, represented the data X shown to the participants ($\sigma/\sqrt{N} = 0.32$, using the notation of A.3 and A.4.2).* ***b:*** *estimates of integration parameter b.* ***c:*** *estimates of inverse temperature of the choice noise $\beta = 1/T$. Inset: detail of the inverse temperature histogram for $\beta \in [0, 1]$. Arrow: numerical value of the population estimate of likelihood sensitivity from the FIA model.* ***d:*** *simple participant-level model comparison (Akaike Information Criterion) between the NIN model and the behavioral model based on the FIA (Equation A106). Lower is better; the dashed diagonal line is the identity. Inset: histogram of NIN-FIA difference, excluding outliers with large positive values, which are overwhelmingly better described by FIA. The AIC is lower (better) for the FIA than for the NIN model for 182 out of 201 subjects.*

bioRxiv preprint doi: https://doi.org/10.1101/2023.01.10.523479; this version posted March 16, 2025. The copyright holder for this preprint (which was not certified by peer review) is the author/funder, who has granted bioRxiv a license to display the preprint in perpetuity. It is made available under aCC-BY 4.0 International license.

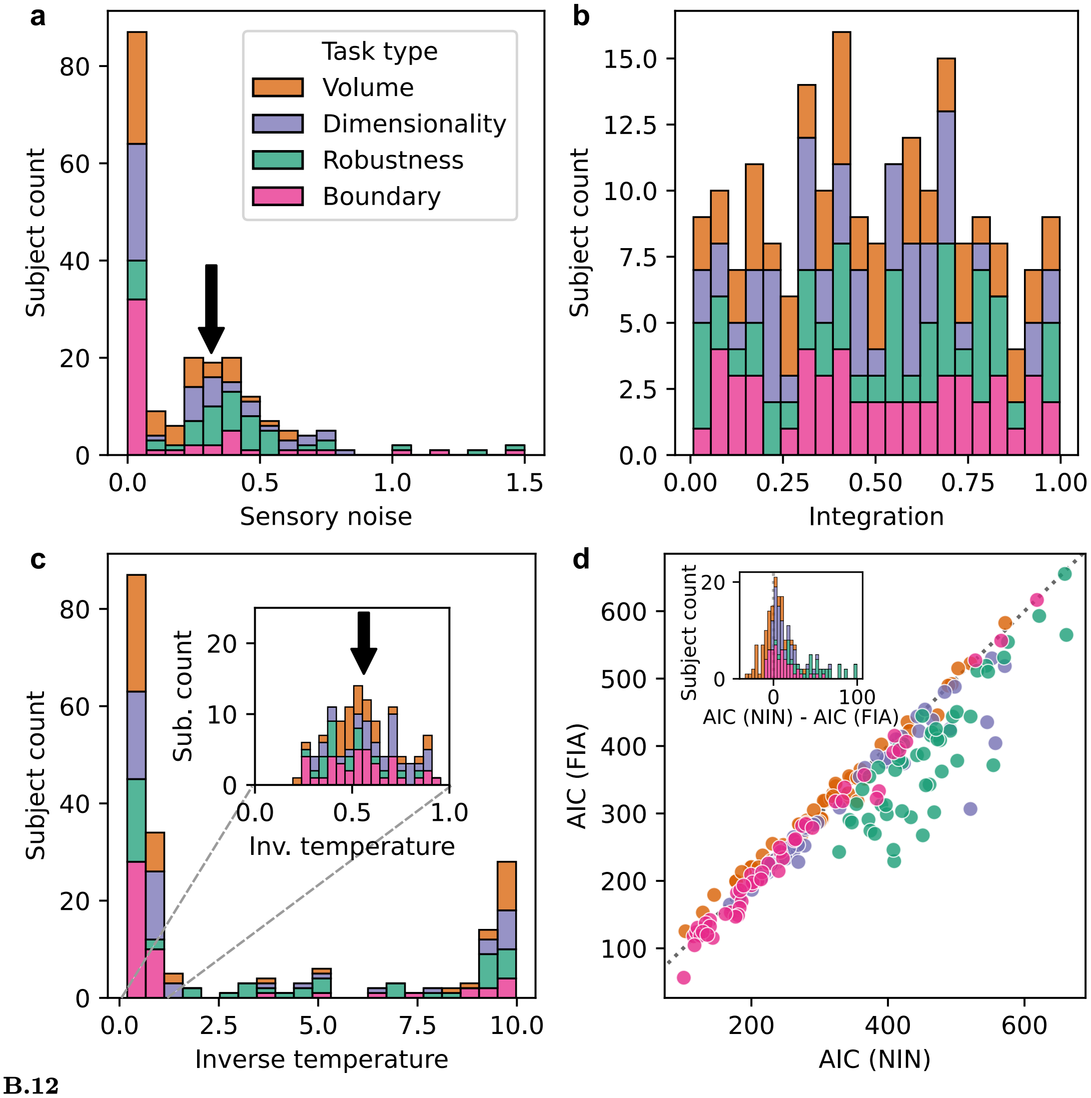

Figure B.12

Analysis of human behavior on the maximum-likelihood task, using the Noise-Integration-Noise (NIN) model. Same as Figure B.11, but for the behavioral data of the subjects that performed the maximum-likelihood task. In panel d, the AIC is lower (better) for the FIA than for the NIN model for 144 out of 201 subjects.
