# Supplementary material for: How Occam’s razor guides human decision-making
Source: bioRxiv. 2023 Feb 8:2023.01.10.523479. Originally published 2023 Jan 10. Preprint. [Version 2] doi: 10.1101/2023.01.10.523479 (PMC9882019; doi:10.1101/2023.01.10.523479)
Supplement: Supplementary file 1 [file NIHPP2023.01.10.523479v2-supplement-1.pdf]

bioRxiv preprint doi: https://doi.org/10.1101/2023.01.10.523479; this version posted February 8, 2023. The copyright holder for this preprint (which was not certified by peer review) is the author/funder, who has granted bioRxiv a license to display the preprint in perpetuity. It is made available under aCC-BY 4.0 International license.

Supplementary Materials

A Methods **14**
A.1 Derivation of the boundary term in the Fisher Information Approximation 14
A.2 Behavioral experiments with human subjects 20
A.3 Detailed model definitions and computation of FIA terms 21
A.4 Numerical experiments with artificial neural networks 27
A.5 Experimental data analysis 29

B Supplementary Figures and Tables **32**

C Supplementary Text **41**
C.1 Numerical comparison of the extended FIA vs exact Bayes 41
C.2 Subject demographics 41
C.3 Posterior predictive checks 41
C.4 Details on raw estimated sensitivities 41
C.5 Uncertainty in subject-level sensitivities 42
C.6 Lapse-rate analysis 42
C.7 Outcome of significance tests specified in the preregistration documents 42

References **43**

A Methods

A.1 Derivation of the boundary term in the Fisher Information Approximation

Here we generalize the derivation of the Fisher Information Approximation given by Balasubramanian (*11*) to the case where the maximum-likelihood solution for a model lies on the boundary of the parameter space. Apart from the more general assumptions, the following derivation follows closely the original one, with some minor notational changes. This derivation appeared in preliminary form in (*46*).

A.1.1 Set-up and hypotheses

The problem we consider here is that of selecting between two models (say $\mathcal{M}_1$ and $\mathcal{M}_2$), after observing empirical data $X = \{x_i\}_{i=1}^N$. N is the sample size and $\mathcal{M}_1$ is assumed to have d parameters, collectively indexed as ϑ taking values in a compact domain Θ. As a prior over ϑ we take Jeffrey's prior:

$$w(\vartheta) = \frac{\sqrt{\det g(\vartheta)}}{\int \mathrm{d}^d\vartheta \sqrt{\det g(\vartheta)}} \tag{1}$$

where g is the (expected) Fisher Information of the model $\mathcal{M}_1$:

$$g_{\mu\nu}(\vartheta) = \mathbb{E}\left[-\frac{\partial^2 \ln p(x|\vartheta)}{\partial\vartheta^\mu \partial\vartheta^\nu}\right]_\vartheta \tag{2}$$

The Bayesian posterior

$$\mathbb{P}(\mathcal{M}_1|X) = \frac{\mathbb{P}(\mathcal{M}_1)}{\mathbb{P}(X)} \int \mathrm{d}^d w(\vartheta)\mathbb{P}(X|\vartheta) \tag{3}$$

then becomes, after assuming a flat prior over models and dropping irrelevant terms:

$$\mathbb{P}(\mathcal{M}_1|X) = \frac{\int_\Theta \mathrm{d}^d\vartheta \sqrt{\det g} \exp\left[-N(-\frac{1}{N}\ln \mathbb{P}(X|\vartheta))\right]}{\int \mathrm{d}^d\vartheta \sqrt{\det g}} \tag{4}$$

Just as in (*11*), we now make a number of regularity assumptions: 1. $\ln \mathbb{P}(X|\vartheta)$ is smooth; 2. there is a unique global minimum $\hat{\vartheta}$ for $\ln \mathbb{P}(X|\vartheta)$; 3. $g_{\mu\nu}(\vartheta)$ is smooth; 4. $g_{\mu\nu}(\hat{\vartheta})$ is positive definite; 5. $\Theta \subset \mathbb{R}^d$ is compact; and 6. the values of the local minima of $\ln \mathbb{P}(X|\vartheta)$ are bounded away from the global minimum by some $\epsilon > 0$. Importantly, unlike in (*11*), we do not assume that $\hat{\vartheta}$ is in the interior of Θ.

bioRxiv preprint doi: https://doi.org/10.1101/2023.01.10.523479; this version posted February 8, 2023. The copyright holder for this preprint (which was not certified by peer review) is the author/funder, who has granted bioRxiv a license to display the preprint in perpetuity. It is made available under aCC-BY 4.0 International license.

The shape of Θ. Because we are specifically interested in understanding what happens at a boundary of the parameter space, we add a further assumption that, while being not very restrictive in spirit, allows us to derive a particularly interpretable result. In particular, we assume that Θ is specified by a single linear constraint of the form:

$$D_\mu \vartheta^\mu + d \geq 0 \tag{5}$$

Without loss of generality, we also take the constraint to be expressed in Hessian normal form, namely, $||D_\mu|| = 1$. For clarity, note this assumption on the shape of Θ is used only from subsubsection A.1.3 onward.

A.1.2 Preliminaries

We now proceed to set up a low-temperature expansion of Equation 4 around the saddle point $\hat{\vartheta}$. We start by rewriting the numerator in Equation 4 as:

$$\int_\Theta \mathrm{d}^d\vartheta \exp\left[-N\left(-\frac{1}{2N}\ln\det g - \frac{1}{N}\ln\mathbb{P}(X|\vartheta)\right)\right] \tag{6}$$

The idea of the Fisher Information Approximation is to expand the integrand in Equation 6 in powers of N around the maximum likelihood point $\hat{\vartheta}$. To this end, we define three useful objects:

$$\tilde{I}_{\mu_1\cdots\mu_i} := -\frac{1}{N}\nabla_{\mu_1}\cdots\nabla_{\mu_i}\ln\mathbb{P}(X|\vartheta)\Big|_{\hat{\vartheta}} = -\frac{1}{N}\sum_{j=1}^{N}\nabla_{\mu_1}\cdots\nabla_{\mu_i}\ln\mathbb{P}(x_j|\vartheta)\Big|_{\hat{\vartheta}}$$

$$F_{\mu_1\cdots\mu_i} := \nabla_{\mu_1}\cdots\nabla_{\mu_i}\ln\det g(\vartheta)\Big|_{\hat{\vartheta}}$$

$$\psi := -\frac{1}{2N}\ln\det g - \frac{1}{N}\ln\mathbb{P}(X|\vartheta)$$

We immediately note that:

$$\nabla_{\mu_1}\cdots\nabla_{\mu_i}\psi\Big|_{\hat{\vartheta}} = \tilde{I}_{\mu_1\cdots\mu_i} - \frac{1}{2N}F_{\mu_1\cdots\mu_i}$$

which is useful to compute

$$\begin{aligned}\psi(\vartheta) &= \psi(\hat{\vartheta}) + \nabla_\mu\psi\Big|_{\hat{\vartheta}}(\vartheta^\mu - \hat{\vartheta}^\mu) + \frac{1}{2}\nabla_\mu\nabla_\nu\psi\Big|_{\hat{\vartheta}}(\vartheta^\mu - \hat{\vartheta}^\mu)(\vartheta^\nu - \hat{\vartheta}^\nu) + \ldots \\ &= \sum_{i=0}^{\infty}\frac{1}{i!}\nabla_{\mu_1}\cdots\nabla_{\mu_i}\psi\Big|_{\hat{\vartheta}}(\vartheta^{\mu_1} - \hat{\vartheta}^{\mu_1})\cdots(\vartheta^{\mu_i} - \hat{\vartheta}^{\mu_i}) \\ &= \sum_{i=0}^{\infty}\frac{1}{i!}\nabla_{\mu_1}\cdots\nabla_{\mu_i}\psi\Big|_{\hat{\vartheta}}\prod_{k=1}^{i}(\vartheta^{\mu_k} - \hat{\vartheta}^{\mu_k})\end{aligned}$$

It is also useful to center the integration variables by introducing

$$\phi := \sqrt{N}(\vartheta - \hat{\vartheta}) \tag{7}$$

$$\mathrm{d}^d\phi = N^{d/2}\,\mathrm{d}^d\vartheta \tag{8}$$

so that

$$\nabla_{\mu_1}\cdots\nabla_{\mu_i}\psi\Big|_{\hat{\vartheta}}\prod_{k=1}^{i}(\vartheta^{\mu_k} - \hat{\vartheta}^{\mu_k}) = N^{-i/2}\left(\tilde{I}_{\mu_1\cdots\mu_i} - \frac{1}{2N}F_{\mu_1\cdots\mu_i}\right)\phi^{\mu_1}\cdots\phi^{\mu_i} \tag{9}$$

bioRxiv preprint doi: https://doi.org/10.1101/2023.01.10.523479; this version posted February 8, 2023. The copyright holder for this preprint (which was not certified by peer review) is the author/funder, who has granted bioRxiv a license to display the preprint in perpetuity. It is made available under aCC-BY 4.0 International license.

and Equation 6 becomes:

$$\begin{aligned}
\int \mathrm{d}^d\vartheta \exp[-N\psi] &= N^{-d/2}\int \mathrm{d}^d\phi \exp\left[-N\sum_{i=0}^{\infty}\frac{1}{i!}N^{-i/2}\left(\tilde{I}_{\mu_1\cdots\mu_i}-\frac{1}{2N}F_{\mu_1\cdots\mu_i}\right)\phi^{\mu_1}\cdots\phi^{\mu_i}\right]\\
&= N^{-d/2}\int \mathrm{d}^d\phi \exp\left\{-N\left(-\frac{1}{N}\ln\mathbb{P}(X|\hat{\vartheta})-\frac{1}{2N}\ln\det g(\hat{\vartheta})\right)+\right.\\
&\quad \left.-N\left[\sum_{i=1}^{\infty}\frac{1}{i!}N^{-i/2}\left(\tilde{I}_{\mu_1\cdots\mu_i}-\frac{1}{2N}F_{\mu_1\cdots\mu_i}\right)\phi^{\mu_1}\cdots\phi^{\mu_i}\right]\right\}\\
&= N^{-\frac{d}{2}}\exp\left[-\left(-\ln\mathbb{P}(X|\hat{\vartheta})-\frac{1}{2}\ln\det g(\hat{\vartheta})\right)\right]\times\\
&\quad \times\int \mathrm{d}^d\phi \exp\left\{-N\left[\frac{1}{\sqrt{N}}\tilde{I}_\mu\phi^\mu+\frac{1}{2N}\tilde{I}_{\mu\nu}\phi^\mu\phi^\nu+\right.\right.\\
&\quad \left.\left.+\frac{1}{N}\sum_{i=1}^{\infty}N^{-\frac{i}{2}}\left(\frac{1}{(i+2)!}\tilde{I}_{\mu_1\cdots\mu_{i+2}}\phi^{\mu_1}\cdots\phi^{\mu_{i+2}}-\frac{1}{2i!}F_{\mu_1\cdots\mu_i}\phi^{\mu_1}\cdots\phi^{\mu_i}\right)\right]\right\}
\end{aligned}$$

Therefore,

$$\begin{aligned}
\mathbb{P}(\mathcal{M}_1|X) &= N^{-\frac{d}{2}}\exp\left[-\left(-\ln\mathbb{P}(X|\hat{\vartheta})-\frac{1}{2}\ln\det g(\hat{\vartheta})+\ln\int \mathrm{d}^d\vartheta\sqrt{\det g}\right)\right]\times\\
&\quad\times\int \mathrm{d}^d\phi \exp\left[-\sqrt{N}\tilde{I}_\mu\phi^\mu-\frac{1}{2}\tilde{I}_{\mu\nu}\phi^\mu\phi^\nu+\right.\\
&\quad \left.-\sum_{i=1}^{\infty}N^{-\frac{i}{2}}\left(\frac{1}{(i+2)!}\tilde{I}_{\mu_1\cdots\mu_{i+2}}\phi^{\mu_1}\cdots\phi^{\mu_{i+2}}-\frac{1}{2i!}F_{\mu_1\cdots\mu_i}\phi^{\mu_1}\cdots\phi^{\mu_i}\right)\right]\Bigg\}\\
&= N^{-\frac{d}{2}}\exp\left[-\left(-\ln\mathbb{P}(X|\hat{\vartheta})-\frac{1}{2}\ln\det g(\hat{\vartheta})+\ln\int_\Theta \mathrm{d}^d\vartheta\sqrt{\det g}\right)\right]\cdot Q
\end{aligned} \tag{10}$$

where

$$Q = \int_\Phi \mathrm{d}^d\phi \exp\left[-\sqrt{N}\tilde{I}_\mu\phi^\mu-\frac{1}{2}\tilde{I}_{\mu\nu}\phi^\mu\phi^\nu-G(\phi)\right] \tag{11}$$

and

$$G(\phi)=\sum_{i=1}^{\infty}N^{-\frac{i}{2}}\left(\frac{1}{(i+2)!}\tilde{I}_{\mu_1\cdots\mu_{i+2}}\phi^{\mu_1}\cdots\phi^{\mu_{i+2}}-\frac{1}{2i!}F_{\mu_1\cdots\mu_i}\phi^{\mu_1}\cdots\phi^{\mu_i}\right) \tag{12}$$

where $G(\phi)$ collects the terms that are suppressed by powers of N.

Our problem has been now reduced to computing Q by performing the integral in Equation 11. Now our assumptions come into play for the key approximation step. For the sake of simplicity, assuming that N is large we drop $G(\phi)$ from the expression above, so that Q becomes a simple Gaussian integral with a linear term:

$$Q = \int_\Phi \mathrm{d}^d\phi \exp\left[-\sqrt{N}\tilde{I}_\mu\phi^\mu-\frac{1}{2}\phi^\mu\tilde{I}_{\mu\nu}\phi^\nu\right] \tag{13}$$

A.1.3 Choosing a good system of coordinates

Consider now the Observed Fisher Information at the maximum likelihood, $\tilde{I}_{\mu\nu}$. As long as it is not singular, we can define its inverse $\Delta^{\mu\nu}=(\tilde{I}_{\mu\nu})^{-1}$. If $\tilde{I}_{\mu\nu}$ is positive definite, then the matrix representation of $\tilde{I}_{\mu\nu}$ has a set of d positive eigenvalues, which we denote by $\{\sigma_{(1)}^{-2},\sigma_{(2)}^{-2},\dots,\sigma_{(d)}^{-2}\}$. The matrix representation of $\Delta^{\mu\nu}$ has eigenvalues $\{\sigma_{(1)}^{2},\sigma_{(2)}^{2},\dots,\sigma_{(d)}^{2}\}$, and is diagonal in the same choice of coordinates as $\tilde{I}_{\mu\nu}$. We denote by

bioRxiv preprint doi: https://doi.org/10.1101/2023.01.10.523479; this version posted February 8, 2023. The copyright holder for this preprint (which was not certified by peer review) is the author/funder, who has granted bioRxiv a license to display the preprint in perpetuity. It is made available under aCC-BY 4.0 International license.

U the (orthogonal) diagonalizing matrix; i.e., U is such that

$$U\Delta U^T = \begin{bmatrix} \sigma^2_{(1)} & 0 & \cdots & 0 \\ 0 & \sigma^2_{(2)} & & \vdots \\ \vdots & & \ddots & 0 \\ 0 & \dots & 0 & \sigma^2_{(d)} \end{bmatrix} \quad , \quad U^T U = U U^T = \mathbb{I} \tag{14}$$

We define also the matrix K as the product of the diagonal matrix with elements $1/\sigma_{(k)}$ along the diagonal and U:

$$K = \begin{bmatrix} 1/\sigma_{(1)} & 0 & \cdots & 0 \\ 0 & 1/\sigma_{(2)} & & \vdots \\ \vdots & & \ddots & 0 \\ 0 & \dots & 0 & 1/\sigma_{(d)} \end{bmatrix} U \tag{15}$$

Note that

$$\det K = (\det \Delta^{\mu\nu})^{-1/2} = \sqrt{\det \tilde{I}_{\mu\nu}}$$

and that K corresponds to a sphering transformation, in the sense that

$$K\Delta K^T = \mathbb{I} \quad \text{or} \quad K^\mu{}_\kappa \Delta^{\kappa\lambda} K^\nu{}_\lambda = \delta^{\mu\nu} \tag{16}$$

and therefore, if we define the inverse

$$P = K^{-1}$$

we have

$$P^T(\tilde{I}_{\mu\nu})P = \mathbb{I} \quad \text{or} \quad P^\kappa{}_\mu \tilde{I}_{\kappa\lambda} P^\lambda{}_\nu = \delta_{\mu\nu} \tag{17}$$

We can now define a new set of coordinates by centering and sphering, as follows:

$$\xi^\mu = K^\mu{}_\nu \left(\phi^\nu + \sqrt{N}\Delta^{\nu\kappa}\tilde{I}_\kappa\right) \tag{18}$$

Then,

$$\mathrm{d}^d\xi = \sqrt{\det \tilde{I}_{\mu\nu}}\, \mathrm{d}^d\phi \tag{19}$$

and

$$\phi^\mu = P^\mu{}_\nu \xi^\nu - \sqrt{N}\Delta^{\mu\nu}\tilde{I}_\nu \tag{20}$$

In this new set of coordinates,

$$\begin{aligned} -\sqrt{N}\tilde{I}_\nu\phi^\nu - \frac{1}{2}\phi^\mu\tilde{I}_{\mu\nu}\phi^\nu = \\ &= -\left(\sqrt{N}\tilde{I}_\nu + \frac{1}{2}\phi^\mu\tilde{I}_{\mu\nu}\right)\phi^\nu \\ &= -\left(\sqrt{N}\tilde{I}_\nu + \frac{1}{2}P^\mu{}_\kappa\xi^\kappa\tilde{I}_{\mu\nu}\frac{1}{2}\sqrt{N}\Delta^{\mu\kappa}\tilde{I}_\kappa\tilde{I}_{\mu\nu}\right)\phi^\nu \\ &= -\sqrt{N}\tilde{I}_\nu P^\nu{}_\lambda\xi^\lambda + N\Delta^{\nu\lambda}\tilde{I}_\lambda\tilde{I}_\nu - \frac{1}{2}P^\mu_\kappa\xi^\kappa\tilde{I}_{\mu\nu}P^\nu{}_\lambda\xi^\lambda + \frac{\sqrt{N}}{2}P^\mu{}_\kappa\xi^\kappa\tilde{I}_{\mu\nu}\Delta^{\nu\lambda}\tilde{I}_\lambda + \\ &\quad + \frac{\sqrt{N}}{2}\Delta^{\mu\kappa}\tilde{I}_\kappa\tilde{I}_{\mu\nu}P^\nu{}_\lambda\xi^\lambda - \frac{N}{2}\Delta^{\mu\kappa}\tilde{I}_\kappa\tilde{I}_{\mu\nu}\Delta^{\nu\lambda}\tilde{I}_\lambda \\ &= \frac{N}{2}\tilde{I}_\nu\Delta^{\nu\lambda}\tilde{I}_\lambda - \frac{1}{2}\xi^\kappa\delta_{\kappa\lambda}\xi^\lambda \end{aligned} \tag{21}$$

where we have used Equation 17 as well as the fact that $\Delta^{\mu\nu} = \Delta^{\nu\mu}$ and that $\Delta^{\mu\kappa}\tilde{I}_{\kappa\nu} = \delta^\mu{}_\nu$ by definition.

Therefore, putting Equation 19 and Equation 21 together, Equation 13 becomes

$$Q = \frac{\exp\left[\frac{N}{2}\tilde{I}_\mu\Delta^{\mu\nu}\tilde{I}_\nu\right]}{\sqrt{\det \tilde{I}_{\mu\nu}}} \int_\Xi \mathrm{d}^d\xi \exp\left[-\frac{1}{2}\xi_\mu\delta^{\mu\nu}\xi_\nu\right] \tag{22}$$

The problem is reduced to a (truncated) spherical gaussian integral, where the domain of integration Ξ will depend on the original domain Θ but also on $\tilde{I}_\mu$, $\tilde{I}_{\mu\nu}$ and $\hat{\vartheta}$. To complete the calculation, we now need to make this dependence explicit.

bioRxiv preprint doi: https://doi.org/10.1101/2023.01.10.523479; this version posted February 8, 2023. The copyright holder for this preprint (which was not certified by peer review) is the author/funder, who has granted bioRxiv a license to display the preprint in perpetuity. It is made available under aCC-BY 4.0 International license.

A.1.4 Determining the domain of integration

We start by combining Equation 7 and Equation 20 to yield:

$$\vartheta^\mu = \frac{1}{\sqrt{N}} P^\mu{}_\nu \xi^\nu - \Delta^{\mu\nu} \tilde{I}_\nu + \hat{\vartheta}^\mu \tag{23}$$

By substituting Equation 23 into Equation 5 we get

$$D_\mu \left(\frac{P^\mu{}_\nu \xi^\nu}{\sqrt{N}} - \Delta^{\mu\nu} \tilde{I}_\nu + \hat{\vartheta}^\mu \right) + d \geq 0$$

which we can rewrite as

$$\tilde{D}_\mu \xi^\mu + \tilde{d} \geq 0 \tag{24}$$

with

$$\tilde{D}_\mu := \frac{1}{\sqrt{N}} D_\nu P^\nu{}_\mu \tag{25}$$

and

$$\begin{aligned} \tilde{d} &:= d + D_\mu \hat{\vartheta}^\mu - D_\mu \Delta^{\mu\nu} \tilde{I}_\nu \\ &= d + D_\mu \hat{\vartheta}^\mu - \langle D_\mu, \tilde{I}_\mu \rangle_\Delta \end{aligned} \tag{26}$$

where by $\langle \cdot, \cdot \rangle_\Delta$ we mean the inner product in the inverse observed Fisher information metric. Now, note that whenever $\tilde{I}_\mu$ is not zero, it will be parallel to D_μ. Indeed, by construction of the maximum-likelihood point $\hat{\vartheta}$, the gradient of the log likelihood can only be orthogonal to the boundary at $\hat{\vartheta}$, and pointing towards the outside of the domain. Therefore $\tilde{I}_\mu$, which is defined as minus the gradient, will point inward. At the same time, D_μ will also always point toward the interior of the domain because of the form of the constraint we have chosen in Equation 5. Because by assumption $||D_\mu|| = 1$, we have that

$$\tilde{I}_\mu = ||\tilde{I}_\nu|| D_\mu$$

and

$$\langle D_\mu, \tilde{I}_\mu \rangle_\Delta = ||D_\nu||_\Delta \cdot ||\tilde{I}_\nu||_\Delta$$

so that

$$\tilde{d} = d + D_\mu \hat{\vartheta}^\mu - ||D_\mu||_\Delta \cdot ||\tilde{I}_\mu||_\Delta \tag{27}$$

Now, the signed distance of the boundary to the origin in ξ-space is

$$l = -\frac{\tilde{d}}{||\tilde{D}_\mu||}$$

where the sign is taken such that l is negative when the origin is included in the integration domain. But noting that

$$K^\mu{}_\kappa \Delta^{\kappa\lambda} K^\nu{}_\lambda = \delta^{\mu\nu} \quad \Rightarrow \quad \Delta^{\mu\nu} = P^\mu{}_\kappa \delta^{\kappa\lambda} P^\nu{}_\lambda$$

we have

$$\begin{aligned} ||\tilde{D}_\mu|| = \sqrt{\tilde{D}_\mu \delta^{\mu\nu} \tilde{D}_\nu} &= \sqrt{\frac{1}{N} D_\kappa \left(P^\kappa{}_\mu \delta^{\mu\nu} P^\lambda{}_\nu \right) D_\lambda} \\ &= \sqrt{\frac{1}{N} D_\kappa \Delta^{\kappa\lambda} D_\lambda} = \frac{||D_\mu||_\Delta}{\sqrt{N}} \end{aligned}$$

and therefore

$$l = -\sqrt{N} \frac{\tilde{d}}{||D_\mu||} \tag{28}$$

bioRxiv preprint doi: https://doi.org/10.1101/2023.01.10.523479; this version posted February 8, 2023. The copyright holder for this preprint (which was not certified by peer review) is the author/funder, who has granted bioRxiv a license to display the preprint in perpetuity. It is made available under aCC-BY 4.0 International license.

Finally, by plugging Equation 27 into Equation 28 we obtain

$$l = -\sqrt{N}\left[\frac{d + D_\mu \hat{\vartheta}^\mu}{\|D_\mu\|_\Delta} - \|\tilde{I}_\mu\|_\Delta\right] \\ =: \sqrt{2}\,(s - m) \tag{29}$$

where m and s are defined for convenience like so:

$$m := \sqrt{\frac{N}{2}}\frac{d + D_\mu \hat{\vartheta}^\mu}{\|D_\mu\|_\Delta} \quad (\geq 0) \tag{30}$$

$$s := \sqrt{\frac{N}{2}}\|\tilde{I}_\mu\|_\Delta \quad (\geq 0) \tag{31}$$

We note that m is a rescaled version of the margin defined by the constraint on the parameters (and therefore is never negative by assumption), and s is a rescaled version of the norm of the gradient of the log likelihood in the inverse observed Fisher metric (and therefore is nonnegative by construction).

A.1.5 Computing the penalty

We can now perform a final change of variables in the integral in Equation 22. We rotate our coordinates to align them to the boundary, so that

$$\tilde{D}_\mu = (\|\tilde{D}_\mu\|, 0, 0, \ldots, 0)$$

Note that we can always do this as our integrand is invariant under rotation. In this coordinate system, Equation 22 factorizes:

$$\begin{aligned} Q &= \frac{\exp\left[\frac{N}{2}\tilde{I}_\mu \Delta^{\mu\nu}\tilde{I}_\nu\right]}{\sqrt{\det \tilde{I}_{\mu\nu}}} \int_{\mathbb{R}^{d-1}} \mathrm{d}^{d-1}\xi \exp\left[-\frac{\xi_\mu \delta^{\mu\nu}\xi_\nu}{2}\right] \int_l^\infty \mathrm{d}\zeta \exp\left[-\frac{\zeta^2}{2}\right] \\ &= \sqrt{\frac{(2\pi)^d}{\det \tilde{I}_{\mu\nu}}} \exp\left[\frac{N}{2}\|\tilde{I}\|_\Delta^2\right]\frac{1}{\sqrt{\pi}} \int_l^\infty \frac{\mathrm{d}\zeta}{\sqrt{2}} \exp\left[-\frac{\zeta^2}{2}\right] \\ &= \sqrt{\frac{(2\pi)^d}{\det \tilde{I}_{\mu\nu}}} \exp\left(s^2\right)\frac{1}{\sqrt{\pi}} \int_{l/\sqrt{2}}^\infty \mathrm{d}\zeta \exp\left[-\zeta^2\right] \\ &= \sqrt{\frac{(2\pi)^d}{\det \tilde{I}_{\mu\nu}}} \exp\left(s^2\right)\frac{\operatorname{erfc}(s-m)}{2} \end{aligned} \tag{32}$$

where $\operatorname{erfc}(\cdot)$ is the complementary error function (*47*, section 7.1.2).

Finally, plugging Equation 32 into Equation 10 and taking the log, we obtain the extended FIA:

$$-\ln \mathbb{P}(\mathcal{M}_1|E) \simeq \ln \mathbb{P}(E|\hat{\vartheta}) + \frac{d}{2}\ln\frac{N}{2\pi} + \ln\int_\Theta \mathrm{d}^d\vartheta\sqrt{\det g} + \frac{1}{2}\ln\left[\frac{\det \tilde{I}_{\mu\nu}}{\det g_{\mu\nu}}\right] + B \tag{33}$$

where

$$B := \ln(2) - \ln\left[\exp\left(s^2\right)\operatorname{erfc}(s-m)\right] \tag{34}$$

can be interpreted as a penalty arising from the presence of the boundary in parameter space.

A.1.6 Interpreting the penalty

We now take a closer look at Equation 34. One key observation is that, by construction, at most one of m and s is ever nonzero. This is because in the interior of the manifold, $m > 0$ by definition, but $s = 0$ because the gradient of the likelihood is zero at $\hat{\vartheta}$; and on the boundary, $m = 0$ by definition, and s can be either zero or positive.

bioRxiv preprint doi: https://doi.org/10.1101/2023.01.10.523479; this version posted February 8, 2023. The copyright holder for this preprint (which was not certified by peer review) is the author/funder, who has granted bioRxiv a license to display the preprint in perpetuity. It is made available under aCC-BY 4.0 International license.

Interior of the manifold When $\hat{\vartheta}$ is in the interior of the parameter space Θ, then $\tilde{I}_\mu = 0 \Rightarrow s = 0$ and Equation 34 simplifies to

$$B = \ln(2) - \ln(\text{erfc}(-m)) \tag{35}$$

but since N is large we have $m \gg 0$, $\text{erfc}(-m) \to 2$ and $B \to 0$, so our result passes the first sanity check: we recover the expression in (*11*).

Boundary of the manifold When $\hat{\vartheta}$ is on the boundary of Θ, $m = 0$ and $s \geq 0$. Equation 34 becomes

$$B = \ln(2) - \ln\left[\exp\left(s^2\right)\text{erfc}(s)\right] = \ln(2) - \ln(w(is)) \tag{36}$$

where w is the Feddeeva function (*47*, p. 7.1.3):

$$w(z) = e^{-z^2}\,\text{erfc}(-iz)$$

This function is tabulated and can be computed efficiently. However, it is interesting to analyze its limiting behavior, as follows.

As a consistency check, when s is small we have at fixed N, to first order:

$$\begin{aligned} B &\simeq \ln(2) - \ln\left(1 - \frac{2s}{\sqrt{\pi}}\right) \\ &\simeq \ln(2) + \frac{2s}{\sqrt{\pi}} = \ln(2) + \sqrt{\frac{2N}{\pi}}\|\tilde{I}_\mu\|_\Delta \end{aligned} \tag{37}$$

and $B = \ln(2)$ when $\tilde{I}_\mu = 0$, as expected.

However, the real case of interest is the behavior of the penalty when N is assumed to be large, which is consistent with the fact that we derived Equation 32 as an asymptotic expansion of Equation 11. In this case, using the asymptotic expansion for the Feddeeva function (*47*, section 7.1.23):

$$\exp\left[s^2\right]\text{erfc}(s) \sim \frac{1}{s\sqrt{\pi}}\left[1 + \sum_{m=1}^{\infty}(-1)^m \frac{1\cdot 3\cdots(2m-1)}{(2s^2)^m}\right]$$

To leading order, we obtain

$$\begin{aligned} B &\simeq \ln(2) + \ln\left(s\sqrt{\pi}\right) \\ &= \ln(2) + \ln\left(\sqrt{\frac{N\pi}{2}}\|\tilde{I}_\mu\|_\Delta\right) \end{aligned}$$

which we can rewrite as

$$\boxed{B \simeq \frac{1}{2}\ln\frac{N}{2\pi} + \ln\left[2\pi\|\tilde{I}_\mu\|_\Delta\right]} \tag{38}$$

We can summarize the above by saying that a new penalty term of order $\ln N$ arose due to the presence of the boundary. Interestingly, comparing Equation 38 with Equation 33 we see that the first term in Equation 38 is analogous to counting an extra parameter dimension in the original Fisher Information Approximation.

A.2 Behavioral experiments with human subjects

The behavioral task required subjects to view a screen showing two curves (one on the upper half, the other on the lower half of the screen) and 10 dots and decide, based on different instructions (see below for details), which curve was the more likely source of the observed dots. There were four task types that differed in terms of the shapes of the curves, corresponding to the different terms of the FIA (see Figure 1 in the main text and Figure S1): *dimensionality*, *boundary*, *volume*, and *robustness*. In each case, the curves represent two parametric statistical models of the form:

$$p(x|t) = \frac{1}{\sqrt{2\pi\sigma^2}}\exp\left[-\frac{\|x-\mu(t)\|^2}{2\sigma^2}\right] \tag{39}$$

bioRxiv preprint doi: https://doi.org/10.1101/2023.01.10.523479; this version posted February 8, 2023. The copyright holder for this preprint (which was not certified by peer review) is the author/funder, who has granted bioRxiv a license to display the preprint in perpetuity. It is made available under aCC-BY 4.0 International license.

where x is a location on the 2D plane visualized on the screen, and $\mu(t)$, $t \in [0,1]$ is a parametrization of the curve. In other words, the curves represent Gaussians of unit isotropic variance whose mean μ can be located at any point along them. The dots shown to the subjects were sampled iid from one of the two models, selected at random with uniform probability. The location of the true mean of the Gaussian generating the dots (i.e., the value of t in the expression above) was randomly sampled from Jeffrey's prior for the selected model. All dots shown within a trial come from the same distribution (same model and same true mean). In the "generative" version of the task, the subjects had to report which curve (model) the dots are more likely to come from. In the "maximum-likelihood" version, the subjects had to report which curve was closest to the empirical centroid of the dot cloud. In both versions of the task, they pressed the "up" or "down" keys on their keyboard to select the curve in the upper or lower part of the screen, respectively.

Each model pairing was designed to emphasize a different term of the FIA. In the dimensionality variant, models have different dimensionality ($d = 0$ for the point and $d = 1$ for the line). In the boundary variant, both models have the same dimensionality and volume and are both flat so that their robustness terms are always identically zero. However, they are oriented such that, for ambiguous data falling around the midpoint between the two models, the influence of the boundary of the vertical model is stronger than that of the horizontal model. In the volume variant, models have the same dimensionality but different volume (length). In the robustness variant, models have the same dimensionality and volume, but their curvature is such that one of them bends away from the region of data space that is more likely to contain ambiguous stimuli, whereas the other bends around it (and therefore the robustness term for these models has opposite sign for data points that fall in that region).

A single run of the task consisted of a brief tutorial followed by 500 trials, divided in 5 blocks of 100 trials each. For each trial, the chosen curve pairing was presented, randomly flipped vertically to dissociate a fixed preference for one of the two models from a fixed preference for reporting "up" or "down". At the end of each block, the subject received feedback on their overall performance during that block. Subjects received a fixed compensation for taking part in the experiment.

We ran both experiments (generative and maximum-likelihood) on the online platform Pavlovia (`pavlovia.org`). For each task type, we collected data from at least 50 subjects who passed a pre-established performance threshold (60% correct for the robustness task variant and 70% correct for the other variants; these thresholds were chosen based on pilot data, and were fixed at preregistration (*24–26*)). We discarded the data collected from all other subjects. For the generative task, the final dataset included 52 subjects for the robustness task variant and 50 subjects for each of the other task variants. For the maximum-likelihood task, the final dataset included 51 subjects for the dimensionality task variant and 50 subjects for each of the others.

A.3 Detailed model definitions and computation of FIA terms

In this section, we report the detailed mathematical form of the models we used for the psychophysics experiment. Each model is defined by specifying the form of the function μ in Equation 39. Given this function, we then derive the analytical solution to the maximum-likelihood problem for any value of $X = \{x_i\}_{i=1}^N$, and finally the expressions for the likelihood (L), dimensionality (D), boundary (B), volume (V) and robustness (R) terms in the FIA for the model pairings we use in the experiment.

We also show that the (expected) Fisher information is constant for all models considered:

$$g(t) \equiv \frac{T^2}{\sigma^2} \tag{40}$$

so that Jeffrey's prior is simply the uniform probability distribution over the $[0,1]$ interval:

$$w(t) = \mathbb{1}_{[0,1]}(t) \tag{41}$$

A.3.1 Fisher information and robustness term for curved exponential families

In the following, we compute the observed Fisher information for each of our models. To do so, it is convenient to have a general expression for the Hessian of the log likelihood and for the observed and expected Fisher information for curved exponential families.

bioRxiv preprint doi: https://doi.org/10.1101/2023.01.10.523479; this version posted February 8, 2023. The copyright holder for this preprint (which was not certified by peer review) is the author/funder, who has granted bioRxiv a license to display the preprint in perpetuity. It is made available under aCC-BY 4.0 International license.

The general form of a curved exponential family is:

$$p(x|u) = \exp\left[C(x) + \vartheta^i(u)F_i(x) - \psi(\vartheta(u))\right] \tag{42}$$

where $\vartheta(u) : \mathbb{R}^d \to \mathbb{R}^k$, $k \geq d$, is a smooth parametrization. The Hessian of the log-likelihood is:

$$\begin{aligned}
\partial_a \partial_b \log p(x|u) &= F_i(x)\partial_a\partial_b\vartheta^i(u) - \partial_a\partial_b\psi(\vartheta(u)) \\
&= F_i(x)\frac{\partial^2\vartheta^i}{\partial u^a \partial u^b} - \frac{\partial}{\partial u^a}\left(\frac{\partial\psi}{\partial\vartheta^i}\frac{\partial\vartheta^i}{\partial u^b}\right) \\
&= F_i(x)\frac{\partial^2\vartheta^i}{\partial u^a \partial u^b} - \frac{\partial\vartheta^j}{\partial u^a}\frac{\partial\psi}{\partial\vartheta^j\partial\vartheta^i}\frac{\partial\vartheta^i}{\partial u^b} - \frac{\partial\psi}{\partial\vartheta^i}\frac{\partial^2\vartheta^i}{\partial u^a\partial u^b} \\
&= \frac{\partial^2\vartheta^i}{\partial u^a\partial u^b}\left[F_i(x) - \mathbb{E}_u[F_i]\right] - \frac{\partial\vartheta^j}{\partial u^a} g_{ji} \frac{\partial\vartheta^i}{\partial u^b}
\end{aligned} \tag{43}$$

where we note that $g_{ij} = -\mathrm{Cov}_u[F]_{ji}$ (remember that by g_{ij} we indicate the Fisher information of the ambient family). Therefore, the (expected) Fisher information is:

$$g_{ab} = \mathbb{E}_u\left[-\partial_a\partial_b \log p(x_i|u)\right] = \frac{\partial\vartheta^j}{\partial u^a} g_{ji} \frac{\partial\vartheta^i}{\partial u^b} \tag{44}$$

and the oberved Fisher information is:

$$\begin{aligned}
h_{ab} &= -\frac{1}{N}\sum_{i=1}^{N}\partial_a\partial_b \log p(x_i|u) \\
&= g_{ab} + \frac{\partial^2\vartheta^i}{\partial u^a\partial u^b}\left[\mathbb{E}_u[F_i] - \frac{1}{N}\sum_{n=1}^{N}F_i(x_n)\right]
\end{aligned} \tag{45}$$

As a corollary, we note that $h_{ab} = g_{ab}$ whenever $\vartheta(\cdot)$ is an affine transformation, that is when

$$\vartheta^i(u) = A^i_a u^a + B^i \tag{46}$$

For some constant A^i_b and B^i. In this case (which corresponds to autoparallel submanifolds in the exponential connection, (*48*, Theorem 1.1)), the robustness term in the FIA is identically zero:

$$\vartheta^i(u) = A^i_a u^a + B^i \Rightarrow R(X;u) \equiv 0 \tag{47}$$

A.3.2 General properties of curved 2D Gaussian models

Our models of interest, defined through Equation 39, are a special case of curved exponential families. They are all submanifolds of the same, larger model — the 2-dimensional exponential family of 2D Gaussian distributions with known isotropic covariance and unknown center. We call this larger family the *ambient family* $\mathcal{S} \supset \mathcal{M}$, composed by all probability distributions whose density is of the form:

$$p(x|\mu) = \frac{1}{2\pi\sigma^2}\exp\left[-\frac{||x-\mu||^2}{2\sigma^2}\right] \tag{48}$$

We can reduce Equation 39 to the notation of Equation 42 by noting that

$$\begin{aligned}
\ln p(x|t) &= -\frac{||x-\mu||^2}{2\sigma^2} - \ln\left(2\pi\sigma^2\right) \\
&= -\frac{1}{2}||x||^2_{g_{ij}} + \mu^i(t)g_{ij}x^j - \frac{1}{2}\left[||\mu(t)||^2_{g_{ij}} + \ln\left((2\pi)^2 \det g_{ij}\right)\right]
\end{aligned} \tag{49}$$

where we indicate by g_{ij} the Fisher information of the ambient family $\mathcal{S}$:

$$g_{ij} = \begin{bmatrix} 1/\sigma^2 & 0 \\ 0 & 1/\sigma^2 \end{bmatrix} \tag{50}$$

bioRxiv preprint doi: https://doi.org/10.1101/2023.01.10.523479; this version posted February 8, 2023. The copyright holder for this preprint (which was not certified by peer review) is the author/funder, who has granted bioRxiv a license to display the preprint in perpetuity. It is made available under aCC-BY 4.0 International license.

By comparing Equation 49 with Equation 42 we see that

$$C(x) = -\frac{1}{2}\|x\|^2_{g_{ij}} \tag{51}$$

$$F^i(x) = x^i \qquad (\text{and } F_i(x) = g_{ij}x^j = \frac{x^i}{\sigma^2}) \tag{52}$$

$$\psi(t) = \frac{1}{2}\left[\|\mu(t)\|^2_{g_{ij}} + \ln\left((2\pi)^2 \det g_{ij}\right)\right] \tag{53}$$

and that $\mu(t)$ plays the role that $\vartheta(u)$ played in Equation 42.

We can now compute the expected and observed Fisher information for our models by specializing Equation 44 and Equation 45:

$$g(t) = \dot{\mu}^i(t) g_{ij} \dot{\mu}^j(t) \tag{54}$$

$$h(t) = g(t) + \ddot{\mu}^i(t) g_{ij}\left[\mu^j(t) - \bar{x}\right] \tag{55}$$

Where $\bar{x}$ is the empirical centroid of the dataset X,

$$\bar{x} = \bar{x}(X) := \frac{1}{N}\sum_{i=1}^{N} x_i \tag{56}$$

and g and h have no indices, because they are scalar functions of t.

We note then that $g(t)$ is simply the squared Euclidean norm of the vector $\dot{\mu}(t)$ divided by σ^2. In other words, the geometry of $\mathcal{M}$ coincides, up to scaling by σ^2, with the Euclidean geometry of the plane curve $\mu(t)$. This very convenient fact is a consequence of the particularly simple noise model we have assumed (Gaussian with known isotropic covariance).

Model volume The volume of a model described by $\mu(\cdot)$ is

$$\int_0^1 \mathrm{d}t\sqrt{g(t)} = \int_0^1 \mathrm{d}t\sqrt{\dot{\mu}^i(t) g_{ij} \dot{\mu}^j(t)} = \frac{1}{\sigma}\int_0^1 \mathrm{d}t \|\dot{\mu}(t)\| \tag{57}$$

In other words, it is simply the length of the curve $\mu(\cdot)$ measured in units of σ.

Likelihood gradient and maximum-likelihood point In the following, we will indicate the log-likelihood function for a model by

$$l = l(x;t) = \ln p(x|t) \tag{58}$$

In order to find the maximum-likelihood point for our models, it is convenient to write a general expression for the score function (the derivative of the log likelihood with respect to the parameter). We start by noting that

$$\frac{\partial}{\partial t}\ln p(X|t) = -\sum_i \frac{\partial}{\partial t}\frac{\|x_i - \mu(t)\|^2}{2\sigma^2} = \frac{N}{2\sigma^2}\left[\frac{2}{N}\sum_n x_n - \mu(t)\right]\cdot \dot{\mu}(t)$$
$$= N\frac{\partial}{\partial t}\ln p(\bar{x}|t) = N\frac{\partial l(\bar{x};t)}{\partial t}$$

Therefore, to find the maximum likelihood point $\hat{t}$ for a certain X we can simply solve the corresponding one-sample ($N = 1$) case for the centroid $\bar{x}$. We can also write the rescaled likelihood gradient (which appears in the FIA as I_μ) as

$$-\frac{1}{N}\frac{\partial l}{\partial t}(X;t) = -\frac{1}{N}\frac{\partial}{\partial t}\ln p(X|\mu(t)) = \dot{\mu}^i(t) g_{ij}\left[\mu^j(t) - \bar{x}^j\right] \tag{59}$$

If we interpret $\dot{\mu}(t)$ as the tangent vector to μ in t, we see that away from model boundaries this equation expresses the familiar condition that the maximum-likelihood point (where $\partial l/\partial t = 0$) is the (Euclidean) orthogonal projection of $\bar{x}$ onto the model manifold. Again, this convenient property is a consequence of assuming isotropic Gaussian noise.

bioRxiv preprint doi: https://doi.org/10.1101/2023.01.10.523479; this version posted February 8, 2023. The copyright holder for this preprint (which was not certified by peer review) is the author/funder, who has granted bioRxiv a license to display the preprint in perpetuity. It is made available under aCC-BY 4.0 International license.

A.3.3 Horizontal model

This model, used in the dimensionality, boundary, and volume task variants, is defined as

$$\mu(t) = \begin{bmatrix} T\left(t - \frac{1}{2}\right) \\ \tau \end{bmatrix} \tag{60}$$

It is immediately evident that this model has volume (length) T/σ. The "base" model corresponds to $T = 1$, $\tau = 0$, and the model type called "horizontal" is defined with $T = 3$, $\tau = 1$.

Because

$$\dot{\mu}(t) = T \begin{bmatrix} 1 \\ 0 \end{bmatrix} \tag{61}$$

and following Equation 54 and Equation 55, the observed and expected Fisher information coincide and are given by

$$g = h = \frac{T^2}{\sigma^2} \tag{62}$$

Given a centroid $X = [X^1, X^2]^T$, the rescaled likelihood gradient is (from Equation 59)

$$\frac{1}{N}\frac{\partial l}{\partial t}(X;t) = \frac{T^2}{\sigma^2}\left[\frac{\bar{x}^1}{T} - \left(t - \frac{1}{2}\right)\right] \tag{63}$$

and the maximum-likelihood point $\hat{t}$ is

$$\hat{t}(X) = \begin{cases} 0 & \text{if } \bar{x}^1 < -T/2 \\ \frac{1}{2} + \frac{\bar{x}^1}{T} & \text{if } -T/2 < \bar{x}^1 < T/2 \\ 1 & \text{if } \bar{x}^1 > T/2 \end{cases} \tag{64}$$

All the FIA terms can be computed in closed form from these expressions:

$$L(X) = -\frac{N}{2\sigma^2}\left[(\bar{x}^1 - T(\hat{t}(X) - 1/2))^2 + (\bar{x}^2 - \tau)^2\right] - \frac{N}{2}\ln\left(2\pi\sigma^2\right) \tag{65}$$

$$D = \frac{1}{2}\ln\frac{N}{2\pi} \tag{66}$$

$$B = \frac{1}{2}\ln\frac{N}{2\pi} + \ln\left[2\pi\frac{T}{\sigma}\left|\frac{\bar{x}^1}{T} - \left(\hat{t}(X) - \frac{1}{2}\right)\right|\right] \quad \left(\text{if } |\bar{x}^1| > \frac{T}{2}\right) \tag{67}$$

$$V = \ln\frac{T}{\sigma} \tag{68}$$

$$R = 0 \tag{69}$$

A.3.4 Vertical model

This model, used the boundary task variant, is just a rotated and translated version of the horizontal model. It is defined as

$$\mu(t) = \begin{bmatrix} 0 \\ \tau + Tt \end{bmatrix} \tag{70}$$

where we keep T and τ as arbitrary parameters for notational clarity, although in practice they are both fixed to 1 in our study. From the definition, it follows that

$$\dot{\mu}(t) = T \begin{bmatrix} 0 \\ 1 \end{bmatrix} \tag{71}$$

$$g = h = \frac{T^2}{\sigma^2} \tag{72}$$

$$\frac{1}{N}\frac{\partial l}{\partial t}(X;t) = \frac{T^2}{\sigma^2}\left[\frac{X^2 - \tau}{T} - t\right] \tag{73}$$

bioRxiv preprint doi: https://doi.org/10.1101/2023.01.10.523479; this version posted February 8, 2023. The copyright holder for this preprint (which was not certified by peer review) is the author/funder, who has granted bioRxiv a license to display the preprint in perpetuity. It is made available under aCC-BY 4.0 International license.

and

$$\hat{t}(X) = \begin{cases} 0 & \text{if } \bar{x}^2 < \tau \\ \frac{\bar{x}^2-\tau}{T} & \text{if } \tau < \bar{x}^2 < \tau + T \\ 1 & \text{if } \bar{x}^2 > \tau + T \end{cases} \tag{74}$$

so that the FIA terms can be written as

$$L(X) = -\frac{N}{2\sigma^2}\left[(\bar{x}^1)^2 + (\bar{x}^2 - \tau - T\hat{t}(X))^2\right] - \frac{N}{2}\ln\left(2\pi\sigma^2\right) \tag{75}$$

$$D = \frac{1}{2}\ln\frac{N}{2\pi} \tag{76}$$

$$B = \frac{1}{2}\ln\frac{N}{2\pi} + \ln\left[2\pi\frac{T}{\sigma}\left|\frac{\bar{x}^2-\tau}{T} - \hat{t}(X)\right|\right] \quad \left(\text{if } \bar{x}^2 < \tau \vee \bar{x}^2 > \tau + T\right) \tag{77}$$

$$V = \ln\frac{T}{\sigma} \tag{78}$$

$$R = 0 \tag{79}$$

A.3.5 Circular-arc model

This model, used in the robustness task variant, is constituted by an arc of a circle, and is defined as

$$\mu(t) = \begin{bmatrix} \frac{T}{\gamma}\sin(\alpha) \\ \tau + \frac{T}{\gamma}(1-\cos(\alpha)) \end{bmatrix} \tag{80}$$

where

$$\alpha = \gamma\left(t - \frac{1}{2}\right) \tag{81}$$

and γ is a positive constant. Concretely, in the experiments we fixed $\gamma = (3/5)\pi$, and T to the value determined below for the rounded model type (Equation 99). We note that the radius of the circle is $r = T/\gamma$, and the y-coordinate of the center is $\tau + r$. The tangent vector $\dot{\mu}$ and the acceleration vector $\ddot{\mu}$ in t are

$$\dot{\mu}(t) = T\begin{bmatrix} \cos(\alpha) \\ \sin(\alpha) \end{bmatrix} \tag{82}$$

$$\ddot{\mu}(t) = T\gamma\begin{bmatrix} -\sin(\alpha) \\ \cos(\alpha) \end{bmatrix} \tag{83}$$

so that, by substitution in Equation 54,

$$g = \dot{\mu}^i g_{ij}\dot{\mu}^j = \frac{T^2}{\sigma^2}(\cos^2(\alpha) + \sin^2(\alpha)) = \frac{T^2}{\sigma^2} \tag{84}$$

and by substitution in Equation 55

$$\begin{aligned} h &= g + \frac{T^2}{\sigma^2}\left[-\sin^2(\alpha) + \frac{\gamma\bar{x}^1}{T}\sin(\alpha) + \frac{\gamma\tau}{T}\cos(\alpha) + \cos(\alpha) - \cos^2(\alpha) - \frac{\gamma\bar{x}^2}{T}\cos(\alpha)\right] \\ &= g\left[\frac{\gamma\bar{x}^1}{T}\sin(\alpha) + \frac{\gamma\tau}{T}\cos(\alpha) + \cos(\alpha) - \frac{\gamma\bar{x}^2}{T}\cos(\alpha)\right] \\ &= \frac{g}{r}\left[\sin(\alpha)\bar{x}^1 + \cos(\alpha)\left(\tau + r - \bar{x}^2\right)\right] \end{aligned} \tag{85}$$

The rescaled likelihood gradient is (from Equation 59)

$$\begin{aligned} -\frac{1}{N}\frac{\partial}{\partial t}\ln(p(X|t)) &= \frac{T^2}{\gamma\sigma^2}\Big[\cos(\alpha)\sin(\alpha) - \frac{\gamma\bar{x}^1}{T}\cos(\alpha) \\ &\quad + \frac{\gamma\tau}{T}\sin(\alpha) + \sin(\alpha) - \sin(\alpha)\cos(\alpha) - \frac{\gamma\bar{x}^2}{T}\sin(\alpha)\Big] \\ &= \frac{T}{\sigma^2}\left[-\bar{x}^1\cos(\alpha) + \tau\sin(\alpha) + r\sin(\alpha) - \bar{x}^2\sin(\alpha)\right] \\ &= \frac{T}{\sigma^2}\left[-\cos(\alpha)\bar{x}^1 + \sin(\alpha)\left(\tau + r - \bar{x}^2\right)\right] \end{aligned} \tag{86}$$

bioRxiv preprint doi: https://doi.org/10.1101/2023.01.10.523479; this version posted February 8, 2023. The copyright holder for this preprint (which was not certified by peer review) is the author/funder, who has granted bioRxiv a license to display the preprint in perpetuity. It is made available under aCC-BY 4.0 International license.

(note that h can also be obtained by differentiating this last expression).

To compute the FIA, we need the maximum-likelihood projection. As for the other models, this projection is defined piecewise due to the presence of model boundaries. To properly partition the plane, we need to define first the equation for the line intersecting the model perpendicularly at t:

$$\rho(x;t) = \tau + r - \cot(\alpha(t))x \tag{87}$$

With this definition, the maximum-likelihood point is

$$\hat{t}(X) = \begin{cases} 0 & \text{if } \bar{x}^1 < 0 \wedge \bar{x}^2 > \rho(\bar{x}^1;0) \\ 1 & \text{if } \bar{x}^1 > 0 \wedge \bar{x}^2 > \rho(\bar{x}^1;1) \\ \frac{1}{2} + \frac{1}{\gamma}\arctan\frac{\bar{x}^1}{\tau+r-\bar{x}^2} & \text{otherwise} \end{cases} \tag{88}$$

and therefore the FIA terms are:

$$L(X) = -\frac{N}{2\sigma^2}\|\bar{x} - \mu(\hat{t}(X))\|^2 - \frac{N}{2}\ln\left(2\pi\sigma^2\right) \tag{89}$$

$$D = \frac{1}{2}\ln\frac{N}{2\pi} \tag{90}$$

$$B = \frac{1}{2}\ln\frac{N}{2\pi} + \ln\left[2\pi\sqrt{\frac{r}{\sigma^2}\frac{\left[-\cos\left(\alpha(\hat{t})\right)\bar{x}^1 + \sin\left(\alpha(\hat{t})\right)(\tau+r-\bar{x}^2)\right]^2}{\sin\left(\alpha(\hat{t})\right)\bar{x}^1 + \cos\left(\alpha(\hat{t})\right)(\tau+r-\bar{x}^2)}}\right] \tag{91}$$

$$V = \ln\frac{T}{\sigma} \tag{92}$$

$$R = \sin\left(\alpha(\hat{t})\right)\frac{\bar{x}^1}{r} + \cos\left(\alpha(\hat{t})\right)\frac{\tau+r-\bar{x}^2}{r} \tag{93}$$

where the value given for B is relevant only when $\hat{t}$ is either 0 or 1. Note that, due to the shape of the model and the presence of the boundary, there are regions of the data space such that the log-likelihood function at the maximum likelihood point will not be concave. These regions represent a complete breakdown of the FIA, but they are not a problem in practice because the approximation holds in the region of data space that is relevant for the experiments (see Figure S1).

A.3.6 Rounded model

This model, also used in the robustness task variant, is a circular arc (like the "circular" model described above) with two straight arms attached on either side. The ratio of the length of the circular section of the model over its total length is defined as a parameter f. The model definition is

$$\mu(t) = \begin{cases} \begin{bmatrix} -T\left[\frac{f}{\gamma}\sin(\gamma/2) - \left(t - \frac{(1-f)}{2}\right)\cos(\gamma/2)\right] \\ \tau + T\left[\frac{f}{\gamma}(1-\cos(\gamma/2)) - \left(t - \frac{1-f}{2}\right)\sin(\gamma/2)\right] \end{bmatrix} & \text{if } t < \frac{1-f}{2} \\ \mu_c\left(\frac{t-(1-f)/2}{f};\tau,\gamma,fT\right) & \text{if } \frac{1-f}{2} \le t \le \frac{1+f}{2} \\ \begin{bmatrix} T\left[\frac{f}{\gamma}\sin(\gamma/2) + \left(t - \frac{(1+f)}{2}\right)\cos(\gamma/2)\right] \\ \tau + T\left[\frac{f}{\gamma}(1-\cos(\gamma/2)) + \left(t - \frac{1+f}{2}\right)\sin(\gamma/2)\right] \end{bmatrix} & \text{if } t > \frac{1+f}{2} \end{cases} \tag{94}$$

where μ_c is the μ mapping defined for the circular model, Equation 80.

For the experiment, the values of the parameters were chosen to guarantee that the circular section of this model would have the same center as a circular model (described above) with $\gamma = (3/5)\pi$ and $\tau = 0$,

bioRxiv preprint doi: https://doi.org/10.1101/2023.01.10.523479; this version posted February 8, 2023. The copyright holder for this preprint (which was not certified by peer review) is the author/funder, who has granted bioRxiv a license to display the preprint in perpetuity. It is made available under aCC-BY 4.0 International license.

and that a relatively large fraction of the two models is in close proximity. The values are

$$f = 1/3 \tag{95}$$
$$\gamma = (3/5)\pi \tag{96}$$
$$\tau = 3/5 \tag{97}$$
$$T = \frac{\tau\gamma}{1-f} \tag{98}$$

Closed-form expressions for all FIA terms can be derived for this model by a straightforward, if somewhat laborious, extension of those presented above for the circular-arc model. We do not report them here in the interest of brevity.

A.3.7 Point model

This model, used in the dimensionality task variant, has no associated latent parameters (it is zero-dimensional). To cast it in the same language as the others, we can define it as

$$\mu(t) = \mu = \begin{bmatrix} 0 \\ \tau \end{bmatrix} \tag{99}$$

For the point model, the FIA (which is an approximation to a model's log evidence) is replaced by the exact evidence, which simply coincides with the log likelihood. For notational consistency, we adopt the following values for the FIA terms:

$$L(X) = \frac{N}{2\sigma^2}\left[(\bar{x}^1)^2 + (\bar{x}^2 - \tau)^2\right] - \frac{N}{2}\ln\left(2\pi\sigma^2\right) \tag{100}$$
$$D = 0 \tag{101}$$
$$B = 0 \tag{102}$$
$$V = 0 \tag{103}$$
$$R = 0 \tag{104}$$

A.4 Numerical experiments with artificial neural networks

A.4.1 Inputs

On each trial, our artificial neural network (henceforth ANN) takes in two images, each depicting one candidate model's location in the data space. It also takes in a length-20 vector, containing the horizontal and vertical coordinates of the $N = 10$ data points. Each image is provided as one RGB matrix of size (3*256*256). In data space units (used for the model definitions in subsection A.3), each image extends from $x = -4$ to $x = 4$ and from $y = -3.5$ to $y = 4.5$, so that the center of the image (located in $(0, 0.5)$) is equidistant from the models in each model pair.

A.4.2 Training dataset

The training dataset consisted of 5000 model pairs. Each model pair was used for generating 50 trials. This approach led to a total of 250000 trials in the entire dataset.

The random generation of model pairs was as follows (see subsection A.3 for the detailed mathematical definitions of each model and the precise meaning of the parameters controlling its shape). Each model pair could be of one of the four variants described in subsection A.2, chosen randomly with equal probabilities. Each model pair could be flipped vertically with probability 0.5. For the robustness variant, the separation of the model pair was 0.6 data space units; for all other model pairs, the separation was 1 data space unit. For the dimensionality variant, the length T (in data space units) of the one-dimensional model was sampled uniformly from $\mathcal{U}(0.5, 5)$. For the boundary variant, the length of both model families were kept identical and sampled from $\mathcal{U}(0.5, 3)$. For the volume variant, the lengths of both models were sampled independently

bioRxiv preprint doi: https://doi.org/10.1101/2023.01.10.523479; this version posted February 8, 2023. The copyright holder for this preprint (which was not certified by peer review) is the author/funder, who has granted bioRxiv a license to display the preprint in perpetuity. It is made available under aCC-BY 4.0 International license.

from $\mathcal{U}(0.5, 5)$; if their length difference was no greater than the task's noise level $\sigma = 1$, then the length of one model was resampled from $\mathcal{U}(0.5, 5)$ until the length difference was greater than 1. For the robustness variant, the length of both model families was kept constant at $(27/50) \cdot \pi$. The length proportion of the rounded model that was perfectly circular was $f = 1/3$, and both model families share the same curvature parameter γ sampled from $\mathcal{U}(1.5, 3)$. The model pairs were centered around the center of each input image.

Given a model pair, each trial was randomly generated as follows. Select one model randomly with equal probability. Sample a location along this model uniformly. Using this location as the center of a 2D isotropic Gaussian and standard deviation of $\sigma = 1$ data space units, sample $N = 10$ data points that were observable to the subject.

The training dataset was pre-shuffled randomly for training purposes. The input batch size was always 50 trials.

A.4.3 Test dataset

The test dataset consisted of 8 model pairs, each generating 15000 trials. Thus, there was a total of 120000 trials in the dataset.

The model pairs were as follows. For the point variant, the one-dimensional model had length (in data space units) 1. For the boundary variant, both model families had length 1. For the volume variant, one model had length 1 while the other had 3. For the robustness variant, both model families had length $T = (27/50) \cdot \pi$, $f = 1/3$, and curvature parameter $\gamma = (3/5)\pi$. Each model pair was presented in the "upright" position (as per the definitions in subsection A.3) and in the vertically flipped position, for a total of 8 cases. The separation between model families and the generation of trials was identical to as in the training dataset.

A.4.4 Artificial neural network architecture

Our ANN had the following architecture (see Figure 3. Each of the two model input images was passed through the pretrained convolutional neural network VGG16, which had its parameters frozen during training. We replaced the fully connected layers at the end of VGG16 with our own structure of Linear-ReLU-BatchNorm1D layers and allowed the updating of weights in these and all subsequent layers. For each image input, the output of this image-processing module was a length-50 vector (model image representation).

In parallel, the length-20 vector of raw data point coordinates was fed through a permutation-invariant layer. This layer featured shared weights such that its outputs were not affected by the sequence of the N=10 data points in the length-20 vector input. This layer also outputted a length-20 vector, which was concatenated to the end of each of the length-50 vectors (the model image representations) along the preexisting dimension, producing two length-70 vectors.

Each length-70 vector was fed through Linear-ReLU-BatchNorm1D layers (identical weights used to process each vector). The resultant two length-50 output vectors were then concatenated together along the preexisting dimension, with the first input image's representation in front.

The resultant length-100 vector was then fed through EquiLinear-ReLU-BatchNorm1D layers. The EquiLinear layers were permutation-equivariant layers of our design, again achieved by weight sharing. They ensure that if we concatenated the two length-50 output vectors in the opposite sequence, then their output, a length-2 vector, also had the same values but in opposite sequence. This length-2 vector was passed through a log softmax layer to produce the ANN's final output, which was also a length-2 vector.

We also introduced a conditional variational encoder (CVAE) structure and used its output as part of the loss function (discussed later), to encourage model representations to preserve information about the data generation process. The details are described below.

We concatenate the length-20 raw data points vector (before passing input the permutation-invariant layers) to the end of each length-50 model image representation vector. The resultant two length-70 vectors (each corresponding to one model) were used as inputs for our CVAE (identical weights used to process each vector). The CVAE took each length-70 vector through its encoder structure to produce 10-dimensional vectors, which were used as parameters $(\mu_{CVAE}, \sigma_{CVAE})$ for the Gaussian random generation of another 10-dimensional vector. The latter vector was again concatenated to the end of the length-50 model image

bioRxiv preprint doi: https://doi.org/10.1101/2023.01.10.523479; this version posted February 8, 2023. The copyright holder for this preprint (which was not certified by peer review) is the author/funder, who has granted bioRxiv a license to display the preprint in perpetuity. It is made available under aCC-BY 4.0 International license.

representation vector responsible for its own generation, before being fed to the CVAE decoder, which mapped back to a 20-dimensional output vector reminiscent of data points. Hence, there were two 20-dimensional output vectors generated, each originating from one model.

A.4.5 Loss function

The loss function for each trial consisted of 2 parts: 1) the final output loss, and 2) the CVAE output loss. For the final output loss, we used Pytorch's negative log likelihood loss function `NLLLoss()`, which computed the loss between the ANN's length-2 output vector and the target label. For each trial's CVAE output loss, we considered only the CVAE output associated with the correct model image/target label (hence one out of the two CVAE output vectors). The CVAE output loss was the sum of a MSE reconstruction loss (between the length-20 CVAE output vector and the length-20 raw data points vector) and a KL Divergence Loss (considering $(\mu_{CVAE}, \sigma_{CVAE})$ used in the CVAE data generation process, using sum reduction). The total loss was the sum of the final output loss and the CVAE output loss.

A.4.6 Update rule

We used Pytorch's Adam optimizer with learning rate 0.005, keeping all other arguments to their default values.

A.4.7 ANN predictions

To evaluate ANN task performance in a way that is comparable to human performance, we need to specify how the ANN output, a length-2 log softmax vector, maps onto a chosen candidate model. The mapping is as follows: we compare the two entries in the output vector and assume that the ANN chooses the candidate model associated with the larger entry.

A.5 Experimental data analysis

For both human and artificial neural network (ANN) experiments, we modeled behavior assuming that each subject samples from a posterior over models determined by a modified version of the Fisher Information Approximation (FIA), where each term of the approximation is multiplied by a free parameter to be inferred, representing the sensitivity of the subject to that term.

Specifically, in our experimental scenario the theory of Bayesian model selection applies directly. Given two models $\mathcal{M}_1$ and $\mathcal{M}_2$, assuming a flat prior over models $p(\mathcal{M}_1) = p(\mathcal{M}_2) = 1/2$ and an uninformative prior (Jeffrey's prior, see Balasubramanian (*11*) and Jaynes (*15*)) over the parameters of each model, when N is sufficiently large we can use the asymptotic expansion in Figure 1 and Equation 33 to write the log posterior ratio for $\mathcal{M}_1$ over $\mathcal{M}_2$ as

$$\begin{aligned}\log \frac{p(\mathcal{M}_1|X)}{p(\mathcal{M}_2|X)} &= \log \frac{p(\mathcal{M}_1|X)}{1-p(\mathcal{M}_1|X)} \\ &\simeq (L_2 - L_1) + (D_2 - D_1) + (B_2 - B_1) + (V_2 - V_1) + (R_2 - R_1)\end{aligned} \tag{105}$$

where L_i, D_i, etc represent the FIA terms for model i:

$$L_i = -\log p(X|\hat{\vartheta}, \mathcal{M}_i) \quad \text{(Likelihood)}$$

$$D_i = \frac{d}{2} \log \frac{N}{2\pi} \quad \text{(Dimensionality)}$$

$$B_i = \frac{1}{2} \log \frac{N}{2\pi} + \log\left[2\pi \left\|\hat{l}\right\|_{\hat{h}^{-1}}\right] \quad \text{(Boundary)}$$

$$V_i = \log \int \mathrm{d}^d\vartheta \sqrt{\det g(\vartheta)} \quad \text{(Volume)}$$

$$R_i = \frac{1}{2} \log\left[\frac{\det h(X;\hat{\vartheta})}{\det g(\hat{\vartheta})}\right] \quad \text{(Robustness)}$$

bioRxiv preprint doi: https://doi.org/10.1101/2023.01.10.523479; this version posted February 8, 2023. The copyright holder for this preprint (which was not certified by peer review) is the author/funder, who has granted bioRxiv a license to display the preprint in perpetuity. It is made available under aCC-BY 4.0 International license.

This expression suggests a simple normative model for subject behavior. Equation 105 determines the probability of reporting $\mathcal{M}_1$ for an ideal Bayesian observer performing probability matching. We can then compare subject behavior to the normative prescription by allowing subjects to have distinct sensitivities to the various terms of the FIA:

$$\begin{aligned}\log\frac{p(\text{report }\mathcal{M}_1|X)}{p(\text{report }\mathcal{M}_2|X)} =& \alpha + \beta_L(L_2 - L_1) + \beta_D(D_2 - D_1) + \\ &+ \beta_B(B_2 - B_1) + \beta_V(V_2 - V_1) + \beta_R(R_2 - R_1)\end{aligned} \tag{106}$$

where α and β were free parameters: α captures any fixed bias, β_L the sensitivity to differences in maximum likelihood, β_D the sensitivity to differences in dimensionality, and so on.

We fitted the model expressed by Equation 106 to subject behavior using a hierarchical, Bayesian logistic regression scheme:

$$\nu_\alpha, \nu_L, \ldots, \nu_R \sim 1 + \text{Exponential}(29) \tag{107}$$
$$\mu_\alpha, \mu_L, \ldots, \mu_R \sim \text{Normal}(0, 3) \tag{108}$$
$$\sigma_\alpha, \sigma_L, \ldots, \sigma_R \sim \text{Exponential}(3) \tag{109}$$
$$\alpha_i \sim \text{StudentT}(\nu_\alpha, \mu_\alpha, \sigma_\alpha) \tag{110}$$
$$\beta_{L,i} \sim \text{StudentT}(\nu_L, \mu_L, \sigma_L) \tag{111}$$
$$\vdots \tag{112}$$
$$\beta_{R,i} \sim \text{StudentT}(\nu_R, \mu_R, \sigma_R) \tag{113}$$
$$C_{i,t} \sim \text{Bernoulli}\left(\text{logit}^{-1}\left(\text{lpr}\left(\alpha_i, \beta_{L,i}, \beta_{D,i}, \beta_{B,i}, \beta_{V,i}, \beta_{R,i}, X_{i,t}\right)\right)\right) \tag{114}$$

where $C_{i,t}$ is the choice made by subject i on trial t, $X_{i,t}$ is the sensory stimulus on that same trial, lpr is the log posterior ratio defined by Equation 106, α_i is the bias for subject i, $\beta_{L,i}$ is the likelihood sensitivity of that same subject, and so on for the other sensitivity parameters. The bias and sensitivity parameters describing each subject are modeled as independent samples from a population-level Student-T probability distribution characterized by a certain shape (ν), location (μ) and scale (σ). The priors assumed over these population-level parameters are standard weakly informative priors (*28*, *29*), and broader or flat priors lead to similar results to those presented below. The model was implemented in PyMC (*49*), and inference was performed by sampling from the posterior for the parameters given the experimental data $\{C_{i,t}, X_{i,t}\}$ using the No-U-Turn Sampler algorithm (*50*, *51*). Further technical details on the inference procedure can be found below, in subsubsection A.5.2.

Definition of relative sensitivity and presentation of sensitivity estimates. Relative sensitivity for a certain feature was defined as the sensitivity for that feature divided by the relevant posterior mean for the likelihood sensitivity. For instance, for dimensionality:

$$\tilde{\beta}_D = \frac{\beta_D}{\langle\beta_L\rangle_{p(\beta_L|\text{data})}} \tag{115}$$

This formulation applies both at the subject level and at the population level.

We note that, because each human subject performed only one task variant, not all sensitivities could be estimated for all subjects. For instance, β_D only entered the behavioral model (and therefore could be estimated) for the subjects that performed the *point* task variant, where the alternative models had different dimensionality. The same holds with β_V and the *horizontal* task variant, and β_R and the *rounded* task variant. The boundary term entered the behavioral model for all task variants, although by design it took on a much broader range of values for the *vertical* task. For consistency, for each sensitivity parameter, we reported its estimate only for those subjects that performed the task variant designed to test it.

bioRxiv preprint doi: https://doi.org/10.1101/2023.01.10.523479; this version posted February 8, 2023. The copyright holder for this preprint (which was not certified by peer review) is the author/funder, who has granted bioRxiv a license to display the preprint in perpetuity. It is made available under aCC-BY 4.0 International license.

A.5.1 Lapse-rate modeling

We designed a variant of the behavioral model that accounts for lapses in subjects' responses (i.e., errors on easy trials). Specifically, we modified Equation 106 as follows:

$$\log \frac{p(\text{report } \mathcal{M}_1|X)}{p(\text{report } \mathcal{M}_2|X)} = \frac{\epsilon}{2} + (1-\epsilon)\big[\alpha + \beta_L(L_2 - L_1) + \beta_D(D_2 - D_1) + \\ + \beta_B(B_2 - B_1) + \beta_V(V_2 - V_1) + \beta_R(R_2 - R_1)\big] \quad (116)$$

where $\epsilon \in [0,1]$ is the lapse rate, representing the probability that a given response is completely random. For $\epsilon = 1$ the responses are random on every trial, whereas for $\epsilon = 0$ this model is equivalent to the original one in Equation 106.

To estimate λ from our experimental data jointly with all other parameters, we kept the same structure as in Equations 107- Equation 114 and exteded it by modeling the population level distribution of ϵ as a Beta distribution, parameterized by count parameters a and b. Following the recommendations in (*29*, section 5.3) and (*52*, section 24.2), we specify hyperpriors in terms of the mean of the distribution $\phi = a/(a+b)$ and the total count $\lambda = a + b$:

$$\phi \sim \text{Uniform}(0,1) \quad (117)$$

$$\lambda \sim \text{Pareto}(0.1, 1.5) \quad \big(p(\lambda) \propto x^{-2.5}\big) \quad (118)$$

$$a = \lambda \cdot \phi \quad (119)$$

$$b = \lambda \cdot (1-\phi) \quad (120)$$

$$\epsilon_i \sim \text{Beta}(a, b) \quad (121)$$

where ϵ_i is the lapse rate for subject i.

A.5.2 Technical details of the inference procedure

Posterior sampling was performed with PyMC (*49*) version 4.2.0, using the NUTS Hamiltonian Monte Carlo algorithm (*50*). Target acceptance probability was set to 0.9 for the human data (both generative and maximum-likelihood task), to 0.8 for the generative task with neural networks and for 0.99 for the maximum-likelihood task for neural networks. The posterior distributions were built by sampling 12 independent Markov chains for 10000 draws each. No divergence occurred in any of the chains. Effective sample size and $\hat{R}$ diagnostics for some of the key parameters are given in Table S5.

A.5.3 Reporting of posterior distributions for inferred parameters

The posterior distributions reported in all figures are Kernel Density Estimates with bandwidth chosen according to Scott's rule (*53*).

bioRxiv preprint doi: https://doi.org/10.1101/2023.01.10.523479; this version posted February 8, 2023. The copyright holder for this preprint (which was not certified by peer review) is the author/funder, who has granted bioRxiv a license to display the preprint in perpetuity. It is made available under aCC-BY 4.0 International license.

B Supplementary Figures and Tables

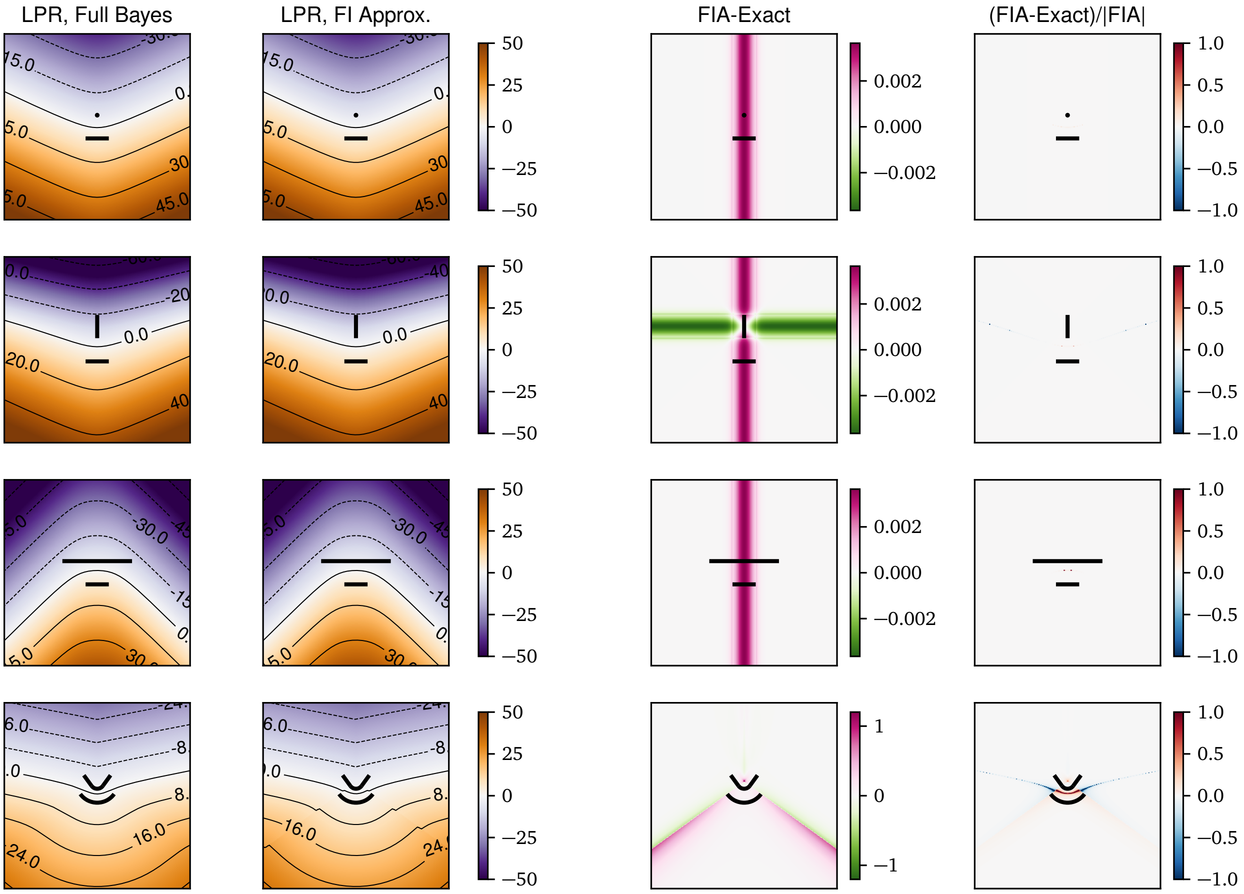

Figure S1: Comparison of the full Bayesian and Fisher Information Approximation computation of the log posterior ratio (LPR) for the model pairs used in our psychophysics tasks ($N = 10$). Each row corresponds to one task variants (from top to bottom, “dimensionality”, “boundary”, “volume”, “robustness”). First column from the left: full Bayesian LPR, computed by numerical integration. Second column: LPR computed with the Fisher Information Approximation. Third column: difference between FIA and exact LPR. Fourth column: relative difference (difference divided by the absolute value of the FIA LPR). Adapted from (*46*).

bioRxiv preprint doi: https://doi.org/10.1101/2023.01.10.523479; this version posted February 8, 2023. The copyright holder for this preprint (which was not certified by peer review) is the author/funder, who has granted bioRxiv a license to display the preprint in perpetuity. It is made available under aCC-BY 4.0 International license.

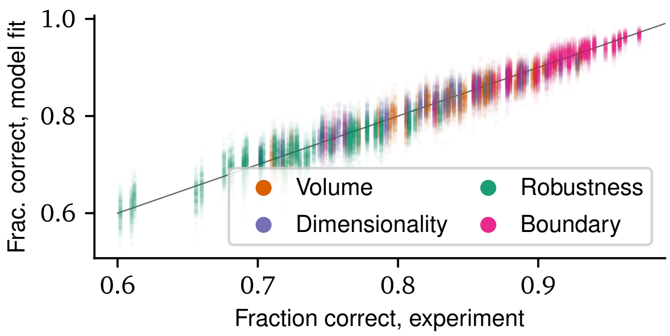

Figure S2: Posterior predictive check for the human subjects in the generative task, looking at subject performance. We sampled 240 samples from the posterior over model parameters by thinning the MCMC chains used for model inference. For each of these samples, we ran a simulation of the experiment using the actual stimuli shown to the subjects, and we recorded the resulting performance of all 202 simulated subjects. This procedure yielded 240 samples of the joint posterior-predictive distribution of task performance over all experimental subjects. To visualize this distribution, for each subject we plotted a cloud of 240 dots where the y coordinate of each dot is the simulated performance of that subject in one of the simulations, and the x coordinate is the true performance of that subject in the experiment plus a small random jitter (for ease of visualization). The gray line is the identity, showing that our inference procedure captures well the behavioral patterns in the experimental data. In the figure, all task types are pooled together, but subjects that performed different task types are distinguished by the color of the dots.

bioRxiv preprint doi: https://doi.org/10.1101/2023.01.10.523479; this version posted February 8, 2023. The copyright holder for this preprint (which was not certified by peer review) is the author/funder, who has granted bioRxiv a license to display the preprint in perpetuity. It is made available under aCC-BY 4.0 International license.

	Orig. task		Max lik. task	
Parameter	Humans	ANNs	Humans	ANNs
μ_α (up/down bias)	0.107 ± 0.023	-0.242 ± 0.151	0.056 ± 0.024	0.010 ± 0.07
μ_L (likelihood)	0.461 ± 0.012	6.529 ± 0.188	0.561 ± 0.018	7.966 ± 0.401
μ_D (dimensionality)	2.150 ± 0.445	8.484 ± 0.653	1.285 ± 0.231	-0.030 ± 0.486
μ_B (boundary)	0.518 ± 0.045	6.286 ± 0.186	0.499 ± 0.058	0.883 ± 0.121
μ_V (volume)	0.108 ± 0.057	6.089 ± 0.204	0.105 ± 0.044	0.128 ± 0.196
μ_R (robustness)	1.018 ± 0.056	4.882 ± 0.417	1.276 ± 0.085	0.356 ± 0.281

Table S1: Posterior mean±standard deviation for population-level parameters. See Equation 106 to Equation 114 for the precise definition of each parameter and its role in the hierarchical model of behavior.

bioRxiv preprint doi: https://doi.org/10.1101/2023.01.10.523479; this version posted February 8, 2023. The copyright holder for this preprint (which was not certified by peer review) is the author/funder, who has granted bioRxiv a license to display the preprint in perpetuity. It is made available under aCC-BY 4.0 International license.

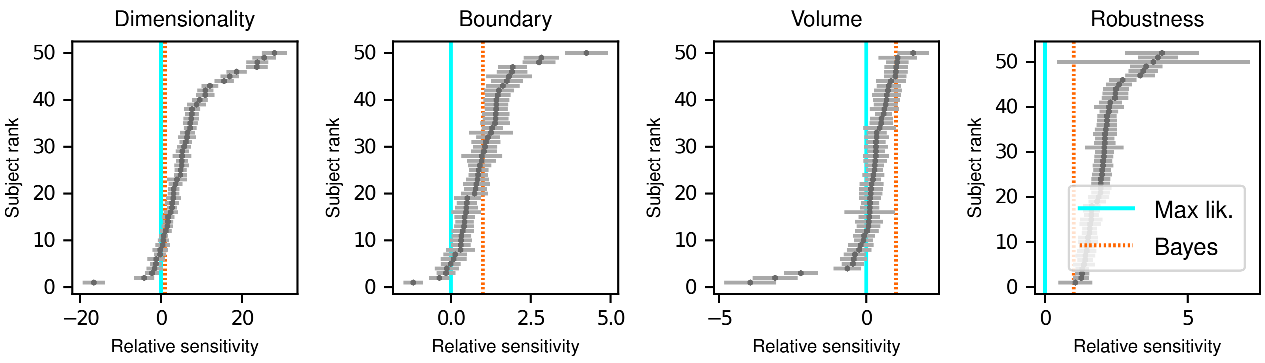

Figure S3: Subject-level relative sensitivities to the geometric features that determine model complexity. Dots with error bars: posterior mean ± standard deviation of the relative sensitivity (the dots are the same as in Figure 2c). For ease of visualization, subjects are ranked based on their posterior mean.

bioRxiv preprint doi: https://doi.org/10.1101/2023.01.10.523479; this version posted February 8, 2023. The copyright holder for this preprint (which was not certified by peer review) is the author/funder, who has granted bioRxiv a license to display the preprint in perpetuity. It is made available under aCC-BY 4.0 International license.

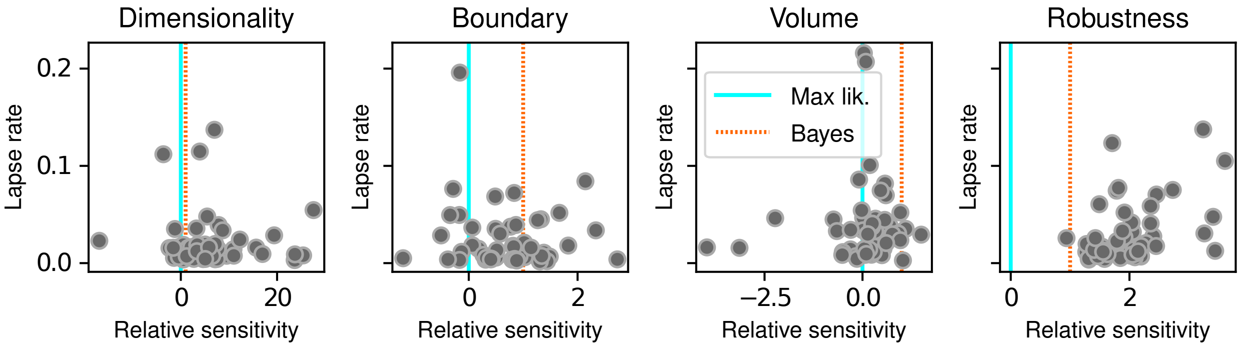

Figure S4: Lapse rate versus relative sensitivity to complexity across subjects. Each dot gives the posterior mean estimate of the relative sensitivity to one of the features that determine model complexity (abscissa) and the posterior mean estimate of the lapse rate, as defined in Section A.5.1.

bioRxiv preprint doi: https://doi.org/10.1101/2023.01.10.523479; this version posted February 8, 2023. The copyright holder for this preprint (which was not certified by peer review) is the author/funder, who has granted bioRxiv a license to display the preprint in perpetuity. It is made available under aCC-BY 4.0 International license.

Model	Rank	WAIC	pWAIC	dWAIC	SE	dSE
Full	0	-34824.6	641.963	0	183.981	0
Likelihood only	1	-37524.9	370.340	2700.4	183.923	69.3817

Table S2: WAIC comparison of the full model and the likelihood-only model for the human subjects in the generative task, reported in the standard format used by (*27*, section 6.4.2). Briefly, WAIC is the value of the criterion (log-score scale — higher is better); pWAIC is the estimated effective number of parameters; dWAIC is the difference between the WAIC of the given model and the highest-ranked one; SE is the standard error of the WAIC estimate; and dSE is the standard error of the difference in WAIC. These estimates were produced with the `compare` function provided by ArviZ (*54*), using 12 MCMC chains with 10000 samples each for each model (in total, 120000 samples for each model).

bioRxiv preprint doi: https://doi.org/10.1101/2023.01.10.523479; this version posted February 8, 2023. The copyright holder for this preprint (which was not certified by peer review) is the author/funder, who has granted bioRxiv a license to display the preprint in perpetuity. It is made available under aCC-BY 4.0 International license.

Model	Rank	WAIC	pWAIC	dWAIC	SE	dSE
Full	0	-31022.8	638.926	0	184.912	0
Likelihood only	1	-33155.1	374.023	2132.28	186.667	63.1851

Table S3: Same as Table S2, for the maximum-likelihood task, where subjects were asked to report the model that was closest to the data.

bioRxiv preprint doi: https://doi.org/10.1101/2023.01.10.523479; this version posted February 8, 2023. The copyright holder for this preprint (which was not certified by peer review) is the author/funder, who has granted bioRxiv a license to display the preprint in perpetuity. It is made available under aCC-BY 4.0 International license.

Parameter	ROPE	95% HDI (generative)	PD (generative)	95% HDI (max lik.)	PD (max lik.)
Likelihood	$\|\beta_L\| < 0.0076$	$[0.012, 0.437]$	1.00	$[0.526, 0.597]$	1.00
Dimensionality	$\|\beta_D\| < 0.43$	$[1.299, 3.048]$	1.00	$[0.835, 1.745]$	1.00
Boundary	$\|\beta_B\| < 0.06$	$[0.43, 0.604]$	1.00	$[0.386, 0.612]$	1.00
Volume	$\|\beta_V\| < 0.091$	$[-0.005, 0.218]$	0.97	$[0.019, 0.193]$	0.99
Robustness	$\|\beta_R\| < 0.11$	$[0.908, 1.126]$	1.00	$[1.11, 1.446]$	1.00

Table S4: HDI vs ROPE comparison and Probability of Direction (PD) for the population-level parameters in the human experiments. See Supplementary Information section C.7.2 and (*28*) for an explanation of the ROPE-HDI comparison, and (*55*, *56*) for more details on the probability of direction metric. Note that the ROPE and HDI definitions were preregistered (*24*–*26*).

bioRxiv preprint doi: https://doi.org/10.1101/2023.01.10.523479; this version posted February 8, 2023. The copyright holder for this preprint (which was not certified by peer review) is the author/funder, who has granted bioRxiv a license to display the preprint in perpetuity. It is made available under aCC-BY 4.0 International license.

Parameter	ESS	$\hat{R}$
μ_α	52148	1.000
μ_L	19254	1.000
μ_D	27384	1.000
μ_B	32434	1.000
μ_V	64614	1.000
μ_R	112118	1.000

Table S5: $\hat{R}$ statistic and effective sample size (ESS) for 12 Markov Chain traces run as described in the text, for the fit to human data in the generative task. See (*29*, sections 11.4–11.5) and Vehtari *et al.* (*57*) for in-depth discussion of chain quality diagnostics. Briefly, $\hat{R}$ depends on the relationship between the variance of the draws estimated within and between contiguous draw sequences. $\hat{R}$ is close to 1 when the chains have successfully converged. The effective sample size estimates how many independent samples one would need to extract the same amount of information as that contained in the (correlated) MCMC draws.

bioRxiv preprint doi: https://doi.org/10.1101/2023.01.10.523479; this version posted February 8, 2023. The copyright holder for this preprint (which was not certified by peer review) is the author/funder, who has granted bioRxiv a license to display the preprint in perpetuity. It is made available under aCC-BY 4.0 International license.

C Supplementary Text

C.1 Numerical comparison of the extended FIA vs exact Bayes

Figure S1 shows that the FIA computed with the expressions given in this document provides a very good approximation to the exact Bayesian log posterior ratio (LPR) for the model pairs used in the psychophysics experiments, and for the chosen sample size ($N = 10$). As highlighted in the panels in the rightmost column, the discrepancies between the exact and the approximated LPR are generally small in relative terms, and therefore are not very important for the purpose of model fitting and interpretation. Note that here, as well as for the results in the main text, the B term in the FIA is computed using Equation 34 rather than Equation 38 in order to avoid infinities (that for finite N can arise when the likelihood gradient is very small) and discontinuities (that for finite N can arise on the interior of the manifold, in proximity to the boundary, where the value of B goes from zero when $\hat{\vartheta}$ is in the interior to $\log(2)$ when $\hat{\vartheta}$ is exactly on the boundary).

Even though overall the agreement between the approximation is good, it is interesting to look more closely at where the approximation is poorest. The task type for which the discrepancies are the largest (both in absolute and relative terms) is the "robustness" type (fourth row in Figure S1). This discrepancy arises because the FIA hypotheses are not fully satisfied everywhere for one of the models. More specifically, the models in that task variant are a circular arc (the bottom model in Figure S1, third row) and a smaller circular arc, concentric with the first, with a straight segment attached to either side (the top model). The log-likelihood function for this second model is smooth only to first order, but its second derivative (and therefore its Fisher Information and its observed Fisher Information) is not continuous at the points where the circular arc is joined with the straight segments, locally breaking hypothesis number 3 in subsubsection A.1.1. Geometrically, this discontinuity is analogous to saying that the curvature of the manifold changes abruptly at the joints. It is likely that the FIA for a model with a smoother transition between the circular arc and the straight arms would have been even closer to the exact value for all points on the 2D plane (the data space). More generally, this line of reasoning suggests that it would be interesting to investigate the features of a model that affect the quality of the Fisher Information Approximation.

C.2 Subject demographics

For the generative task, after excluding subjects based on pre-registered performance thresholds as detailed above, we were left with data from 202 subjects. The age range was 18–68 (mean: 28.0, std: 9.5). Sex was reported as male for 129 subjects, female for 69, other for 4. Ethnicity was as follows: White 174, Asian 12, Hispanic or Latino 5, Other 4, Black or African American 3, American Indian or Alaskan Native 2, do not wish to respond 2. For the maximum-likelihood task, the total number of subjects after exclusions was 201, and the with the following demographics. Age: 18–87 (mean: 35.9, std: 12.8). Sex: male 124, female 75, other 1, prefer not to say 1. Ethnicity: White 157, Asian 25, Black or African American 8, Other 6, Hispanic or Latino 3, American Indian or Alaskan Native 1, do not wish to respond 1.

C.3 Posterior predictive checks

We performed a simple posterior predictive check (*28*) to ensure that the Bayesian hierarchical model described in the text captures the main pattern of behavior across our subjects. In Figure S2, the behavioral performance of the subjects is compared with its posterior predictive distribution under the model, for the case of the human subjects in the generative task. As can be seen from the figure, the performance of each subject is correctly captured by the model, across systematic differences between task types (with subjects performing better in the boundary task variant than the robustness task variant, for instance) as well as individual differences between subjects that performed the same task variant.

C.4 Details on raw estimated sensitivities

Table S1 reports the posterior mean and standard deviation of the population-level parameters entering the regression (Equation 106). Note that these are the raw parameters, not their normalized counterparts relative to the likelihood sensitivity as reported in the rest of the paper.

bioRxiv preprint doi: https://doi.org/10.1101/2023.01.10.523479; this version posted February 8, 2023. The copyright holder for this preprint (which was not certified by peer review) is the author/funder, who has granted bioRxiv a license to display the preprint in perpetuity. It is made available under aCC-BY 4.0 International license.

C.5 Uncertainty in subject-level sensitivities

Figure S3 illustrates the uncertainty in the estimate for the relative sensitivity of each subject. This uncertainty is typically small compared to between-subject variability of the sensitivity, which is therefore not a trivial consequence of the noise in the sensitivity estimation for individual subjects.

C.6 Lapse-rate analysis

By applying the model variant described in Section A.5.1, we were able to estimate a lapse rate for each subject simultaneously with the sensitivity parameters. The results are summarized in Figure S4, showing that although there is a substantial spread of lapse rates in the range 0–0.2, there is no clear relationship between lapse rates and sensitivity. The sensitivity parameters estimated with this extended model were qualitatively compatible with those presented everywhere else in the text.

C.7 Outcome of significance tests specified in the preregistration documents

C.7.1 Formal comparison between ideal observers

We compared the Bayesian hierarchical model described in section A.5 to a simpler model, where subjects were assumed to only be sensitive to likelihood differences, or in other words to choose $\mathcal{M}_1$ over $\mathcal{M}_2$ based only on which model was on average closer to the dot cloud constituting the stimulus on a given trial. Mathematically, this "likelihood-only" model was equivalent to fixing all β parameters to zero except for β_L in the model described in section A.5. All other details of the model were the same, and in particular the model still had a hierarchical structure with adaptive shrinkage (the subject-level parameters α and β_L were modeled as coming from Student T distributions controlled by population-level parameters). We compared the full model and the likelihood-only model on our human behavior data using the Widely Applicable Information Criterion (*29*). This comparison indicates strong evidence in favor of the full model not only in the generative task (Table S2), but also in the maximum-likelihood task (Table S3).

C.7.2 Other statistical tests

As described in the preregistration documents (*24–26*), in this work we have emphasized parameter estimation and information criteria-based model comparison over null hypothesis significance testing (see for instance (*27*), and (*28*) for a discussion and comparison of these ideas). However, for completeness, we report in Table S4 (1) the comparison between the Regions of Practical Equivalence (ROPE, (*28*)) and the 95% highest-density interval (HDI) for each population-level parameter, and (2) the "probability of direction" (*55*, *56*) for the same parameters (see below for more details on these methods). The ROPE-HDI tests highlight that the null value of zero sensitivity is not credible (rejected) for L, D and R, and neither rejected not accepted for V. The probability of direction is high for all parameters, including V, which has $PD = 0.97$ for the generative task and $PD = 0.99$ for the maximum-likelihood task. Overall, these analyses point to a significant sensitivity for all terms of the FIA in both experiments (generative and maximum-likelihood), with V having a more moderate effect size than the other terms.

Technical details on the ROPE-HDI comparison and on the Probability of Direction for sensitivity parameters Briefly, the ROPE for a parameter is the range around a null value for that parameter such that variations within this range would imply only a "negligible change" in the behavior of the model, if all other parameters were held at their null values. The HDI is the smallest interval that contains a certain probability mass for the posterior of that parameter. The ROPE-HDI comparison is based on the idea that if the bulk of the posterior distribution for that parameter (represented by the HDI) falls outside the ROPE, then the null value for that parameter can be considered not credible (rejected). On the other hand, if the bulk of the posterior for the parameter falls within the ROPE, the null value can be considered credible (accepted). Finally, if the posterior distribution has a partial overlap with the ROPE (neither mostly contained within it, nor mostly falling outside of it), then the test is inconclusive. Note that, just like frequentist null hypothesis significance testing procedures and unlike the information criterion approach used above, this

bioRxiv preprint doi: https://doi.org/10.1101/2023.01.10.523479; this version posted February 8, 2023. The copyright holder for this preprint (which was not certified by peer review) is the author/funder, who has granted bioRxiv a license to display the preprint in perpetuity. It is made available under aCC-BY 4.0 International license.

method depends on some arbitrary assumptions, namely the definition of the ROPE and the probability to use in computing the HDI.

In practice, as explained in more details in our preregistration documents (*24–26*), here we define, conventionally, the HDI as the smallest interval that contains 95% of the posterior. The ROPE is computed as follows. We start by defining a "negligible change" over the probability of the choice variable over the "main range" $[\mu_x - 2\sigma_x, \mu_x + 2\sigma_x]$ of one of the predictors in our model (L, D, B, V or R). In other words pick an interval of probabilities $[\pi_0 - \delta, \pi_0 + \delta]$ such that if the probability stays within $[\pi_0 - \delta, \pi_0 + \delta]$ when x varies over its typical range, then the probability is not meaningfully affected by x. Mathematically, if the probability of choosing one of the alternatives in the task is π and the log-odds is $\text{logit}(\pi) = \log(\pi/(1-\pi)) =$, then in a logistic regression setting

$$\text{logit}(\pi) = \alpha + \beta x \quad . \tag{122}$$

If $\pi_0 = \text{logit}^{-1}(\alpha)$, then the ROPE for β is defined as

$$\text{logit}(\pi_0 + \delta) = \alpha + \beta_+(\mu_x + 2\sigma_x) \tag{123}$$
$$\text{logit}(\pi_0 - \delta) = \alpha + \beta_-(\mu_x - 2\sigma_x) \tag{124}$$

so that

$$\beta_+ = \frac{\text{logit}(\pi_0 + \delta) - \alpha}{\mu_x + 2\sigma_x} \tag{125}$$
$$\beta_- = \frac{\text{logit}(\pi_0 - \delta) - \alpha}{\mu_x - 2\sigma_x} \tag{126}$$

In our case, assuming a negligible influence of the up/down bias (α in Equation 106), $\pi_0 = 0.5$ and therefore we can assume $\alpha = 0$. The definition of the ROPE further depends on the arbitrary choice of δ, and on the values of μ_x and σ_x. We choose $\delta = 0.025$, and we estimate μ_x and σ_x by generating 25000 experimental trials per task type (Dimensionality, Boundary, Volume, Robustness) and computing the empirical average and standard deviation of the predictors over that trial set. These numbers were all fixed at preregistration time (*24–26*).

References

11. V. Balasubramanian, Statistical Inference, Occam's Razor, and Statistical Mechanics on the Space of Probability Distributions. *Neural Computation* **9**, 349–368 (1997).

15. E. T. Jaynes, *Probability Theory: The Logic of Science* (Cambridge University Press, Apr. 1, 2003), 753 pp., ISBN: 0-521-59271-2.

24. E. Piasini, V. Balasubramanian, J. I. Gold, *Preregistration Document* (https://doi.org/10.17605/OSF.IO/2X9H6).

25. E. Piasini, V. Balasubramanian, J. I. Gold, *Preregistration Document Addendum* (https://doi.org/10.17605/OSF.IO/5HDQZ).

26. E. Piasini, S. Liu, V. Balasubramanian, J. I. Gold, *Preregistration Document Addendum* (https://doi.org/10.17605/OSF.IO/826JV).

27. R. McElreath, *Statistical Rethinking* (CRC Press, 2016), ISBN: 978-1-4822-5344-3.

28. J. K. Kruschke, *Doing Bayesian Data Analysis* (Academic Press, ed. 2, 2015), ISBN: 978-0-12-405888-0.

29. A. Gelman *et al.*, *Bayesian Data Analysis* (CRC Press, ed. 3, 2014), ISBN: 978-1-4398-4095-5.

46. E. Piasini, V. Balasubramanian, J. I. Gold, presented at the The First International Symposium on AI and Neuroscience - ACAIN 2021.

47. M. Abramowitz, I. A. Stegun, *Handbook of Mathematical Functions: With Formulas, Graphs, and Mathematical Tables* (Dover, New York, 1972), ISBN: 0-486-61272-4.

bioRxiv preprint doi: https://doi.org/10.1101/2023.01.10.523479; this version posted February 8, 2023. The copyright holder for this preprint (which was not certified by peer review) is the author/funder, who has granted bioRxiv a license to display the preprint in perpetuity. It is made available under aCC-BY 4.0 International license.

48. S.-i. Amari, H. Nagaoka, *Methods of Information Geometry*, trans. by D. Harada (American Mathematical Society, 2000), 206 pp., ISBN: 0-8218-4302-8.

49. J. Salvatier, T. V. Wiecki, C. Fonnesbeck, Probabilistic Programming in Python Using PyMC3. *PeerJ Computer Science* **2**, e55 (Apr. 2016).

50. M. D. Hoffman, A. Gelman, The No-U-Turn Sampler: Adaptively Setting Path Lengths in Hamiltonian Monte Carlo. *Journal of Machine Learning Research* **15**, 1593–1623, (`http://jmlr.org/papers/v15/hoffman14a.html`) (2014).

51. M. Betancourt, *A Conceptual Introduction to Hamiltonian Monte Carlo*

52. S. development team, *Stan Modeling Language Users Guide, Version 2.31*.

53. F. Pedregosa *et al.*, Scikit-Learn: Machine Learning in Python. *Journal of Machine Learning Research* **12**, 2825–2830, ISSN: 1533-7928, (2023; `http://jmlr.org/papers/v12/pedregosa11a.html`) (2011).

54. R. Kumar, C. Carroll, A. Hartikainen, O. Martin, ArviZ a Unified Library for Exploratory Analysis of Bayesian Models in Python. *Journal of Open Source Software* **4**, 1143 (2019).

55. D. Makowski, M. S. Ben-Shachar, D. Lüdecke, bayestestR: Describing Effects and Their Uncertainty, Existence and Significance within the Bayesian Framework. *Journal of Open Source Software* **4**, 1541, ISSN: 2475-9066, (2022; `https://joss.theoj.org/papers/10.21105/joss.01541`) (Aug. 13, 2019).

56. D. Makowski, M. S. Ben-Shachar, S. H. A. Chen, D. Lüdecke, Indices of Effect Existence and Significance in the Bayesian Framework. *Frontiers in Psychology* **10**, ISSN: 1664-1078, (2022; `https://www.frontiersin.org/articles/10.3389/fpsyg.2019.02767`) (2019).

57. A. Vehtari, A. Gelman, D. Simpson, B. Carpenter, P.-C. Bürkner, Rank-Normalization, Folding, and Localization: An Improved $\hatR$ for Assessing Convergence of MCMC. *Bayesian Analysis* (2020).
